# Differential diagnosis of neurodegenerative dementias with the explainable MRI based machine learning algorithm MUQUBIA

**DOI:** 10.1038/s41598-023-43706-6

**Published:** 2023-10-13

**Authors:** Silvia De Francesco, Claudio Crema, Damiano Archetti, Cristina Muscio, Robert I. Reid, Anna Nigri, Maria Grazia Bruzzone, Fabrizio Tagliavini, Raffaele Lodi, Egidio D’Angelo, Brad Boeve, Kejal Kantarci, Michael Firbank, John-Paul Taylor, Pietro Tiraboschi, Alberto Redolfi, the RIN – Neuroimaging Network, Maria Grazia Bruzzone, Pietro Tiraboschi, Claudia A. M. Gandini Wheeler-Kingshott, Michela Tosetti, Gianluigi Forloni, Alberto Redolfi, Egidio D’Angelo, Fabrizio Tagliavini, Raffaele Lodi, Raffaele Agati, Marco Aiello, Elisa Alberici, Carmelo Amato, Domenico Aquino, Filippo Arrigoni, Francesca Baglio, Laura Biagi, Lilla Bonanno, Paolo Bosco, Francesca Bottino, Marco Bozzali, Nicola Canessa, Chiara Carducci, Irene Carne, Lorenzo Carnevale, Antonella Castellano, Carlo Cavaliere, Mattia Colnaghi, Valeria Elisa Contarino, Giorgio Conte, Mauro Costagli, Greta Demichelis, Silvia De Francesco, Andrea Falini, Stefania Ferraro, Giulio Ferrazzi, Lorenzo Figà Talamanca, Cira Fundarò, Simona Gaudino, Francesco Ghielmetti, Ruben Gianeri, Giovanni Giulietti, Marco Grimaldi, Antonella Iadanza, Matilde Inglese, Maria Marcella Laganà, Marta Lancione, Fabrizio Levrero, Daniela Longo, Giulia Lucignani, Martina Lucignani, Maria Luisa Malosio, Vittorio Manzo, Silvia Marino, Jean Paul Medina, Edoardo Micotti, Claudia Morelli, Cristina Muscio, Antonio Napolitano, Anna Nigri, Francesco Padelli, Fulvia Palesi, Patrizia Pantano, Chiara Parrillo, Luigi Pavone, Denis Peruzzo, Nikolaos Petsas, Anna Pichiecchio, Alice Pirastru, Letterio S. Politi, Luca Roccatagliata, Elisa Rognone, Andrea Rossi, Maria Camilla Rossi-Espagnet, Claudia Ruvolo, Marco Salvatore, Giovanni Savini, Emanuela Tagliente, Claudia Testa, Caterina Tonon, Domenico Tortora, Fabio Maria Triulzi

**Affiliations:** 1 Laboratory of Neuroinformatics, IRCCS Istituto Centro San Giovanni di Dio Fatebenefratelli, Brescia, Italy; 2 ASST Bergamo Ovest, Bergamo, Italy; 3 Division of Neurology V/Neuropathology, Fondazione IRCCS Istituto Neurologico Carlo Besta, Milan, Italy; 4 Department of Information Technology, Mayo Clinic and Foundation, Rochester, Minnesota USA; 5 Department of Neuroradiology, Fondazione IRCCS Istituto Neurologico Carlo Besta, Milan, Italy; 6 Scientific Directorate, Fondazione IRCCS Istituto Neurologico Carlo Besta, Milan, Italy; 7 Department of Biomedical and Neuromotor Sciences, University of Bologna, Bologna, Italy; 8 IRCCS Istituto delle Scienze Neurologiche di Bologna, Bologna, Italy; 9 Department of Brain and Behavioral Sciences, University of Pavia, Pavia, Italy; 10 IRCCS Mondino Foundation, Pavia, Italy; 11 Department of Neurology, Mayo Clinic, Rochester, Minnesota USA; 12 Department of Radiology, Mayo Clinic, Rochester, Minnesota USA; 13 Translational and Clinical Research Institute, Newcastle University, Campus for Ageing and Vitality, Newcastle Upon Tyne, UK; 14 Queen Square MS Center, Department of Neuroinflammation, UCL Institute of Neurology, London, UK; 15 University of Pavia, Pavia, Italy; 16 IRCCS Stella Maris, Pisa, Italy; 17 Istituto di Ricerche Farmacologiche Mario Negri IRCCS, Milan, Italy; 18 IRCCS SYNLAB SDN, Naples, Italy; 19 IRCCS Istituti Clinici Scientifici Maugeri, Pavia, Italy; 20 Oasi Research Institute-IRCCS, Troina, Italy; 21 Scientific Institute, IRCCS E. Medea, Milan, Italy; 22 Fondazione IRCCS Istituto Neurologico Carlo Besta, Milan, Italy; 23 IRCCS Fondazione Don Carlo Gnocchi ONLUS, Milan, Italy; 24 IRCCS Centro Neurolesi Bonino Pulejo, Messina, Italy; 25 IRCCS Istituto Ospedale Pediatrico Bambino Gesù, Rome, Italy; 26 Fondazione IRCCS Santa Lucia, Rome, Italy; 27 IRCCS Neuromed, Pozzilli, Italy; 28 IRCCS Ospedale San Raffaele, Milan, Italy; 29 IRCCS Istituto Auxologico Italiano, Milan, Italy; 30 Fondazione IRCCS Ca’ Granda Osp. Maggiore Policlinico, Milan, Italy; 31 University of Genoa, Genoa, Italy; 32 Philips Healthcare, Milan, Italy; 33 Fondazione Policlinico Universitario Agostino Gemelli IRCCS, Rome, Italy; 34 IRCCS Humanitas Research Hospital, Rozzano, Italy; 35 IRCCS Ospedale Policlinico San Martino, Genoa, Italy; 36 IRCCS Istituto Giannina Gaslini, Genoa, Italy; 37 University of Turin, Turin, Italy

**Keywords:** Information technology, Software, Neurology, Dementia, Neurodegenerative diseases

## Abstract

Biomarker-based differential diagnosis of the most common forms of dementia is becoming increasingly important. Machine learning (ML) may be able to address this challenge. The aim of this study was to develop and interpret a ML algorithm capable of differentiating Alzheimer’s dementia, frontotemporal dementia, dementia with Lewy bodies and cognitively normal control subjects based on sociodemographic, clinical, and magnetic resonance imaging (MRI) variables. 506 subjects from 5 databases were included. MRI images were processed with FreeSurfer, LPA, and TRACULA to obtain brain volumes and thicknesses, white matter lesions and diffusion metrics. MRI metrics were used in conjunction with clinical and demographic data to perform differential diagnosis based on a Support Vector Machine model called MUQUBIA (Multimodal Quantification of Brain whIte matter biomArkers). Age, gender, Clinical Dementia Rating (CDR) Dementia Staging Instrument, and 19 imaging features formed the best set of discriminative features. The predictive model performed with an overall Area Under the Curve of 98%, high overall precision (88%), recall (88%), and F1 scores (88%) in the test group, and good Label Ranking Average Precision score (0.95) in a subset of neuropathologically assessed patients. The results of MUQUBIA were explained by the SHapley Additive exPlanations (SHAP) method. The MUQUBIA algorithm successfully classified various dementias with good performance using cost-effective clinical and MRI information, and with independent validation, has the potential to assist physicians in their clinical diagnosis.

## Introduction

Neurodegenerative dementias are a common and increasing cause of mortality and disability worldwide, particularly in older age^1^. The most common form of neurodegenerative dementia worldwide is Alzheimer’s dementia (AD), but recent epidemiological studies and refinement of new clinical criteria have shown that frontotemporal dementia (FTD) and dementia with Lewy bodies (DLB) are also common forms^2^. Specifically, DLB accounts for 5–7% of all dementias in the elderly^3^, FTD about 7%^4^, with one in four cases occurring late in life^5^, while AD may contribute to 60–70% of cases overall^6^. These neurodegenerative dementias are heterogeneous in their clinical presentation and underlying pathophysiology, although they share overlapping features^7^.

Biomarkers provide a powerful approach to understand the spectrum of neurological diseases by identifying them from the earliest manifestations to the final stages^8^. Increased diagnostic accuracy allows more precise prognostic approaches and often leads to specific treatments and optimal patient care^9^. In this context, it is important to determine which diagnostic markers can most reliably identify the different pathologies that lead to dementia. The main challenge for researchers and clinicians is to determine biomarkers that not only identify AD but can simultaneously distinguish between patients with FTD, DLB and cognitively normal controls (CN). Currently, imaging biomarkers assessed by magnetic resonance imaging (MRI) in conjunction with clinical examinations and neurocognitive assessments are the most commonly used tests to diagnose neurodegenerative dementias^10^. In recent years, several MRI-based imaging sequences or modalities have been introduced into clinical practice. The most commonly used MRI sequences are: structural T1-weighted 3D (T13D) and T2 Fluid Attenuated Inversion Recovery (FLAIR) images, which provide morphological measurements of the brain. In addition, Diffusion Tensor Imaging (DTI) is a well-established technique that is particularly useful for studying white matter (WM) integrity^11^.

The development of accurate image analysis pipelines combined with advanced classification methods could improve differential diagnosis^12^. Indeed, automated MRI segmentation tools can systematically generate brain morphometric features with minimal operator-differences, although a limitation is that some of these tools require a lot of processing time and computational power.

The best-known segmentation algorithms are *FreeSurfer* (FS), which can extract volume, area and thickness of many brain regions of interest (ROI) and the *Lesion Prediction Algorithm* (LPA), which can quantify WM hyperintensities. Both algorithms have been validated against manual raters and performed well^13–15^. As for DTI analysis, *TRActs Constrained by UnderLying Anatomy* (TRACULA) is one of the best validated tools for reconstructing WM pathways^16^.

The results of automated MRI pipelines can be used to develop machine learning (ML) tools with good classification performance. Support Vector Machines (SVM) are among the widely used supervised ML algorithms because they are easy to implement while being effective in diagnostic classification tasks^17,18^. In some cases, imaging variables can be used in conjunction with clinical and neuropsychological variables as input to multivariate data analyses and ML algorithms^19^. These models have been shown to be an effective strategy for identifying features capable of discriminating between different classes and subtypes of disease^20,21^, with results comparable to or better than neuropsychological tests alone^22,23^.

Indeed, ML in neuroscience is an ever-growing area of research based on learning relationships from large and complex data sets with the ability to apply the learned rules to other similar unseen data. Often, these tools appear to be able to detect brain patterns that are beyond human perception and can help clinicians to highlight and interpret medical findings^24^. To this end, tools for global and local interpretability of ML models have recently been developed^25^.

The present study was conducted within the framework of the Italian Network for Neuroscience and Neurorehabilitation (RIN) (https://www.reteneuroscienze.it/en/), established in 2017 by the Italian Ministry of Health. The RIN (1) promotes collaboration among the National Research Hospitals (IRCCS), (2) facilitates the dissemination of information on clinical/scientific community, and (3) promotes the use of harmonized protocols and advanced ML tools to enhance clinical practice^26–28^.

With this background, we developed and explained how our ML algorithm classified subjects into the four diagnostic classes (i.e.: AD, FTD, DLB, CN) based on sociodemographic, clinical, and imaging data. Our objectives were to: (1) discover the most informative combination of biomarkers to distinguish the different forms of dementia; (2) investigate the pathophysiological role of WM alterations multimodally; (3) provide an interpretation of how MUltimodal QUantification of Brain whIte matter biomArkers in dementia (MUQUBIA) works.

## Methods

### Study design

This study included the following steps: data preprocessing, selection of discriminative features, classification of subjects, SHapley Additive exPlanations (SHAP) analyses.

MRI images were processed with automated tools to extract the volume and thickness of cortical and subcortical brain regions, WM lesions, and WM diffusion metrics. All these values were used to train and test the MUQUBIA model for classification into diagnostic groups with a hold-out strategy.

### Data

Subjects with a clinical diagnosis of AD, FTD, DLB, or CN were selected from 5 data sets.

The databases used for data collection were:

- Alzheimer’s Disease Neuroimaging Initiative (ADNI)^29^: 84 AD, 15 DLB (from Neuropathology Data, http://adni.loni.usc.edu/methods/neuropath-methods/), 80 CN;
- Frontotemporal Lobar Degeneration Neuroimaging Initiative (FTLDNI): 135 FTD, 10 CN;
- National Alzheimer's Coordinating Center (NACC)^30^: 26 AD, 27 DLB, 18 CN;
- NIH Parkinson's Disease Biomarkers Program (PDBP)^31^: 60 DLB;
- Newcastle University, Newcastle upon Tyne^32–35^: 51 DLB.

The FTLDNI database contained sufficient FTD data for our purposes. All three FTD subtypes (i.e.: behavioural variant, semantic variant, and progressive non-fluent aphasia) were considered. AD and CN were selected from a larger sample to avoid size imbalance. For these three classes, only subjects with all three available sequences at the same time-point and DTI directions greater than 12 were included. Because there were no available open access databases of DLB patients with all three sequences needed for this study, we also included subjects with at least one sequence for the DLB group (Supplementary Table S2), thus improving the sample size and allowing more accurate data imputation. A sample of no less than 100 subjects was assembled for each diagnostic class. Sociodemographic, clinical, and imaging variables were collected for all subjects. Neuropsychological test scores were collected in our study but not included in the analysis because the assessment protocol for CN does not always include neuropsychological characterization. The clinical assessment used was the global score of the Clinical Dementia Rating (CDR) Dementia Staging Instrument.

Supplementary Table S1 lists the diagnostic and selection criteria for each study considered. For a complete list of subjects, diagnoses, and data sets used in this study see Supplementary Table S2.

### MR imaging

Data used in the preparation of this article were obtained from the Alzheimer’s Disease Neuroimaging Initiative (ADNI) database (adni.loni.usc.edu). The ADNI was launched in 2003 as a public–private partnership, led by Principal Investigator Michael W. Weiner, MD. The primary goal of ADNI has been to test whether serial magnetic resonance imaging (MRI), positron emission tomography (PET), other biological markers, and clinical and neuropsychological assessment can be combined to measure the progression of mild cognitive impairment (MCI) and early Alzheimer’s disease (AD).

ADNI and FTLDNI data were collected from the Imaging Data Archive (IDA) web-portal of the Laboratory of NeuroImaging (LONI) (http://adni.loni.usc.edu).

NACC and PDBP data were downloaded from their respective web portals: https://naccdata.org/ and https://pdbp.ninds.nih.gov/.

The Newcastle data were provided directly by the Translational and Clinical Research Institute, Newcastle University.

Table 1 reports the imaging characteristics for each sequence and data set. Combining data from multiple hospitals is useful to build ML models that are invariant to systematic inter-scanner effects and to overcome differences in field strengths and acquisition protocols^36^.

**Table 1:** Image characteristics for each data set.

	Scanner manufacturer	T13D		FLAIR		DTI		
		Field strength (T)	Voxel size (mm)	Field strength (T)	Voxel size (mm)	# Directions	Field strength (T)	Voxel size (mm)
ADNI*	GE Philips Siemens	1.5 3.0	1.2 × 1.0 × 1.0 1.0 × 1.0 × 1.0	3.0	0.86 × 0.86 × 5.0 1.2 × 1.0 × 1.0 1.0 × 1.0 × 1.0	From 19 to 114	3.0	1.37 × 1.37 × 2.7 1.37 × 1.37 × 2.9 2.0 × 2.0 × 2.0 2.17 × 2.17 × 2.0 0.91 × 0.91 × 2.0 2.67 × 2.67 × 2.0 1.22 × 1.22 × 4.0
FTLDNI	GE Siemens	3.0	1.0 × 1.0 × 1.0 1.2 × 1.02 × 1.02 1 × 1.14 × 1.14	3.0	0,86 × 0,86 × 3.0 1 × 0.98 × 0.98 1 × 0.49 × 0.49	From 30 to 64	3.0	2.73 × 2.73 × 2.7 1.37 × 1.37 × 2.7 2.2 × 2.2 × 2.2 1.87 × 1.87 × 6.5
NACC	GE Siemens	1.5 3.0	1.0 × 1.0 × 1.0 1 × 0.98 × 0.98 1.2 × 1.02 × 1.02 1.2 × 1.05 × 1.05 0.78 × 1.6 × 0.78 0.98 × 1.5 × 0.98 0.98 × 1 × 0.98 1.0 × 1.0 × 1.2	1.5 3.0	1.0 × 1.0 × 1.0 2.0 × 1.0 × 1.0 0.94 × 0.94 × 3.3 0.97 × 0.97 × 2.0 0.98 × 0.98 × 1.0 0.98 × 0.98 × 3.0 0.86 × 0.86 × 3.0 0.86 × 0.86 × 3.6 0.69 × 0.69 × 4.0 0.43 × 0.43 × 3.0 0.43 × 0.43 × 7.0 1.0 × 0.5 × 0.5	From 15 to 80	1.5 3.0	0.94 × 0.94 × 2.5 0.94 × 0.94 × 2.9 0.94 × 0.94 × 6.0 1.0 × 1.0 × 2.0 1.37 × 1.37 × 6 1.41 × 1.41 × 5 1.87 × 1.87 × 2.5 1.87 × 1.87 × 5.0 2.0 × 2.0 × 2.0 2.5 × 2.5 × 2.5
PDBP	Siemens	3.0	0.8 × 0.8 × 0.8 1.0 × 1.0 × 1.0	3.0	1.2 × 1.0 × 1.0	From 48 or 114	3.0	2.0 × 2.0 × 2.0 2.2 × 2.2 × 2.0
Newcastle	Philips	1.5 3.0	1.0 × 1.0 × 1.0 1.2 × 0.94 × 0.94 0.94 × 0.94 × 1.5	1.5 3.0	0.94 × 0.94 × 3.0 0.94 × 0.94 × 6.0 1.02 × 1.02 × 2.5	64	1.5 3.0	2.1 × 2.1 × 2.1 2.0 × 2.0 × 2.5 1.87 × 1.87 × 3.0

Information about scanner manufacturer, sequence type, field strength, dimensionality and directions are reported for each data set.

*GE* general electric, *FLAIR* fluid attenuated inversion recovery, *DTI* diffusion tensor imaging, * ADNI1, ADNI2 and ADNI3 data were included.

### Pipelines for image processing

N4 correction, from Advanced Normalization Tools (ANTs)^37^, was performed for all images to correct smooth intensity variations in MRI. The pipelines used for image processing in this study were FS version 6.0, LPA, and TRACULA.

FS is a pipeline for segmenting the cortical and subcortical brain structures using volumetric T13D images, where each voxel is labeled based on a probabilistic atlas^13,14^. The T13D MRIs were processed using the cross-sectional stream over the recon-all script using the Desikan-Killiany atlas and, when available in high quality, FLAIRs were used to improve the segmentation of the pial surfaces^38^. Volumes of subcortical regions in native space were normalized to FS estimated total intracranial volume (eTIV). Normalization was performed by dividing the volume of the region by the eTIV of the subject and multiplying the ratio by a reference value of 1409 ml^39^ to remove the effect of head size^40^. Cortical thickness values were not normalized^19^.

LPA is an algorithm for the quantification of the WM lesions that is part of the Lesion Segmentation Toolbox (LST)^41^. First, FLAIR images were linearly registered to T13D and each voxel was classified as cerebrospinal fluid (CSF), gray matter, or WM using the Statistical Parametric Mapping Tool v12.0 (SPM-12) tissue probability maps. Intensity distributions were calculated for each of them and weighted based on the spatial probability of belonging to WM. Finally, the map was converted to a binary lesion mask and its volume in native space (normalized to eTIV) was calculated.

TRACULA is a tool for automatic reconstruction of a set of 18 major WM pathways^16^. It uses prior information about the anatomy and relative positions of the WM tracts in relation to surrounding anatomical structures, obtained from a set of cognitively normal training subjects in which the tracts were manually labeled to produce tractography streamlines^42^. After mitigating image distortions due to eddy currents and B0 field inhomogeneities, TRACULA fits the shape of the tracts to both the subject's diffusion data and the anatomical neighborhood priors derived from the subject's T1 data. Fractional anisotropy (FA) and mean diffusivity (MD) were extracted from the diffusion data in MNI template space for each of the 18 reconstructed pathways. Then, the mean FA and mean MD of 48 ROIs were obtained from the WM John Hopkins University (JHU-ICBM-labels-1 mm) atlas^43^ and applied to the TRACULA maps.

**Figure 1:**
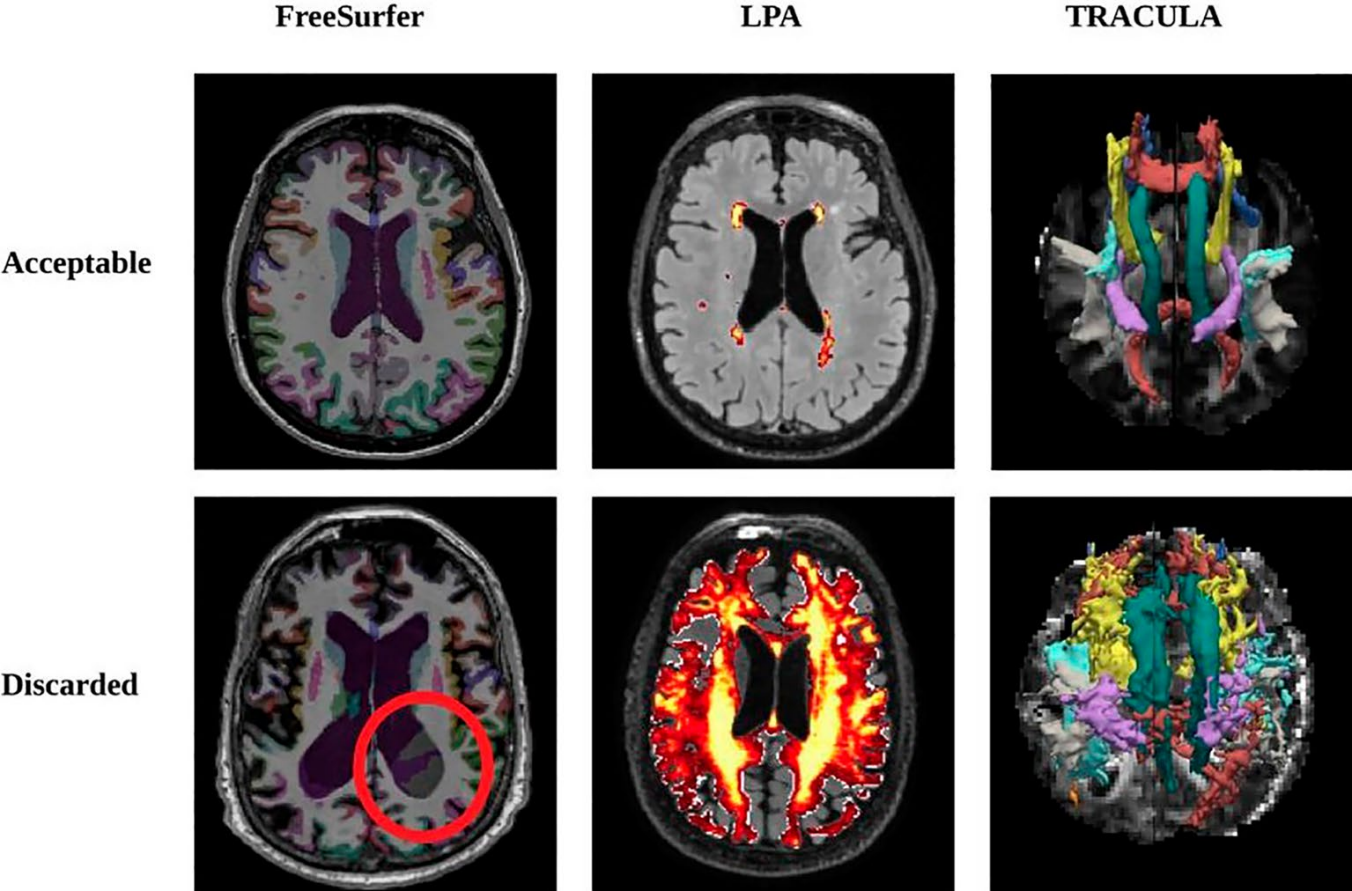
Acceptable and non-acceptable outputs of each image analysis pipeline. All images and outputs have been inspected slice by slice. Images of low quality, presenting artifacts or resulting in wrong segmentation or unrealistic reconstruction were discarded.

Quality control of the processed outputs was performed by experienced neuroscientists (SD, AR) who inspected the images and the results of each pipeline slice by slice, and discarded those with poor quality or incorrect segmentation (Fig. 1). The influence of WM-hyperintensity load on FA and MD values in MUQUBIA selected tracts was assessed with two multivariate linear regression models^44^ (Supplementary Table S3). To investigate possible bias due to different image acquisition protocols in the datasets, we compared the distributions of MRI features of subjects with the same diagnosis from different datasets (inter-cohort variability), and the distributions of MRI features of subjects with different diagnoses from the same dataset (intra-cohort variability) (Supplementary Fig. S8).

### MUQUBIA classification steps

**Figure 2:**
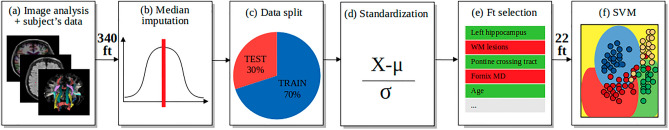
Steps to create and test MUQUBIA. (**a**) Images of 506 subjects were processed to obtain the full set of features. (**b**) Missing values were replaced with median values. (**c**) The data were split into training set (70% of the subjects) and test set (30%) to avoid any bias in the selection of features and in the classification performance. (**d**) Values were standardized. (**e**) The full set of features was pruned to avoid overfitting using a bidirectional sequential feature selection approach. (**f**) The non-linear SVM model was built and fine-tuned on the training and validation sets, while being tested on the test set left aside. Acronyms: ft, features; MD, Mean Diffusivity; SVM, Support Vector Machine; WM, White Matter.

Figure 2 shows the workflow for the creation of the Support Vector Machine (SVM) model.

The imaging biomarkers, CDR scores, and sociodemographic information served as input to the SVM algorithm, which was run in Python 3.7.11. The framework we used was based on the *scikit-learn* library version 0.22.2^45^.

The data set was randomly shuffled, with 70% of subjects forming the training set and 30% forming the test set. All 5 data sets were included in both the training and test data sets. None of the features resulted in more than 50% missing data. For the missing values, we employed the median as a method of imputation^46^. The statistical comparison between the original biomarker values of the training and test sets is presented in Supplementary Table S6 to demonstrate homogeneity between the two groups. All values were standardized by removing the mean and scaling to the variance of the feature distributions of the subjects from the whole training sample (z-scores).

To test the adequacy of the training sample size we modeled the relationship between training sample size and accuracy using the post-hoc “learning curve fitting” method^47^. The results are shown in Supplementary Fig. S1.

Machine learning models tend to overfit and become less generalizable when dealing with high-dimensional features, a well-known phenomenon called the “curse of dimensionality”^48^. A large set of features generally implies the presence of irrelevant, redundant, or correlated variables. To overcome this, our algorithm performed feature selection, considering only those features that maximized the accuracy of the classification task in the training set evaluated with a five-fold cross validation (CV) approach. This procedure allowed us to determine which variables were most informative for the diagnostic categories selected in this study. To determine the best set, a forward and backward sequential feature selection approach was followed, with each feature added to the model individually^49^. If accuracy increased, the feature was considered important; otherwise, it was discarded. After the selection process was completed, the surviving features were further reduced to obtain a Variance Inflation Factor (VIF) below the threshold value of 5 for each of them (see Table 4), indicating that there was no collinearity^50^.

To increase computational efficiency, the one-versus-rest (OVR) method was used to transform the multi-class problem into multiple binary classifications. The classification results were obtained using a non-linear SVM^51^. We optimized the search for the best hyperparameters using a five-fold CV splitting strategy over a grid search to find the best combination of SVM kernel, C and *γ* values*.* We also used L2 regularization.

Finally, SVM performance was evaluated using the following metrics: accuracy, precision, recall, F1 score, Area Under the Curve (AUC), Receiver Operating Characteristic curve (ROC). The global metrics, except for the accuracy, are macro-averaged, that is the arithmetic mean of the individual class performance.

In the context of ML, interpretability is necessary to explain the outcome of a model. In this study, Shapley values were calculated using the library SHAP, version 0.40.0^25^, to better understand the contribution of each feature expression.

The clinical challenge for the MUQUBIA algorithm was to distinguish between the different types of dementia. Because CDR is a clinical score collected by clinicians during the assessment process to differentiate the healthy from the dementia state, we evaluated the performance of our model even without including this scale in the feature set (Supplementary Fig. 2) to avoid circularity and minimize potential bias in favor of CN classification.

### Statistics

Differences in the variances of the feature distribution of each diagnostic class between the original data set and the data set with imputed medians were assessed using the Brown-Forsythe test. Differences in sociodemographic, clinical, neuropsychological and morphological feature distributions among diagnostic groups, and inter- intra-cohort differences were assessed using the Kruskal–Wallis test for continuous variables and the Chi-squared test for dichotomous variables. Post-hoc analyses were performed to test differences between the four diagnostic groups by pairwise comparisons of the Wilcoxon rank sum test for continuous variables and a pairwise comparison between pairs of proportions for dichotomous variables. The p-values of the post-hoc analyses were adjusted with the Benjamini–Hochberg correction. To compare the neuropathological multilabel evidence with the MUQUBIA results, the metric LRAP (Label Ranking Average Precision) was calculated. Similarity between test and train ROC curves was assessed using the DeLong’s test. All statistical analyses were performed using R version 3.6.3, and the significance level was set at 0.05 for all tests.

### Pipeline availability

The single subject classification tool based on the MUQUBIA models was also made publicly available through the neuGRID platform (https://neugrid2.eu)^21,52,53^, an on-line high-performance computing (HPC) infrastructure that provides source code, tools, and data for image processing and ML analysis (see Supplementary Fig. S5).

## Results

### Subjects

The final data set included 506 subjects: 110 AD, 135 FTD, 153 DLB and 108 CN. Demographic, clinical, neuropsychological, and ApoE information are shown in Table 2. Only neuropsychological tests that followed the same protocol in all 5 data sets were considered.

**Table 2:** Group characteristics.

	AD	FTD	DLB	CN	*P* values
Age (years)	73.6 ± 7.7 (n = 110)	63.9 ± 6.9 (n = 135)	73.9 ± 8.6 (n = 150)	74.3 ± 10.3 (n = 108)	< 0.05^°£ç^
Gender (% of females)	40.9% (n = 110)	40% (n = 135)	18.4% (n = 152)	51.8% (n = 108)	< 0.05^^^*^ç^
Handedness (% right)	90% (n = 109)	N.A	91% (n = 71)	90% (n = 98)	0.92
Disease duration (years)	4.6 ± 2.7 (n = 109)	N.A	4.8 ± 3.6 (n = 65)	N.A	0.75
Education (years)	15.3 ± 2.7 (n = 104)	16.25 ± 3.2 (n = 112)	13.9 ± 4 (n = 152)	16.4 ± 2.8 (n = 106)	< 0.05^§^^*^ç^
CDR*®*	0.9 ± 0.4 (n = 61)	0.8 ± 0.5 (n = 133)	1.1 ± 0.6 (n = 42)	0.04 ± 0.1 (n = 97)	< 0.05^§^*^°£ç^
NPI-Q	4.2 ± 3.6 (n = 56)	10.1 ± 6.3 (n = 117)	5.8 ± 5.6 (n = 83)	0.7 ± 1.5 (n = 56)	< 0.05^§^*^°£ç^
GDS	1.9 ± 2.2 (n = 60)	3.5 ± 3.1 (n = 105)	3.8 ± 3.1 (n = 67)	1.1 ± 1.5 (n = 100)	< 0.05^§^°^*^£^
MMSE	21.9 ± 3.9 (n = 101)	24.9 ± 4.4 (n = 128)	21.5 ± 5.1 (n = 85)	28.9 ± 1.3 (n = 107)	< 0.05^§°^*^£ç^
Category Fluency—Animals	11 ± 5.2 (n = 74)	9.8 ± 6.4 (n = 124)	9.8 ± 3.9 (n = 66)	20.2 ± 5.3 (n = 89)	< 0.05^§^*^£^
Digit Forward	6.2 ± 1.3 (n = 25)	5.7 ± 1.4 (n = 123)	5.5 ± 1.0 (n = 26)	6.7 ± 1.1 (n = 26)	< 0.05*^£^
Digit Backward	3.9 ± 1.2 (n = 25)	3.9 ± 1.4 (n = 126)	3.3 ± 0.9 (n = 26)	5 ± 1.3 (n = 27)	< 0.05^§^*^£^
ApoE4 (% carriers)	65.9% (n = 91)	N.A	49.1% (n = 55)	33.0% (n = 97)	< 0.05^§^

Values are expressed as mean ± standard deviation or percentage (%). *P* values were determined using the Kruskal–Wallis test for continuous variables and the Chi-squared test for dichotomous variables (α = 0.05). Values in brackets indicate the number of subjects for whom the characteristic is available. (§, post-hoc significant difference between AD and CN; ^, post-hoc significant difference between AD and DLB; °, post-hoc significant difference between AD and FTD; *, post-hoc significant difference between CN and DLB; £, post-hoc significant difference between CN and FTD; ç, post-hoc significant difference between DLB and FTD).

*n* sample size, *CDR®* clinical dementia rating dementia staging instrument, *NPI-Q* neuropsychiatric inventory questionnaire, *GDS* Geriatric Depression Scale, *MMSE* mini-mental state examination, *AD* Alzheimer’s dementia, *FTD* frontotemporal dementia, *DLB* dementia with Lewy bodies, *CN* cognitively normal controls, *ApoE* apolipoprotein E, *N.A.* not available.

### Feature set and sanity check

Image processing yielded a total of 336 features: 202 from FS (including 132 volumes and 70 cortical thickness values); 2 from LPA (WM lesion volume and WM lesion number); 36 from TRACULA (18 FA, 18 MD values for WM pathways); 96 features from the application of the JHU atlas ROIs to the FA and MD maps. The full list of features is reported in Supplementary Table S4.

Table 3 reports the number of outputs deemed acceptable after visual inspection for each pipeline and diagnostic group, as well as the consistency of the success rate for each pipeline in the 4 diagnostic groups.

**Table 3:** Number of correctly processed images and success rate of image processing after visual inspection.

	FS	LPA	TRACULA
AD	102 (93%)	81 (74%)	86 (78%)
FTD	132 (98%)	120 (89%)	109 (81%)
DLB	149 (97%)	116 (94%)	81 (77%)
CN	100 (93%)	85 (79%)	95 (88%)
*P* value	0.07	< 0.05	0.17

Numeric values denote the number of outputs that were deemed acceptable after visual inspection for each pipeline in each diagnostic group. Percentages indicate the success rate of each pipeline after visual quality inspection by two raters. *P* values were obtained with the Chi-squared test (α = 0.05).

*FS* FreeSurfer version 6.0, *LPA* Lesion Prediction Algorithm, *AD* Alzheimer’s dementia, *FTD* frontotemporal dementia, *DLB* dementia with Lewy bodies, *CN* cognitively normal controls.

### MUQUBIA algorithm

The training sample of MUQUBIA included 354 subjects, while 152 subjects formed the test group. The best hyper-parameters among those tested with the GridSearchCV function (i.e. kernel: linear, polynomial, sigmoid, radial basis function (RBF); C: 1, 10, 100, 1000, 10,000; γ: 0.1, 0.01, 0.001, 0.0001, 0.00001), were RBF kernel, C equal to 1000, and *γ* equal to 0.0001. For the entire analysis, consisting of image processing and classification of the subjects, the algorithm requires 10 h on a machine running Ubuntu Server 18.04 LTS version on a Sun Grid Engine scheduler equipped with 1300 GB RAM and 214 cores. Most of the requested time is spent for image analysis.

**Figure 3:**
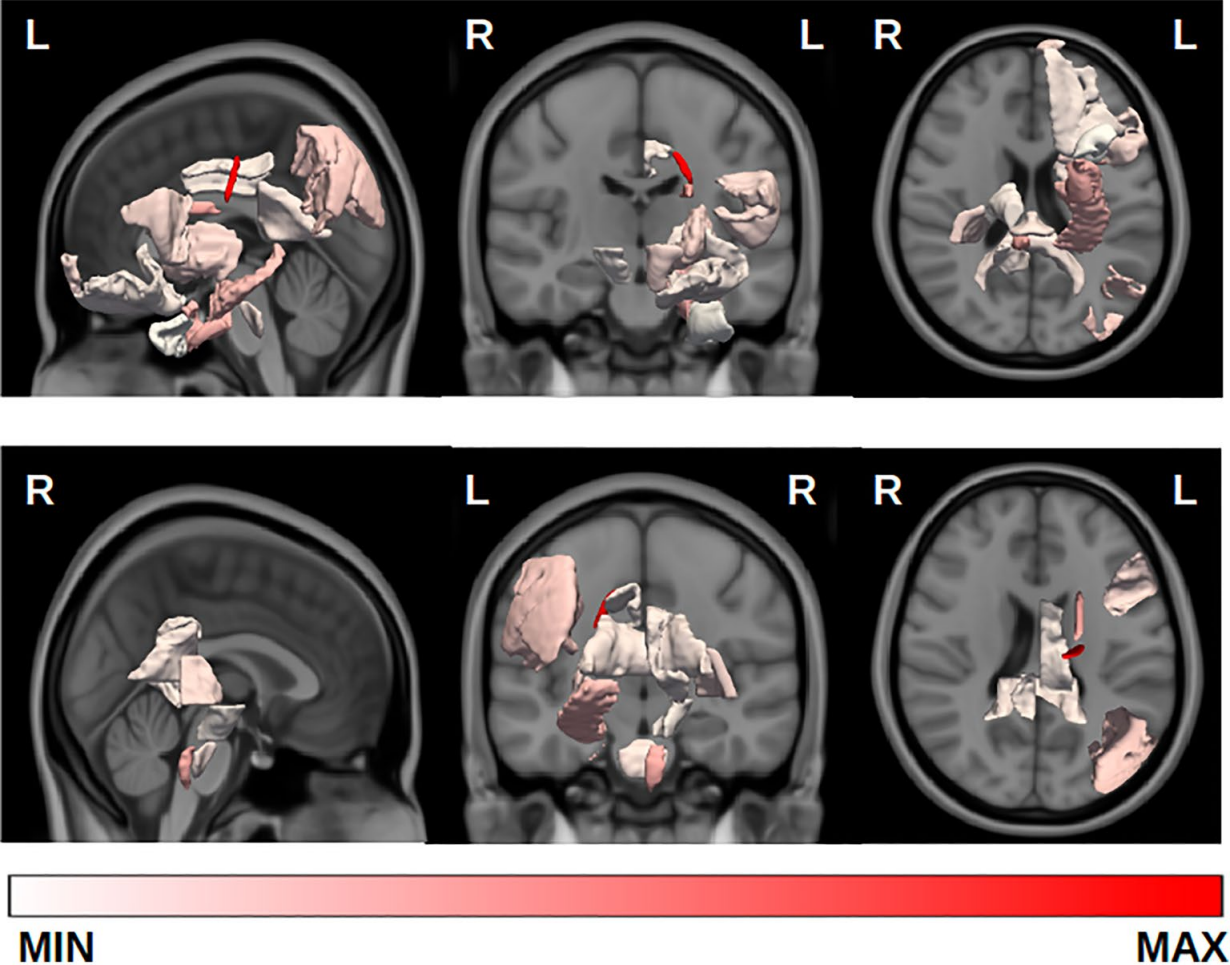
Representation of brain regions corresponding to imaging features selected by MUQUBIA to distinguish the different diagnostic classes (AD, DLB, FTD, CN). The color of each brain region reflects the ability of the corresponding feature to discriminate among the different classes (averaged mean Shapley value). Acronyms: L, left; R, right.

The algorithm selected 24 features, but two of them were discarded because of a VIF above 5, namely: fractional anisotropy of the left retrolenticular part of the internal capsule and left postcentral thickness. Figure 3 shows the imaging features selected by the bidirectional selection process implemented in MUQUBIA. The 22 features composing the best set are listed in Table 4. The features were ranked from highest to lowest importance in distinguishing the four diagnostic classes. The set of best features was composed by CDR, 19 MRI features, age and gender. The influence of age and gender on the MRI features was assessed and the results are reported in Supplementary Table S5. Across all diagnoses, CDR was the most important feature. The results of the Kruskal–Wallis test showed that the diagnostic groups differed significantly with respect to the selected variables. Post-hoc analyses revealed *p*-values below 0.05 in at least one comparison for all the features.

**Table 4:** Best set of features selected by MUQUBIA.

Name	Pipeline	Type	AD	FTD	DLB	CN	*P* values	VIF factor
CDR*®*	//	Clinical	0.94 ± 0.28	0.76 ± 0.55	1.01 ± 0.31	0.04 ± 0.14	< 0.05^§°^*^£ç^	1.6
LH corticospinal tract	TRACULA	FA	0.74 ± 0.12	0.73 ± 0.11	0.56 ± 0.08	0.78 ± 0.16	< 0.05^§^^*^£ç^	3.8
LH superior fronto-occipital fasciculus	JHU	FA	0.38 ± 0.20	0.37 ± 0.20	0.35 ± 0.13	0.60 ± 0.26	< 0.05^§^*^£^	3
RH medial lemniscus (mm^2^/s)	JHU	MD	5 × 10^–4^ ± 2 × 10^–4^	1 × 10^–3^ ± 4 × 10^–4^	6 × 10^–4^ ± 1 × 10^–4^	5 × 10^–4^ ± 3 × 10^–4^	< 0.05^^°^*^£ç^	2.2
LH entorhinal (mm)	FS	Thickness	2.61 ± 0.55	2.53 ± 0.76	2.92 ± 0.52	3.16 ± 0.38	< 0.05^§^^*^£ç^	3.3
LH hippocampus (mm^3^)	FS	Volume	2887 ± 432	2966 ± 591	2973 ± 430	3544 ± 445	< 0.05^§^*^£^	2.5
Age (years)	//	Demographic	74.07 ± 8.14	64.73 ± 7.11	74.26 ± 8.46	73.98 ± 10.02	< 0.05^°£ç^	1.5
LH inferior parietal (mm)	FS	Thickness	2.24 ± 0.33	2.49 ± 0.20	2.42 ± 0.30	2.40 ± 0.30	< 0.05^§^°^	3.1
LH cortex (mm^3^)	FS	Volume	193,461 ± 23,426	195,671 ± 22,459	190,081 ± 21,871	213,303 ± 22,509	< 0.05^§^*^£^	4.6
LH putamen (mm^3^)	FS	Volume	3795 ± 679	3556 ± 711	3696 ± 502	4061 ± 595	< 0.05^§^*^£^	1.8
LH frontal pole (mm^3^)	FS	Volume	826 ± 193	975 ± 196	917 ± 234	943 ± 187	< 0.05^§^°ç^	1.4
RH retrolenticular part of internal capsule (mm^2^/s)	JHU	MD	9 × 10^–4^ ± 3 × 10^–4^	9 × 10^–4^ ± 3 × 10^–4^	8 × 10^–4^ ± 2 × 10^–4^	7 × 10^–4^ ± 3 × 10^–4^	< 0.05^§^^*^£ç^	1.8
LH pars opercularis (mm)	FS	Thickness	2.41 ± 0.34	2.47 ± 0.30	2.55 ± 0.32	2.58 ± 0.27	< 0.05^§^^	3.7
Pontine crossing tract (mm^2^/s)	JHU	MD	5 × 10^–4^ ± 1 × 10^–4^	5 × 10^–4^ ± 1 × 10^–4^	6 × 10^–4^ ± 1 × 10^–4^	4 × 10^–4^ ± 2 × 10^–4^	< 0.05^§^^*^£ç^	3.2
Gender (% of females)	//	Demographic	36%	47%	17%	50%	< 0.05^^^*^ç^	1.4
Splenium of corpus callosum	JHU	FA	0.57 ± 0.15	0.62 ± 0.12	0.49 ± 0.10	0.69 ± 0.18	< 0.05^§^°^*^£ç^	3
LH pallidum (mm^3^)	FS	Volume	1743 ± 290	1675 ± 220	1697 ± 206	1785 ± 242	< 0.05*^£^	1.6
RH isthmus cingulate (mm)	FS	Thickness	2.24 ± 0.29	2.33 ± 0.22	2.28 ± 0.26	2.38 ± 0.27	< 0.05^§^*	1.6
LH lateral orbitofrontal (mm^3^)	FS	Volume	6873 ± 1323	6367 ± 1346	7471 ± 1210	7355 ± 891	< 0.05^§^°£ç^	2.4
LH posterior cingulate (mm)	FS	Thickness	2.38 ± 0.28	2.48 ± 0.20	2.37 ± 0.27	2.49 ± 0.24	< 0.05^§°^*^ç^	1.7
RH cerebral peduncle (mm^2^/s)	JHU	MD	6 × 10^–4^ ± 2 × 10^–4^	7 × 10^–4^ ± 2 × 10^–4^	8 × 10^–4^ ± 2 × 10^–4^	6 × 10^–4^ ± 3 × 10^–4^	< 0.05^^°^*^£^	3.2
LH temporal pole (mm)	FS	Thickness	3.16 ± 0.52	3.06 ± 0.72	3.31 ± 0.52	3.47 ± 0.43	< 0.05^§^£ç^	3

Values denote the mean ± standard deviation or percentage of variables that best classified subjects into the 4 diagnostic groups, ordered by Shapley values. *P* values were determined using the Kruskal–Wallis test or the Chi squared test (α = 0.05) (§, Post-hoc significant analysis difference between AD and CN; ^, Post-hoc significant analysis difference between AD and DLB; °, Post-hoc significant analysis difference between AD and FTD; *, Post-hoc significant analysis difference between CN and DLB; £, Post-hoc significant analysis difference between CN and FTD; ç, Post-hoc significant analysis difference between DLB and FTD).

*AD* Alzheimer’s dementia, *CDR®* clinical dementia rating dementia staging instrument, *CN* cognitively normal controls, *DLB* dementia with Lewy bodies, *FTD* frontotemporal dementia, *FS* FreeSurfer version 6.0, *LH* left hemisphere, *RH* right hemisphere, *FA* fractional anisotropy, *MD* mean diffusivity, *VIF* variance inflation factor.

The Brown-Forsythe test always yielded a p-value greater than 0.05 (Supplementary Table S7), indicating that the original variance of the data set was not altered by median imputation.

### SHAP analysis

**Figure 4:**
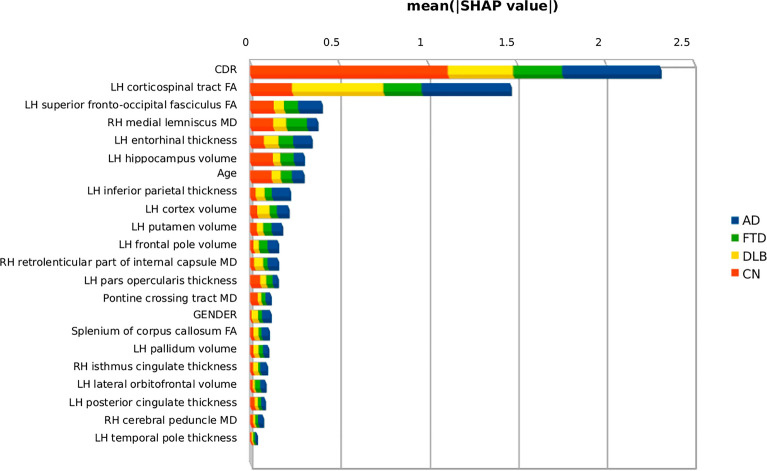
Contribution of each feature to the classification, represented by the mean Shapley magnitude values. The graph shows the importance of each variable for each diagnostic group. Acronyms: AD, Alzheimer’s Dementia; FTD, Frontotemporal Dementia; DLB, Dementia with Lewy Body; CN, Cognitive Normal; FA, Fractional Anisotropy; MD, Mean Diffusivity; LH, left hemisphere; RH, right hemisphere.

Figure 4 shows the average influence of the features on the prediction of each diagnosis, the values of CDR have the greatest influence especially for the classification of CN and AD, whereas the FA of the left corticospinal tract, among the others, influences the classification of DLB and AD groups the most.

**Figure 5:**
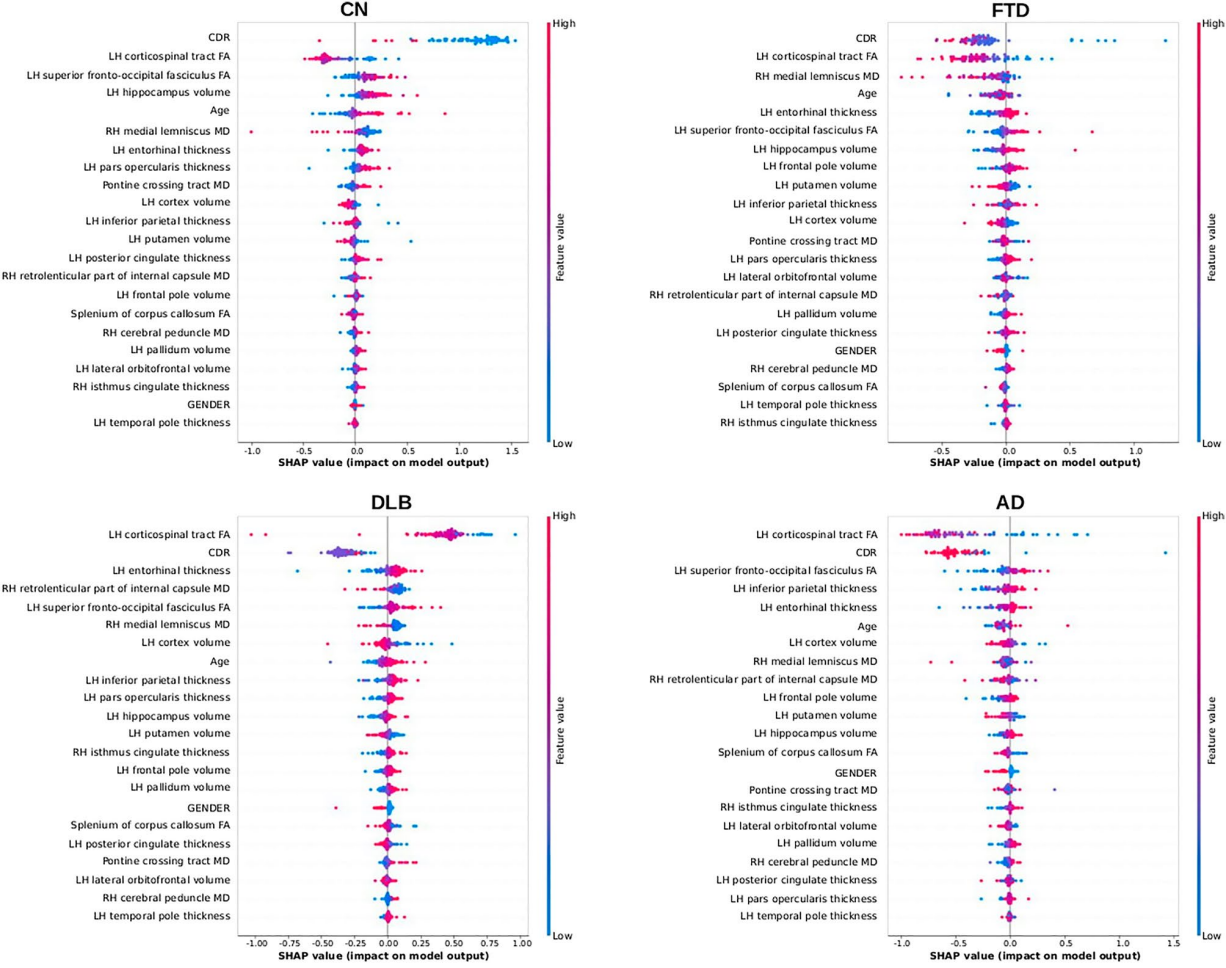
Global interpretability plots for each diagnostic class. Each dot corresponds to a subject in the training set. The position of the dot on the x-axis shows the effect of that feature on the prediction of the model for that subject. If multiple dots land at the same x position, they piled up to show density. The features are ordered by the sum of the Shapley values. Colors are used to display the standardized value of each feature (colder colors represent lower values, warmer colors represent higher values). Acronyms: AD, Alzheimer’s Dementia; FTD, Frontotemporal Dementia; DLB, Dementia with Lewy Body; CN, Cognitive Normal; LH, left hemisphere; RH, right hemisphere; FA, Fractional Anisotropy; MD, Mean Diffusivity.

The global interpretability plot (Fig. 5), shows whether a feature shifts the MUQUBIA prediction toward other diagnostic classes and the relative contribution of each feature. The plot consists of all points standardized. Focusing on the CN class, low values of CDR have a very high impact on the determination of this diagnosis. High values of temporal ROIs (left hippocampal volume and left entorhinal thickness) also have a high influence, as does a low MD value of right medial lemniscus. Other MRI measures do not provide simple or practical information on how they influence MUQUBIA outcome. Atrophy of the left frontal pole, associated with the increase of MD in the right medial lemniscus and the decrease of FA in the fronto-occipital fasciculus, influences the prediction of FTD class in addition to the degeneration of the corticospinal tract. For DLB class, the corticospinal tract represented an imaging biomarker of great importance, especially with a reduced value of FA, although this tract is not a classic biomarker for DLB. Other imaging biomarkers, such as preservation of MD in the retrolenticular part of the internal capsule and preservation of left cortical thickness (entorhinal and inferior parietal), have an impact on the classification of DLB patients. For AD, lack or moderate impairment of FA for the corticospinal tract and high scores for CDR have a major impact on classification, followed by damage and shrinkage of some ROIs of the left hemisphere, such as: left superior fronto-occipital fasciculus, inferior-parietal thickness, entorhinal thickness. In general, age represents one of the most important factors for classification in all dementias.

**Figure 6:**
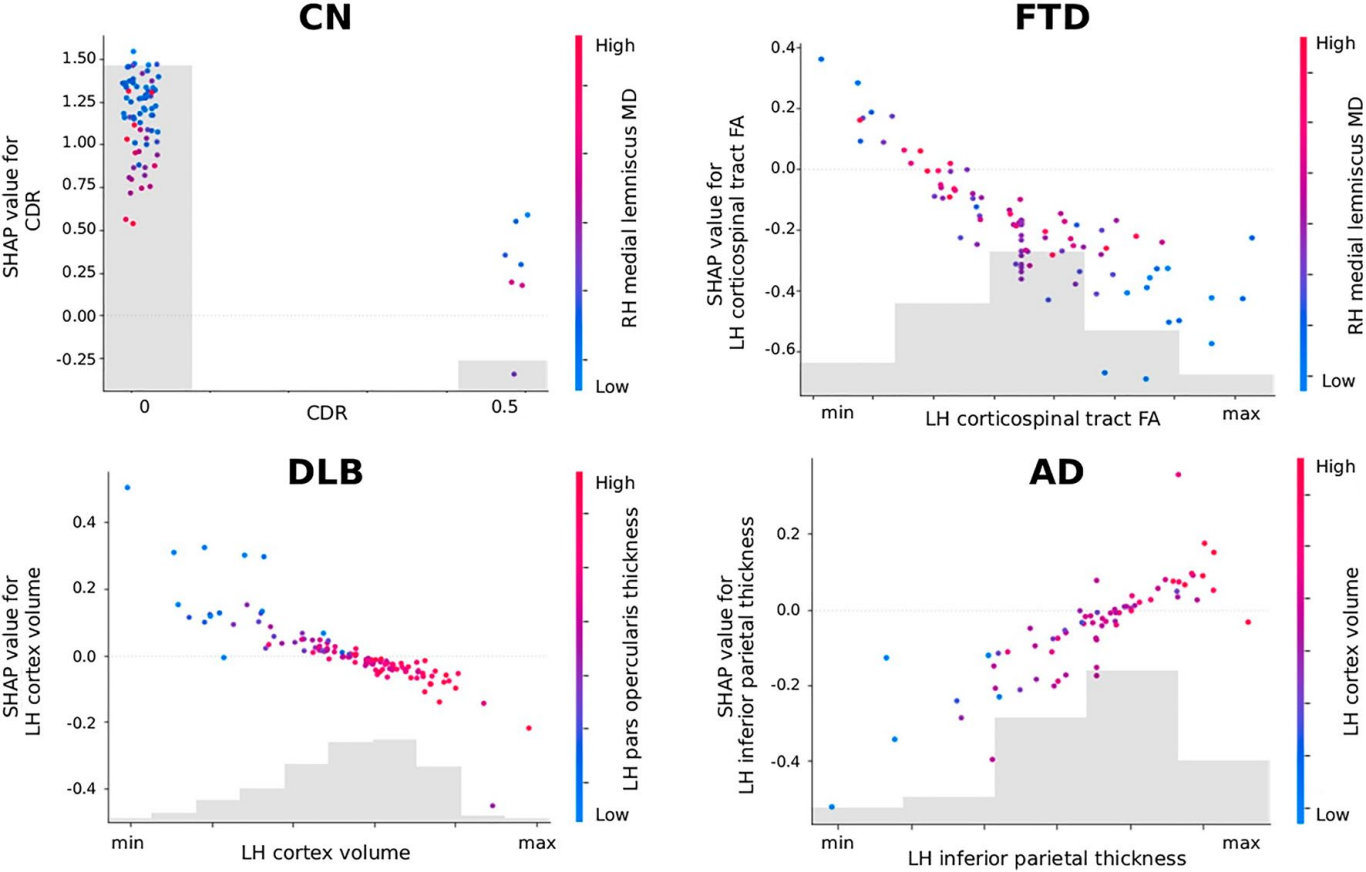
SHAP partial dependence plots for each diagnostic class (AD, DLB, FTD, CN). Each subplot shows the marginal effect that two features have on the predicted diagnosis. Once the first feature is chosen, the second is selected based on the feature with which the first feature interacts most strongly. The color of a dot indicates the value for the second feature. The color of each plot changes progressively from blue to red (or vice-versa) as you move along the axes. Colder colors represent lower values, warmer colors represent higher values of the second feature. Acronyms: AD, Alzheimer’s Dementia; FTD, Frontotemporal Disease; DLB, Dementia with Lewy Body; CN, Cognitive Normal; LH, left hemisphere; RH, right hemisphere; FA, Fractional Anisotropy; MD, Mean Diffusivity.

Additional information can be derived from the partial dependence plot of the main features (Fig. 6). This plot shows the marginal effect that two features have on the predicted outcome of MUQUBIA. Once the first feature was selected, the second was automatically chosen, picking out the feature with the strongest interaction with first one. Most of the plots show complex correlations between the two features and the Shapley values (Supplementary Fig. S7), which are discussed in more detail in the “Discussion” section.

Finally, to increase the interpretability and to understand potential problems of MUQUBIA we analyzed some correctly and incorrectly predicted subjects in Supplementary Fig. S3 and in Supplementary Fig. S4.

### MUQUBIA performance on training set

The classification resulted in the following global metrics: accuracy 91.53%, macro-precision 91.62%, macro-recall 90.82%, macro-F1 score 90.92%, AUC 98.44%.

### MUQUBIA performance on test set

**Figure 7:**
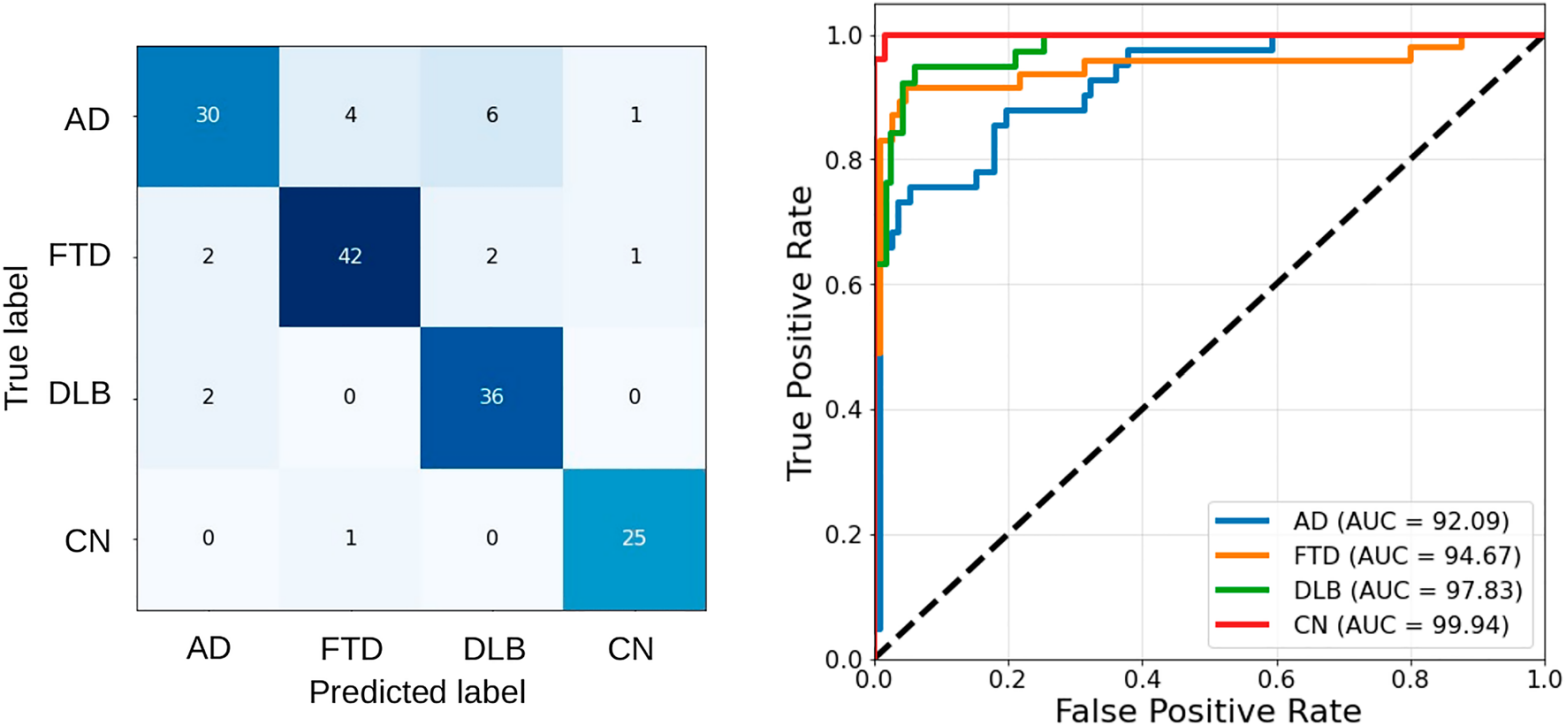
Confusion matrix and ROC curves of the test set. The AUC of each ROC curve for each diagnostic class against all others is reported in the legend. Acronyms: AD, Alzheimer’s Dementia; FTD, Frontotemporal Disease; DLB, Dementia with Lewy Body; CN, Cognitive Normal; AUC, Area Under the Curve.

The SVM classification task for the subjects in the test set (Fig. 7) resulted in the following global metrics: accuracy 87.50%, macro-precision 88.00%, macro-recall 88.36%, macro-F1 score 87.88%, AUC 97.79%. The DeLong test revealed no significant differences (*p* > 0.05) between the ROC curves of the training and test sets for each class. A summary of the performance metrics is provided in Table 5.

**Table 5:** MUQUBIA quantitative metrics for differential diagnosis in each diagnostic group of the test set.

	Accuracy (%)	Precision (PPV) (%)	Recall (%)	F1 score (%)	NPV (%)
AD	90	88	73	80	91
FTD	93	89	89	89	95
DLB	93	82	95	88	98
CN	98	93	96	94	99

Metrics used to determine the goodness of MUQUBIA in discriminating each diagnostic class.

*AD* Alzheimer’s dementia, *FTD* frontotemporal dementia, *DLB* dementia with Lewy bodies, *CN* cognitively normal controls, *PPV* positive predictive value, *NPV* negative predictive value.

Classification metrics obtained with MUQUBIA, trained with the same selected features but without CDR, are shown in Supplementary Fig. S2. Performance decreased slightly, especially in the case of CN. However, the classification task yielded the following global metrics: accuracy 84%, macro-precision 84%, macro-recall 84%, macro-F1 score 83%, AUC 96%.

### MUQUBIA performance on neuropathological assessed subsample of the test set

Table 6 reports the LRAP value used to compare the agreement between the MUQUBIA probability estimates with the National Institute on Aging and Alzheimer’s Association protocol^54^ for neuropathological assessment of 9 patients in our test group. The LRAP metric is classically used in multilabel ranking problems^55^. It determines the percentage of higher-ranked labels that resemble the true labels for each of the given samples. The score obtained is always greater than 0, and the best score is 1.

**Table 6:** MUQUBIA agreement with neuropathologic assessments.

ID	Neuropathology			MUQUBIA				LRAP
	ABC score	Lewy body pathology	FTLD with tau or other tauopathy	AD probability	DLB probability	FTD probability	CN probability	
011_S_0183	High ADNC	Neocortical (diffuse)	No	0.18	0.82	0.00	0.00	95%
027_S_4938	High ADNC	Amygdala (predominant)	Yes	0.37	0.05	0.58	0.01	
027_S_4962	High ADNC	Neocortical (diffuse)	Yes	0.46	0.15	0.08	0.32	
033_S_0724	High ADNC	Neocortical (diffuse)	No	0.03	0.97	0.00	0.00	
127_S_5058	Intermediate ADNC	Neocortical (diffuse)	No	0.47	0.26	0.26	0.01	
PDDJ916LE2	Intermediate ADNC	Limbic (transitional)	No	0.61	0.29	0.09	0.02	
PDEZ829YJX	High ADNC	Neocortical (diffuse)	No	0.20	0.77	0.01	0.03	
NACC047218	High ADNC	No	No	0.54	0.06	0.29	0.11	
NACC131130	High ADNC	Amygdala (predominant)	No	0.42	0.56	0.02	0.00	

The table reports the LRAP score derived considering the multilabel neuropathological ground truth (Montine’s criteria) of 9 subjects of our test set and the MUQUBIA classification probabilities. All the 9 subjects had cognitive impairment. 'Intermediate' or 'High' level of ADNC should be considered adequate explanation of AD dementia. 'Limbic', 'Neocortical' or 'Amygdala-predominant' level should be considered adequate explanation of Lewy Body Diseases and this does not preclude contribution of other diseases (e.g.: 'Amygdala-predominant LBD' typically occurs in the context of advanced AD neuropathologic change). Presence of frontotemporal lobar degeneration with tau or other tauopathy and subtypes were labeled as ‘Yes’.

*LRAP* label ranking average precision, *ADNC* NIA-AA Alzheimer’s disease neuropathologic change, *ABC* Aβ/amyloid plaques (A)—NFT stage (B)—and neuritic plaque score (C), *FTLD* frontotemporal lobar degeneration, *AD* Alzheimer’s dementia, *DLB* dementia with Lewy body, *FTD* frontotemporal dementia, *CN* cognitive normal.

### MUQUBIA report

An example of the MUQUBIA report generated with the on-line tool on the neuGRID platform is available as supplementary material (Supplementary Fig. S6).

## Discussion

In this work, we developed an automated ML algorithm based on multimodal MRI capable of discriminating the most common forms of dementia. The performance of this classifier was validated using quality metrics that resulted in high scores for accuracy, macro-precision, macro-recall, macro-F1 and AUC. The classifier was successful in discriminating between the 4 groups (AD, FTD, DLB and CN) characterized by different neuropsychological scores and ApoE expression (Table 2). The algorithm selected CDR, age, gender information, MRI-based diffusion metrics, volumetric and cortical thickness values as the best differentiating features.

SVM performance did not differ significantly between the test and training sets using 22 informative features; and performances on training set were higher than performance on the test set arguing against severe overfitting^56^.

In the test set group, MUQUBIA scored highest in discriminating CN from the others, with excellent discrimination performance for each diagnostic class. The lowest performance was in detecting the AD group. This could be due to the overlap with other types of dementia, especially DLB^57^. Neuropathological brains assessed by Montine’s criteria were also correctly classified by MUQUBIA with very good performance (LRAP = 95%).

The MRI features studied were appropriate to selectively distinguish AD, FTD, DLB and to differentiate them from cognitively normal aging. The neuroimaging features were extracted from FS and TRACULA pipelines, making mandatory only the T13D and DTI to run the MUQUBIA algorithm. Optionally, the FLAIR can be used to improve the pial segmentation and to reduce segmentation errors caused by WM hyperintensities. The WM hyperintensity information extracted from the LPA does not seem to affect MUQUBIA, as this aspect is likely already present in the DTIs as increased MD and decreased FA. It is known that WM hyperintensity may have an impact on the DTI metrics, although in the present study and in relation to the features selected by MUQUBIA, only the tract of the superior fronto-occipital fasciculus was weakly affected.

In addition to cortical/subcortical gray matter information, which has long been considered informative biomarkers, WM diffusion metrics have also been shown to be important for ML classification. These metrics appear to be useful in distinguishing AD from FTD^17^, and, albeit to a lesser extent, in distinguishing AD from DLB^35^.

The implemented data-driven MUQUBIA approach identified the best set of features, many of which were consistent with those described in the literature, while others were unexpected. For the benefit of the reader, the discussion of the results was organized according to the following 3 main macro-groups:

1. Clinical and socio-demographic features:

Among the most important features in our model there is the CDR, a well-known test for detecting and assessing the severity of dementia^58^; therefore, it is not surprising that it turned out to be the most informative feature. Interestingly, the SHAP partial dependence plot (Fig. 6) shows that the probability of being classified as cognitively normal by MUQUBIA is greater when the CDR score is zero and the MD value of the medial lemniscus tract is low, indicating no degeneration. Higher values of MD, may instead, progressively reduce the weight of the (non-pathological) CDR score in classifying a person as cognitively normal. This could be very promising information, especially for secondary prevention, which, by combining multimodal ad hoc biomarkers, would allow more accurate, sensitive, and earlier stratification of individuals at the pre-dementia stage than using CDR alone^59^. As expected, the MUQUBIA model without CDR performed worse in the classification of CN, but also in AD, DLB, and FTD confirming the importance of CDR also in the classification of dementia groups, as explained by the Shapley values (Fig. 4).

In addition, although neurological diseases are naturally assumed to affect only the elderly, this is not always the case. From the Shapley analysis, younger individuals belonging to the CN class are more likely to drop out (Fig. 5). The younger age of the FTD group must also be taken into account to explain possible brain imaging deviation and possible errors of our model.

Interestingly, according to the literature, DLB is associated with male preponderance^60^, and this was also observed in our DLB group. Finally, MUQUBIA seems to be strongly influenced by the degeneration of the left corticospinal tract, which is more pronounced in women than in men, when classifying AD subjects.

2. Cortical and sub-cortical features:

DLB is associated with less global atrophy than AD, whereas posterior cingulate atrophy was similar in AD and DLB. AD patients showed more atrophy of the medial temporal lobe structures compared to DLB^61^. Hippocampal atrophy was not limited to the AD and DLB groups, but has also been noted in FTD, although to a lesser extent than in AD^62^. Conversely, FTD patients showed greater atrophy of the temporal pole and orbitofrontal areas than AD patients, while AD patients showed greater atrophy of the posterior cingulate and inferior parietal regions^63^. In our study, no significant differences were found between DLB and CN with respect to the temporal pole, inferior parietal and orbitofrontal areas.

According to the literature, we found the putamen volume of AD is intermediate between CN and FTD, showing more atrophy in the latter^64^. DLB showed volumetric atrophy in the putamen^65^, with a moderate influence in the MUQUBIA model, or a slight influence in other basal ganglia such as the left pallidum^66^. Even in FTD, where there is limited and conflicting evidence in the literature regarding the volumetry of deep gray matter structures, our results tend to confirm the findings of Möller et al., with respect to the basal ganglia, and show that FTD patients are characterized by the most severe atrophy compared with other diagnostic groups as well as that atrophy of the pallidum contributes to the classification of FTD patients in MUQUBIA model. Further specific efforts will be needed to clarify this point in future studies.

Surprisingly, the volume of the left frontal pole was highest in FTD and differed significantly from all other patients examined in this study. This can be partly explained by the younger age of FTD compared with the other groups by approximately a decade. Consistent with the literature, patients with AD had smaller volumes of the frontal pole, isthmus of cingulate and left pars opercularis^67^ compared with CN subjects.

Cortical thickness was a sensitive and comprehensive marker to distinguish AD from other dementias. Cortical shrinkage of the left entorhinal cortex has been reported to be greater in AD than in DLB^68^, but similar in AD and FTD^69^. Left inferior parietal thickness, also greater in FTD, proved to be a robust marker to disentangle AD from FTD for MUQUBIA^70^.

Moreover, the SHAP partial dependence plot (Fig. 6) showed that MUQUBIA classifies patients as AD when a concomitant reduction in left inferior parietal thickness is associated with a reduction in total left cortical volume, which has been linked in previous studies to a decrease in semantic fluency^71^. Likewise, the SHAP partial dependence analysis (Fig. 6) revealed that MUQUBIA tends to classify patients in the DLB class when they exhibit lower total left cortical volume and a reduction in left parsopercularis thickness. This observation aligns with the existing literature, that links speech fluency impairment to these important regions in DLB^72,73^.

3. DTI feature

FA of the left corticospinal tract was lower in AD than in CN^74^. Degeneration of the corticospinal tract has also been described in FTD^75^. Instead, there is no clear evidence in the literature of damage of this tract in the DLB group^76^, although this tract had a major effect on MUQUBIA. Possible explanations may be found in the larger group size used in our study than in other efforts and the quality of the DTI pipeline and scans we used to quantify the DTI metrics.

FA of the splenium of the corpus callosum and the superior fronto-occipital fasciculus was lower in AD than in CN^72^, although the lowest FA values of these pathways occurred in DLB. DLB also showed lower values for FA than all other groups in many other pathways and ROIs^77^. According to the literature, DLB showed higher MD in brainstem areas^78^, such as in the pontine crossing tract, compared to CN. Other imaging biomarkers, such as the preservation of the retrolenticular part of the internal capsule, influenced MUQUBIA toward DLB classification. This is correct given that motor and sensory fibres run through this ROI^79^ and must be maintained integer to prevent dysphagia and swallowing dysfunction. FTD and AD were the most affected groups in the right retrolenticular part of the internal capsule^80^. The medial lemniscus MD proved to be the third most important feature for classifying FTD patients in MUQUBIA. As previously mentioned, FTD was characterized by the degeneration of the corticospinal tract^81^ similar to AD. The SHAP partial dependence plot (Fig. 6) for the FTD class also revealed that MUQUBIA finds a direct relationship between left corticospinal tract FA and right medial lemniscus MD values indicating a specific form of frontal neurodegeneration. Last but not least, the correlation between these two tracts could confirm interesting findings on the detection of subtypes of frontotemporal lobar degeneration^82^.

### Benefits from MUQUBIA

Recently, the number of studies using ML has steadily increased because ML enables a fully data-driven and automated approach. ML is indeed flexible in discovering patterns, complex relationships, and predicting unobserved outcomes in data, starting from a sufficient number of observations^83^, especially with increasing complexity, where classical statistical methods may be rather ineffective^84^.

Research studies often address the binary classification between two clinical conditions (i.e.: AD vs. CN; FTD vs. CN; FTD vs. AD, etc.…), but this does not reflect the reality of the clinician who needs to make a diagnosis considering multiple neurodegenerative diseases at the same time. Although the field of neurodegenerative diseases has been extensively researched^85^, to our knowledge, few studies have implemented an MRI-based ML algorithm for the classification of AD, FTD, DLB and CN^56,86,87^, and to date, no study has used DTIs and multimodal analyses simultaneously. MUQUBIA is the first ML algorithm for differential diagnosis to use DTI together with T13D and FLAIR on a very robust sample size. In fact, Klöppel et al. recruited a small group of FTD and DLB, whereas Koikkalainen et al. and Tong et al. included a broader range of dementias (such as vascular dementia and subjective memory complaints), but still with fewer subjects per group and with worse performance compared with MUQUBIA (i.e.: Klöppel et al.: accuracy of 65%; Koikkolainen et al.: accuracy of 70.6%; Tong et al.: accuracy of 75.2%). Moreover, Tong et al. used CSF biomarkers that required an invasive procedure such as lumbar puncture which is difficult to obtain in a large population. This could also affect the applicability in daily routine and clinical practice in hospitals compared to the data needed as input to MUQUBIA. Many advanced research frameworks recommend the analysis of amyloid, tau, or ^18^F-fluorodeoxyglucose positron emission tomography (PET) scans of the brain and CSF to better classify patients^88^. However, these expensive procedures may limit their actual utility and are not available in the normal clinical setting. MUQUBIA requires routinely available MRIs, a clinical test, and a few demographic information, so it can be considered widely applicable without incurring excessive costs and burdening patients unnecessarily.

The online MUQUBIA tool does not require manual or “a priori” preprocessing, and the end-user does not need to have prior knowledge of the algorithm, although a quality check of the ROI segmentation is always advisable.

In addition, experienced neuroradiologists are often not available in routine clinical practice outside of a specialized memory clinic, so an automated method capable of extracting and interpreting the information with high precision would be of great clinical value.

A strength of this study is that the DTIs followed heterogeneous acquisition protocols, e.g., gradient directions vary from a minimum of 19 (low) to a maximum of 114 (high). The FLAIR and T13D parameters differed, bringing this study closer also to a real-world clinical scenario.

### Limitations and future developments

We have considered various types of neurodegenerative diseases, which account for a large proportion of dementia cases, but this approach to differential diagnosis is far from complete. We did not attempt to define subtypes, such as posterior cortical atrophy in AD or the language or semantic variant in FTD or psychiatric and delirium onset in DLB. This study has limitations related to a partial influence of age and gender on certain MRI features, particularly in the FTD or in DLB. In fact, FTD group is the youngest and has an average age of onset of 56 years, while AD and DLB occurs later^9^. DLB group instead showed a preponderance of male. These confounders could help the classifier to identify more easily these groups and additional experiments should be performed to exclude this point. The fact that inter-cohort variability was lower than intra-cohort variability hints that the effect of etiology of dementia on MRI features is more important than potential bias induced by heterogeneous acquisition protocols, still the classifier might be further improved by trying to minimize the “center-effect” and reduce the few differences observed^89^.

Future efforts will aim to speed up processing times with new tools, such as FastSurferCNN, that exploit deep neural networks and graphical processor units to reduce image preprocessing in minutes.

Finally, due to difficulties finding datasets that contained multimodal and multiclass data, this study lacked a complete independent validation data set, but in the future, MUQUBIA should be validated with independent data sets given the upcoming Big-Data era.

## Conclusion

The fully automated classifier developed in this study can discriminate between AD, FTD, DLB and CN with good to excellent performance. Our ML classifier can help clinicians as a second opinion tool to better diagnose the different forms of dementia based on routine and cost-effective biomarkers such as age, gender, CDR and automatically extracted MRI features. It is important to point out that the interpretability and explainability of the methods of ML provide important clues, allow to go beyond the slogan “ML is a black-box”, and lead to the discovery of new informative data-driven candidate biomarkers.

### Supplementary Information

Supplementary Information.

## Data Availability

Publicly available data sets were analyzed in this study: ADNI and FTLDNI are accessible through the Laboratory of NeuroImaging (LONI) web portal (http://adni.loni.usc.edu). NACC and PDBP data are available through the following web portals: https://naccdata.org/ and https://pdbp.ninds.nih.gov/. MUQUBIA algorithm is publicly accessible through the neuGRID platform (https://www.neugrid2.eu).

## References

[1] GBD 2016 Neurology Collaborators (2019). Global, regional, and national burden of neurological disorders, 1990–2016: A systematic analysis for the Global Burden of Disease Study 2016. Lancet Neurol..

[2] Benussi A (2020). Classification accuracy of transcranial magnetic stimulation for the diagnosis of neurodegenerative dementias. Ann. Neurol..

[3] McKeith IG (2020). Research criteria for the diagnosis of prodromal dementia with Lewy bodies. Neurology.

[4] Van der Flier WM, Scheltens P (2018). Amsterdam dementia cohort: Performing research to optimize care. J. Alzheimers Dis..

[5] Onyike CU, Diehl-Schmid J (2013). The epidemiology of frontotemporal dementia. Int. Rev. Psychiatry.

[6] Young JJ, Lavakumar M, Tampi D, Balachandran S, Tampi RR (2018). Frontotemporal dementia: Latest evidence and clinical implications. Ther. Adv. Psychopharmacol..

[7] Armstrong RA, Lantos PL, Cairns NJ (2005). Overlap between neurodegenerative disorders. Neuropathology.

[8] Mayeux R (2004). Biomarkers: Potential uses and limitations. NeuroRx.

[9] Erkkinen MG, Kim MO, Geschwind MD (2018). Clinical neurology and epidemiology of the major neurodegenerative diseases. Cold Spring Harb. Perspect. Biol..

[10] Frisoni GB, Fox NC, Jack CR, Scheltens P, Thompson PM (2010). The clinical use of structural MRI in Alzheimer disease. Nat. Rev. Neurol..

[11] Risacher SL, Saykin AJ (2019). Neuroimaging in aging and neurologic diseases. Handb. Clin. Neurol..

[12] Amelio L, Amelio A, Tsihrintzis G, Sotiropoulos D, Jain L (2019). Classification methods in image analysis with a special focus on medical analytics. Machine Learning Paradigms. Intelligent Systems Reference Library.

[13] Fischl B (2002). Whole brain segmentation: Automated labeling of neuroanatomical structures in the human brain. Neuron.

[14] Fischl B (2004). Automatically parcellating the human cerebral cortex. Cereb. Cortex.

[15] Ribaldi F (2021). Accuracy and reproducibility of automated white matter hyperintensities segmentation with lesion segmentation tool: A European multi-site 3T study. Magn. Reson. Imaging..

[16] Yendiki A (2011). Automated probabilistic reconstruction of white-matter pathways in health and disease using an atlas of the underlying anatomy. Front. Neuroinform..

[17] Bron EE (2017). Multiparametric computer-aided differential diagnosis of Alzheimer's disease and frontotemporal dementia using structural and advanced MRI. Eur. Radiol..

[18] Dukart J, Mueller K, Barthel H, Villringer A, Sabri O, Schroeter ML (2013). Alzheimer's disease neuroimaging initiative. Meta-analysis based SVM classification enables accurate detection of Alzheimer's disease across different clinical centers using FDG-PET and MRI. Psychiatry Res..

[19] Westman E, Aguilar C, Muehlboeck JS, Simmons A (2013). Regional magnetic resonance imaging measures for multivariate analysis in Alzheimer's disease and mild cognitive impairment. Brain Topogr..

[20] Kim JP (2019). Machine learning based hierarchical classification of frontotemporal dementia and Alzheimer's disease. Neuroimage Clin..

[21] Archetti D (2021). Inter-cohort validation of SuStaIn model for Alzheimer's disease. Front. Big Data.

[22] Möller C (2016). Alzheimer disease and behavioral variant frontotemporal dementia: Automatic classification based on cortical atrophy for single-subject diagnosis. Radiology.

[23] Klöppel S (2008). Accuracy of dementia diagnosis: A direct comparison between radiologists and a computerized method. Brain.

[24] Erickson BJ, Korfiatis P, Akkus Z, Kline TL (2017). Machine learning for medical imaging. Radiographics.

[25] Lundberg, S. M. & Lee, S.-I. A unified approach to interpreting model predictions. In *Proceedings of the 31st International Conference on Neural Information Processing Systems* (*NIPS'17*). Curran Associates Inc., Red Hook, NY, USA, 4768–4777. Preprint at 1705.07874 (2017).

[26] Redolfi A (2020). Medical Informatics Platform (MIP): A pilot study across clinical Italian cohorts. Front. Neurol..

[27] Nigri A (2022). Quantitative MRI harmonization to maximize clinical impact: The RIN-neuroimaging network. Front. Neurol..

[28] Palesi F (2022). MRI data quality assessment for the RIN: Neuroimaging Network using the ACR phantoms. Phys. Med..

[29] Petersen RC (2010). Alzheimer's Disease Neuroimaging Initiative (ADNI): Clinical characterization. Neurology.

[30] Beekly DL (2007). The National Alzheimer's Coordinating Center (NACC) database: The uniform data set. Alzheimer Dis. Assoc. Disord..

[31] Ofori E, Du G, Babcock D, Huang X, Vaillancourt DE (2016). Parkinson's disease biomarkers program brain imaging repository. Neuroimage.

[32] Firbank MJ (2007). Diffusion tensor imaging in dementia with Lewy bodies and Alzheimer's disease. Psychiatry Res..

[33] Firbank MJ (2010). High resolution imaging of the medial temporal lobe in Alzheimer's disease and dementia with Lewy bodies. J. Alzheimers Dis..

[34] Firbank MJ (2016). Neural correlates of attention-executive dysfunction in Lewy body dementia and Alzheimer's disease. Hum. Brain Mapp..

[35] Donaghy PC (2020). Diffusion imaging in dementia with Lewy bodies: Associations with amyloid burden, atrophy, vascular factors and clinical features. Parkinsonism Relat. Disord..

[36] Archetti D (2019). Multi-study validation of data-driven disease progression models to characterize evolution of biomarkers in Alzheimer's disease. Neuroimage Clin..

[37] Tustison NJ (2010). N4ITK: Improved N3 bias correction. IEEE Trans. Med. Imaging.

[38] Lindroth H (2019). Examining the identification of age-related atrophy between T1 and T1 + T2-FLAIR cortical thickness measurements. Sci. Rep..

[39] Reite M (2010). Brain size and brain/intracranial volume ratio in major mental illness. BMC Psychiatry.

[40] De Francesco S (2021). Norms for automatic estimation of hippocampal atrophy and a step forward for applicability to the Italian population. Front. Neurosci..

[41] Schmidt P (2012). An automated tool for detection of FLAIR-hyperintense white-matter lesions in multiple sclerosis. Neuroimage.

[42] Watanabe A (2018). The detection of white matter alterations in obsessive-compulsive disorder revealed by TRActs Constrained by UnderLying Anatomy (TRACULA). Neuropsychiatr. Dis. Treat..

[43] Mori S (2008). Stereotaxic white matter atlas based on diffusion tensor imaging in an ICBM template. Neuroimage.

[44] Svärd D (2017). The effect of white matter hyperintensities on statistical analysis of diffusion tensor imaging in cognitively healthy elderly and prodromal Alzheimer's disease. PLoS ONE.

[45] Pedregosa F (2011). Scikit-learn: Machine learning in python. Journal of Machine Learning Research..

[46] Acuña E, Rodriguez C, Banks D, McMorris FR, Arabie P, Gaul W (2004). The treatment of missing values and its effect on classifier accuracy. Classification, Clustering, and Data Mining Applications. Studies in Classification, Data Analysis, and Knowledge Organisation.

[47] Balki I (2019). Sample-size determination methodologies for machine learning in medical imaging research: A systematic review. Can. Assoc. Radiol. J..

[48] Berisha V (2021). Digital medicine and the curse of dimensionality. NPJ Digit. Med..

[49] Jović, A., Brkić, K. & Bogunović, N. A review of feature selection methods with applications. In *38th International Convention on Information and Communication Technology, Electronics and Microelectronics (MIPRO),* 1200–1205 (IEEE, 2015). 10.1109/MIPRO.2015.7160458.

[50] Craney TA, Surles JG (2002). Model-dependent variance inflation factor cutoff values. Qual. Eng..

[51] Abraham A (2014). Machine learning for neuroimaging with scikit-learn. Front. Neuroinform..

[52] Redolfi A, Bosco P, Manset D, Frisoni GB, neuGRID consortium (2013). Brain investigation and brain conceptualization. Funct. Neurol..

[53] Redolfi A (2022). Italian, European, and international neuroinformatics efforts: An overview. Eur. J. Neurosci..

[54] Hyman BT (2012). National institute on aging-Alzheimer's association guidelines for the neuropathologic assessment of Alzheimer's disease. Alzheimers Dement..

[55] Liu Z (2021). Listening to mental health crisis needs at Scale: Using natural language processing to understand and evaluate a mental health crisis text messaging service. Front. Digit. Health.

[56] Klöppel S (2015). Applying automated MR-based diagnostic methods to the memory clinic: A prospective study. J. Alzheimers Dis..

[57] Koenig AM, Nobuhara CK, Williams VJ, Arnold SE (2018). Biomarkers in Alzheimer's, frontotemporal, Lewy body, and vascular dementias. Focus (Am. Psychiatr. Publ.).

[58] Huang HC, Tseng YM, Chen YC, Chen PY, Chiu HY (2021). Diagnostic accuracy of the Clinical Dementia Rating Scale for detecting mild cognitive impairment and dementia: A bivariate meta-analysis. Int. J. Geriatr. Psychiatry.

[59] Saxton J (2009). Functional and cognitive criteria produce different rates of mild cognitive impairment and conversion to dementia. J. Neurol. Neurosurg. Psychiatry.

[60] Kane JPM (2018). Clinical prevalence of Lewy body dementia. Alzheimers Res. Ther..

[61] Mak E, Su L, Williams GB, O'Brien JT (2014). Neuroimaging characteristics of dementia with Lewy bodies. Alzheimers Res. Ther..

[62] Muñoz-Ruiz MA (2012). Structural MRI in frontotemporal dementia: Comparisons between hippocampal volumetry, tensor-based morphometry and voxel-based morphometry. PLoS ONE.

[63] Möller C (2015). More atrophy of deep gray matter structures in frontotemporal dementia compared to Alzheimer's disease. J. Alzheimers Dis..

[64] Looi JC (2012). Differential putaminal morphology in Huntington's disease, frontotemporal dementia and Alzheimer's disease. Aust. N. Z. J. Psychiatry.

[65] Cousins DA (2003). Atrophy of the putamen in dementia with Lewy bodies but not Alzheimer's disease: An MRI study. Neurology.

[66] Watson R, Colloby SJ, Blamire AM, O'Brien JT (2016). Subcortical volume changes in dementia with Lewy bodies and Alzheimer's disease. A comparison with healthy aging. Int. Psychogeriatr..

[67] Vasconcelos LG (2014). The thickness of posterior cortical areas is related to executive dysfunction in Alzheimer's disease. Clinics (Sao Paulo).

[68] Sun X (2014). Destruction of white matter integrity in patients with mild cognitive impairment and Alzheimer disease. J. Investig. Med..

[69] Frisoni GB (1999). Hippocampal and entorhinal cortex atrophy in frontotemporal dementia and Alzheimer's disease. Neurology.

[70] Du AT (2007). Different regional patterns of cortical thinning in Alzheimer's disease and frontotemporal dementia. Brain.

[71] Vonk JMJ (2020). Semantic loss marks early Alzheimer's disease-related neurodegeneration in older adults without dementia. Alzheimers Dement (Amst)..

[72] Blanc F (2015). Cortical thickness in dementia with lewy bodies and Alzheimer's disease: A comparison of prodromal and dementia stages. PLoS ONE.

[73] Ash S (2012). Impairments of speech fluency in Lewy body spectrum disorder. Brain Lang..

[74] Lee SH (2015). Tract-based analysis of white matter degeneration in Alzheimer's disease. Neuroscience.

[75] Crespi C (2020). Diffusion tensor imaging evidence of corticospinal pathway involvement in frontotemporal lobar degeneration. Cortex.

[76] Delli Pizzi S (2015). Structural Connectivity is differently altered in dementia with Lewy body and Alzheimer's disease. Front. Aging Neurosci..

[77] Kiuchi K (2011). White matter changes in dementia with Lewy bodies and Alzheimer's disease: A tractography-based study. J. Psychiatr. Res..

[78] Watson R (2012). Characterizing dementia with Lewy bodies by means of diffusion tensor imaging. Neurology.

[79] Bozzali M (2005). Brain tissue damage in dementia with Lewy bodies: An in vivo diffusion tensor MRI study. Brain.

[80] Zhang Y (2009). White matter damage in frontotemporal dementia and Alzheimer's disease measured by diffusion MRI. Brain.

[81] Lillo P (2012). Grey and white matter changes across the amyotrophic lateral sclerosis-frontotemporal dementia continuum. PLoS ONE.

[82] Josephs KA (2013). Corticospinal tract degeneration associated with TDP-43 type C pathology and semantic dementia. Brain.

[83] Bzdok D, Krzywinski M, Altman N (2017). Machine learning: A primer. Nat. Methods.

[84] Bzdok D, Altman N, Krzywinski M (2018). Statistics versus machine learning. Nat. Methods.

[85] Tanveer M (2020). Machine learning techniques for the diagnosis of Alzheimer’s disease: A review. ACM Trans. Multimed. Comput. Commun. Appl..

[86] Koikkalainen J (2016). Differential diagnosis of neurodegenerative diseases using structural MRI data. Neuroimage Clin..

[87] Tong T (2017). Five-class differential diagnostics of neurodegenerative diseases using random undersampling boosting. Neuroimage Clin..

[88] Jack CR (2018). NIA-AA research framework: Toward a biological definition of Alzheimer's disease. Alzheimers Dement..

[89] Garcia-Dias R (2020). Neuroharmony: A new tool for harmonizing volumetric MRI data from unseen scanners. Neuroimage.

