# Author Correction: Differential diagnosis of neurodegenerative dementias with the explainable MRI based machine learning algorithm MUQUBIA

**DOI:** 10.1038/s41598-024-51435-7

**Published:** 2024-01-18

**Authors:** Silvia De Francesco, Claudio Crema, Damiano Archetti, Cristina Muscio, Robert I. Reid, Anna Nigri, Maria Grazia Bruzzone, Fabrizio Tagliavini, Raffaele Lodi, Egidio D’Angelo, Brad Boeve, Kejal Kantarci, Michael Firbank, John-Paul Taylor, Pietro Tiraboschi, Alberto Redolfi, the RIN – Neuroimaging Network, Maria Grazia Bruzzone, Pietro Tiraboschi, Claudia A. M. Gandini Wheeler-Kingshott, Michela Tosetti, Gianluigi Forloni, Alberto Redolfi, Egidio D’Angelo, Fabrizio Tagliavini, Raffaele Lodi, Raffaele Agati, Marco Aiello, Elisa Alberici, Carmelo Amato, Domenico Aquino, Filippo Arrigoni, Francesca Baglio, Laura Biagi, Lilla Bonanno, Paolo Bosco, Francesca Bottino, Marco Bozzali, Nicola Canessa, Chiara Carducci, Irene Carne, Lorenzo Carnevale, Antonella Castellano, Carlo Cavaliere, Mattia Colnaghi, Valeria Elisa Contarino, Giorgio Conte, Mauro Costagli, Greta Demichelis, Silvia De Francesco, Andrea Falini, Stefania Ferraro, Giulio Ferrazzi, Lorenzo Figà Talamanca, Cira Fundarò, Simona Gaudino, Francesco Ghielmetti, Ruben Gianeri, Giovanni Giulietti, Marco Grimaldi, Antonella Iadanza, Matilde Inglese, Maria Marcella Laganà, Marta Lancione, Fabrizio Levrero, Daniela Longo, Giulia Lucignani, Martina Lucignani, Maria Luisa Malosio, Vittorio Manzo, Silvia Marino, Jean Paul Medina, Edoardo Micotti, Claudia Morelli, Cristina Muscio, Antonio Napolitano, Anna Nigri, Francesco Padelli, Fulvia Palesi, Patrizia Pantano, Chiara Parrillo, Luigi Pavone, Denis Peruzzo, Nikolaos Petsas, Anna Pichiecchio, Alice Pirastru, Letterio S. Politi, Luca Roccatagliata, Elisa Rognone, Andrea Rossi, Maria Camilla Rossi-Espagnet, Claudia Ruvolo, Marco Salvatore, Giovanni Savini, Emanuela Tagliente, Claudia Testa, Caterina Tonon, Domenico Tortora, Fabio Maria Triulzi

**Affiliations:** 1 Laboratory of Neuroinformatics, IRCCS Istituto Centro San Giovanni di Dio Fatebenefratelli, Brescia, Italy; 2 ASST Bergamo Ovest, Bergamo, Italy; 3 Division of Neurology V/Neuropathology, Fondazione IRCCS Istituto Neurologico Carlo Besta, Milan, Italy; 4 Department of Information Technology, Mayo Clinic and Foundation, Rochester, Minnesota USA; 5 Department of Neuroradiology, Fondazione IRCCS Istituto Neurologico Carlo Besta, Milan, Italy; 6 Scientific Directorate, Fondazione IRCCS Istituto Neurologico Carlo Besta, Milan, Italy; 7 Department of Biomedical and Neuromotor Sciences, University of Bologna, Bologna, Italy; 8 IRCCS Istituto delle Scienze Neurologiche di Bologna, Bologna, Italy; 9 Department of Brain and Behavioral Sciences, University of Pavia, Pavia, Italy; 10 IRCCS Mondino Foundation, Pavia, Italy; 11 Department of Neurology, Mayo Clinic, Rochester, Minnesota USA; 12 Department of Radiology, Mayo Clinic, Rochester, Minnesota USA; 13 Translational and Clinical Research Institute, Newcastle University, Campus for Ageing and Vitality, Newcastle Upon Tyne, UK; 14 Queen Square MS Center, Department of Neuroinflammation, UCL Institute of Neurology, London, UK; 15 University of Pavia, Pavia, Italy; 16 IRCCS Stella Maris, Pisa, Italy; 17 Istituto di Ricerche Farmacologiche Mario Negri IRCCS, Milan, Italy; 18 IRCCS SYNLAB SDN, Naples, Italy; 19 IRCCS Istituti Clinici Scientifici Maugeri, Pavia, Italy; 20 Oasi Research Institute-IRCCS, Troina, Italy; 21 Scientific Institute, IRCCS E. Medea, Milan, Italy; 22 Fondazione IRCCS Istituto Neurologico Carlo Besta, Milan, Italy; 23 IRCCS Fondazione Don Carlo Gnocchi ONLUS, Milan, Italy; 24 IRCCS Centro Neurolesi Bonino Pulejo, Messina, Italy; 25 IRCCS Istituto Ospedale Pediatrico Bambino Gesù, Rome, Italy; 26 Fondazione IRCCS Santa Lucia, Rome, Italy; 27 IRCCS Neuromed, Pozzilli, Italy; 28 IRCCS Ospedale San Raffaele, Milan, Italy; 29 IRCCS Istituto Auxologico Italiano, Milan, Italy; 30 Fondazione IRCCS Ca’ Granda Osp. Maggiore Policlinico, Milan, Italy; 31 University of Genoa, Genoa, Italy; 32 Philips Healthcare, Milan, Italy; 33 Fondazione Policlinico Universitario Agostino Gemelli IRCCS, Rome, Italy; 34 IRCCS Humanitas Research Hospital, Rozzano, Italy; 35 IRCCS Ospedale Policlinico San Martino, Genoa, Italy; 36 IRCCS Istituto Giannina Gaslini, Genoa, Italy; 37 University of Turin, Turin, Italy

Correction to: *Scientific Reports* 10.1038/s41598-023-43706-6, published online 13 October 2023

In the original version of this Article the consortia names were incorrectly given as “for the ADNI, Frontotemporal Lobar Degeneration Neuroimaging; NIA Alzheimer’s Disease Centers; and the RIN – Neuroimaging Network”. The correct consortium name is “the RIN – Neuroimaging Network”.

The original Article has been corrected.

