# Supplementary material for: Differential diagnosis of neurodegenerative dementias with the explainable MRI based machine learning algorithm MUQUBIA
Source: Sci Rep. 2023 Oct 13;13:17355. doi: 10.1038/s41598-023-43706-6 (PMC10575864; doi:10.1038/s41598-023-43706-6)
Supplement: Supplementary file 1 — Supplementary Information. [file 41598_2023_43706_MOESM1_ESM.docx]

**SUPPLEMENTARY MATERIAL**

**Differential diagnosis of neurodegenerative dementias with the explainable MRI based machine learning algorithm MUQUBIA**

*Silvia De Francesco^1*^, Claudio Crema^1^, Damiano Archetti^1^, Cristina Muscio^2^, Robert I. Reid^3^, Anna Nigri*^4^*, Maria Grazia Bruzzone^4^, Fabrizio Tagliavini*^5^*, Raffaele Lodi^6,7^, Egidio D’Angelo^8,9^, Brad Boeve^10^, Kejal Kantarci^11^, Michael Firbank^12^, John-Paul Taylor^12^, Pietro Tiraboschi^13^, Alberto Redolfi^1^* & the RIN – Neuroimaging Network^**^.


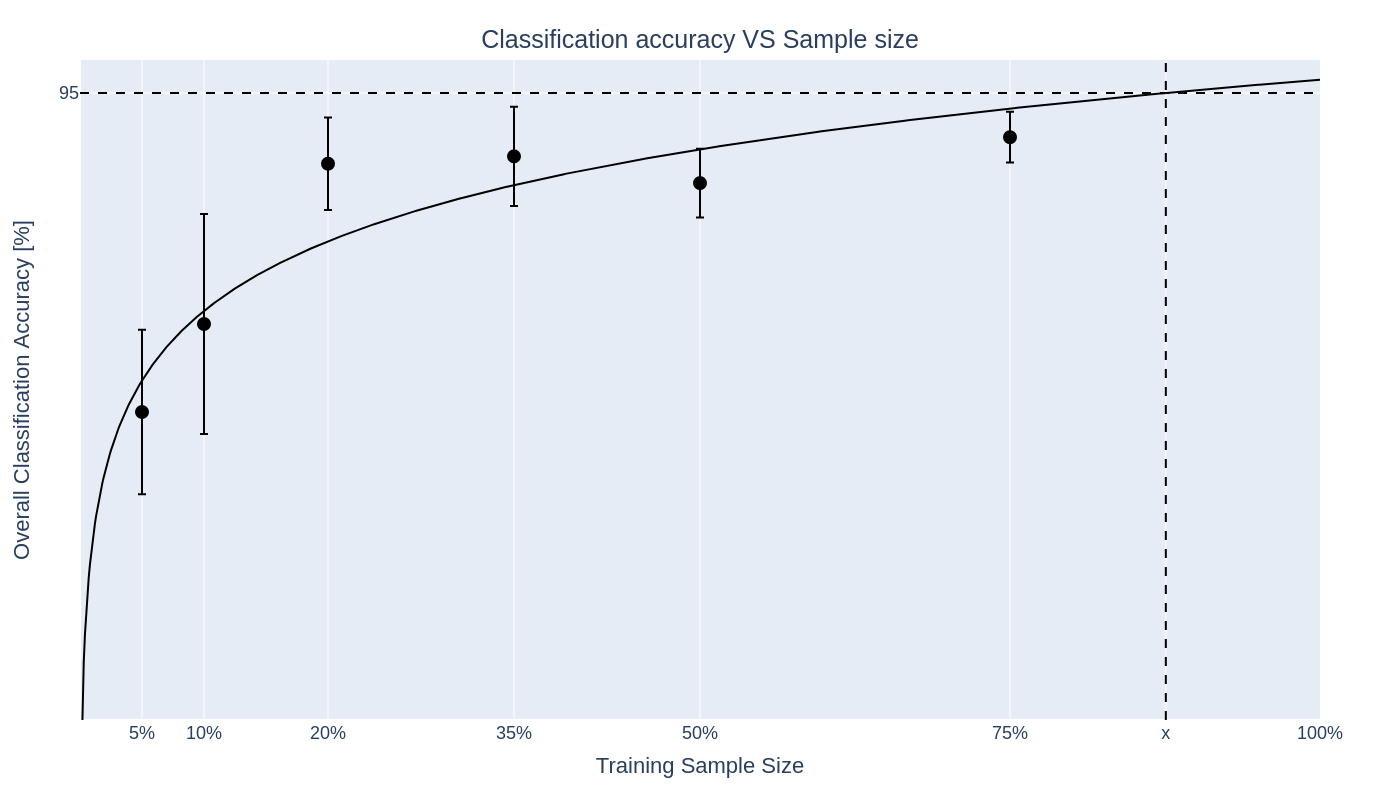


**Figure Sup 1**: Relationship between training sample size and classification accuracy. The whole training sample was composed by 354 subjects with 22 features. The first point represents the 5% of it (17 subjects and 1 feature, reduced accordingly to avoid over-fitting), the last point represents the 75% (265 subjects and 16 features). X represents the percentage at which the accuracy can reach the value of 95%; this point corresponds to the 87.6% of the whole size. This result demonstrates that the training sample considered was large enough to achieve a good model performance.


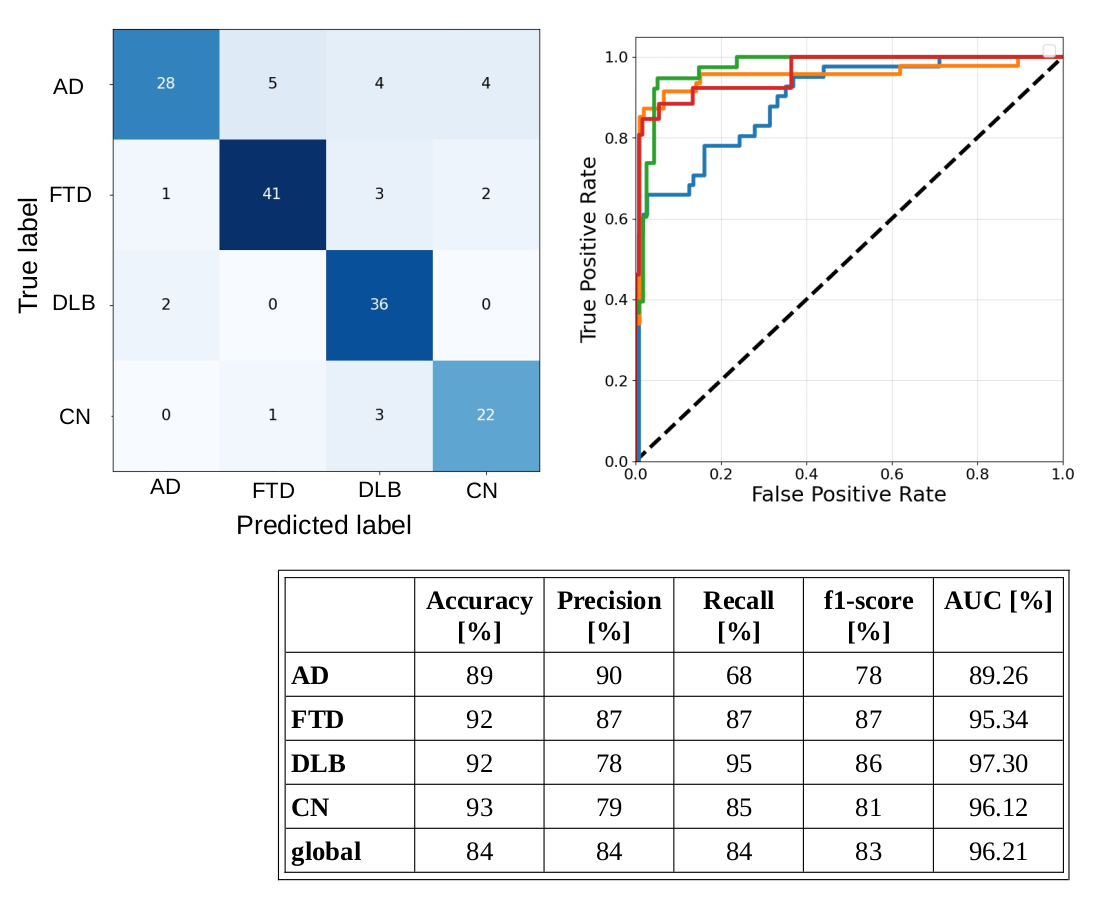
**Figure Sup 2**: Confusion matrix, ROC curves, and performance metrics of the test set using the MUQUBIA model excluding CDR from the set of features. Global metrics are macro-averaged, except for the accuracy which was calculated with the multiclass approach (sum of diagonal divided by all records). Acronyms: AD, Alzheimer’s Dementia; FTD, Frontotemporal Disease; DLB, Dementia with Lewy Body; CN, Cognitive Normal; AUC, Area Under the Curve.



**Figure Sup 3**: The figure shows a matrix of correctly classified subjects (on the diagonal) and some randomly selected subjects from the test set that were incorrectly predicted by MUQUBIA. The influence of the features on the correct and incorrect class predictions was examined. Each subgraph contains an output value *f(x)*, which was the final prediction of the model, and an expected value (E[*f(x)*]=1.67), which was the mean of all MUQUBIA predictions for the test set. Features that shifted the prediction upward (to the right) are shown in purple, and those that shifted the prediction downward (to the left) are shown in blue.

For the true positive case in AD, almost all features had a similar impact (in blue) in the direction of *f(x)=0*, except for left cortex volume, which had a weak opposite influence (in purple) on the final MUQUBIA decision. The prediction for the true positive case in the FTD class, denoted by *f(x)=1*, had many features with a similar impact (in blue), but some features had an opposite effect (i.e.: left cortex volume, left lateral orbitofrontal volume). The prediction of true positive DLB (*f(x)=2)* had increasingly more features with purple colors (i.e.: left corticospinal tract FA, left cortex volume, right retrolenticular part of the internal capsule MD, right medial lemniscus MD, and volume of the left putamen), but other features had a smaller opposite influence. For the true CN case, denoted by *f(x)=3*, the CDR and age features had a significant influence that greatly increased the probability of belonging to the true class. On the other hand, for all the incorrectly predicted subjects in the matrix, the impacts of the features had confounded MUQUBIA. Some of the features had a slight effect in favor of the true class, but other features had a higher overall opposite effect, so the probability of being in the true class ends up to be too low and a classification error occurred. Acronyms: AD: Alzheimer’s Dementia, FTD: Frontotemporal Dementia, DLB: Dementia with Lewy bodies, CN: Cognitively Normal, FA: Fractional Anisotropy, MD: Mean Diffusivity, lh or L: left, rh or R: right*,* //: No errors reported in the test set group.


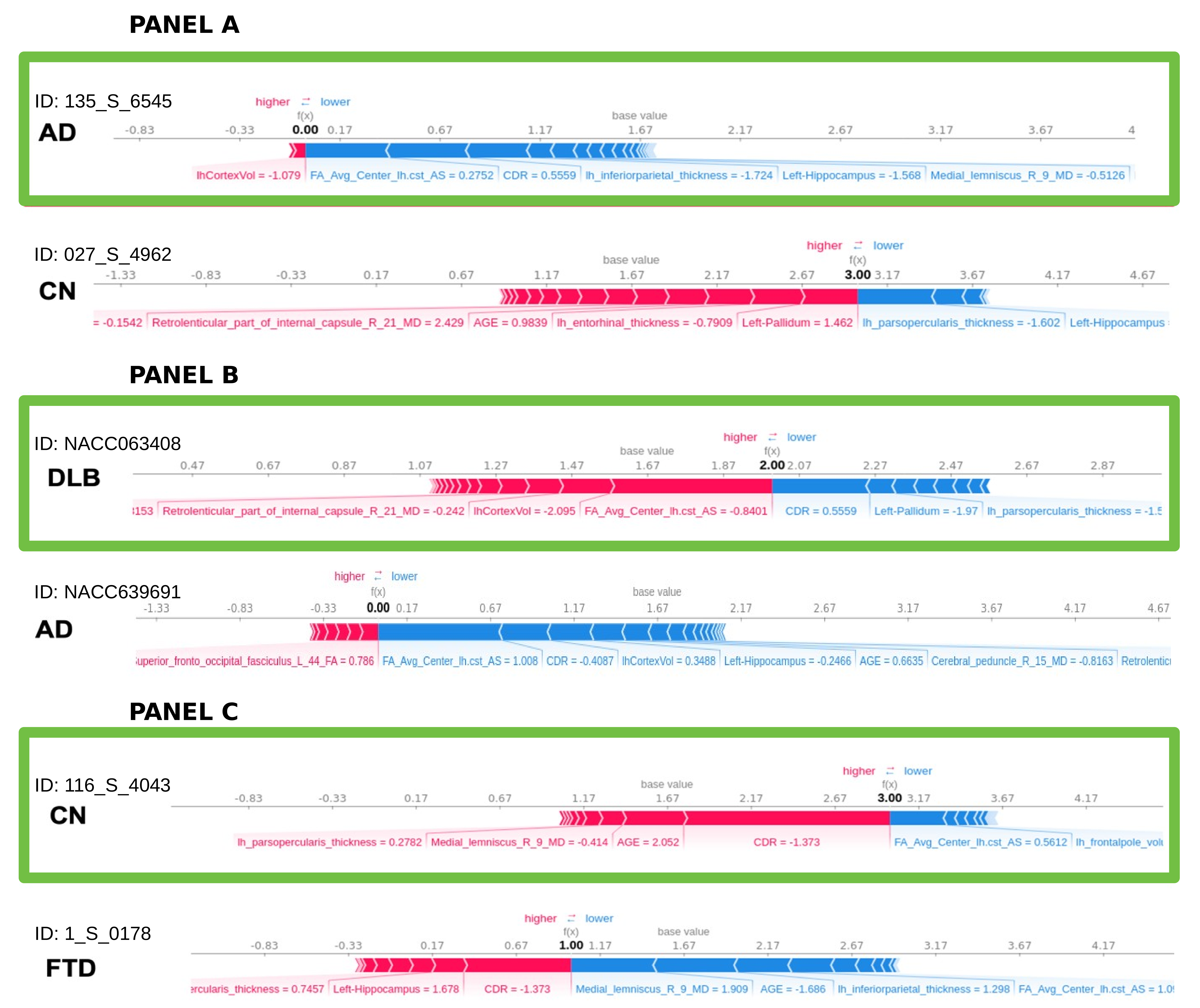
**Figure Sup 4**: Various ‘force plots’ are shown. This is an effective representation showing how MUQUBIA arrived at its decision. Panel A shows two patients with a clinical diagnosis of AD. The first (green rectangle) was correctly classified by MUQUBIA as AD while the second was misclassified as CN. The plot shows the "base value" (also known as expected value (E[*f(x)*]=1.67), i.e.: the mean value derived from all the MUQUBIA predictions on the test set) and the output value (*f(x)*), which was the final prediction of MUQUBIA for that specific patient. In this case the difference between the probability for the predicted class and the true class was very large. In addition to the left pallidum, the left entorhinal cortex, age, and the right retrolenticular part of the internal capsule also played an important role in increasing the CN probability. Panel B shows two patients with a clinical diagnosis of DLB. The first (green rectangle) was correctly classified as DLB, while the second was misclassified as AD. It can be seen that the difference between the probability for the predicted class and the true class was quite large. It appears that most of the contribution of the corticospinal tract, CDR and other ROI features, such as volume of the left cortex and partially left hippocampus, artfully pointed to AD rather than DLB. Panel C shows two CN subjects. The first (green rectangle) was correctly classified as CN, while the second was misclassified as FTD. It can be seen that the difference between the probability for the predicted class and the true class was quite large. It appears that the age and many diffusivity features, such as the medial lemniscus and left corticospinal tract, in addition to the thickness values of the inferior parietal region and age, were incorrectly more suggestive of FTD than CN. Acronyms: AD: Alzheimer’s Dementia, FTD: Frontotemporal Dementia, DLB: Dementia with Lewy bodies, CN: Cognitively Normal, base value: E[*f(x)*], FA: Fractional Anisotropy, MD: Mean Diffusivity, lh or L: left, rh or R: right.


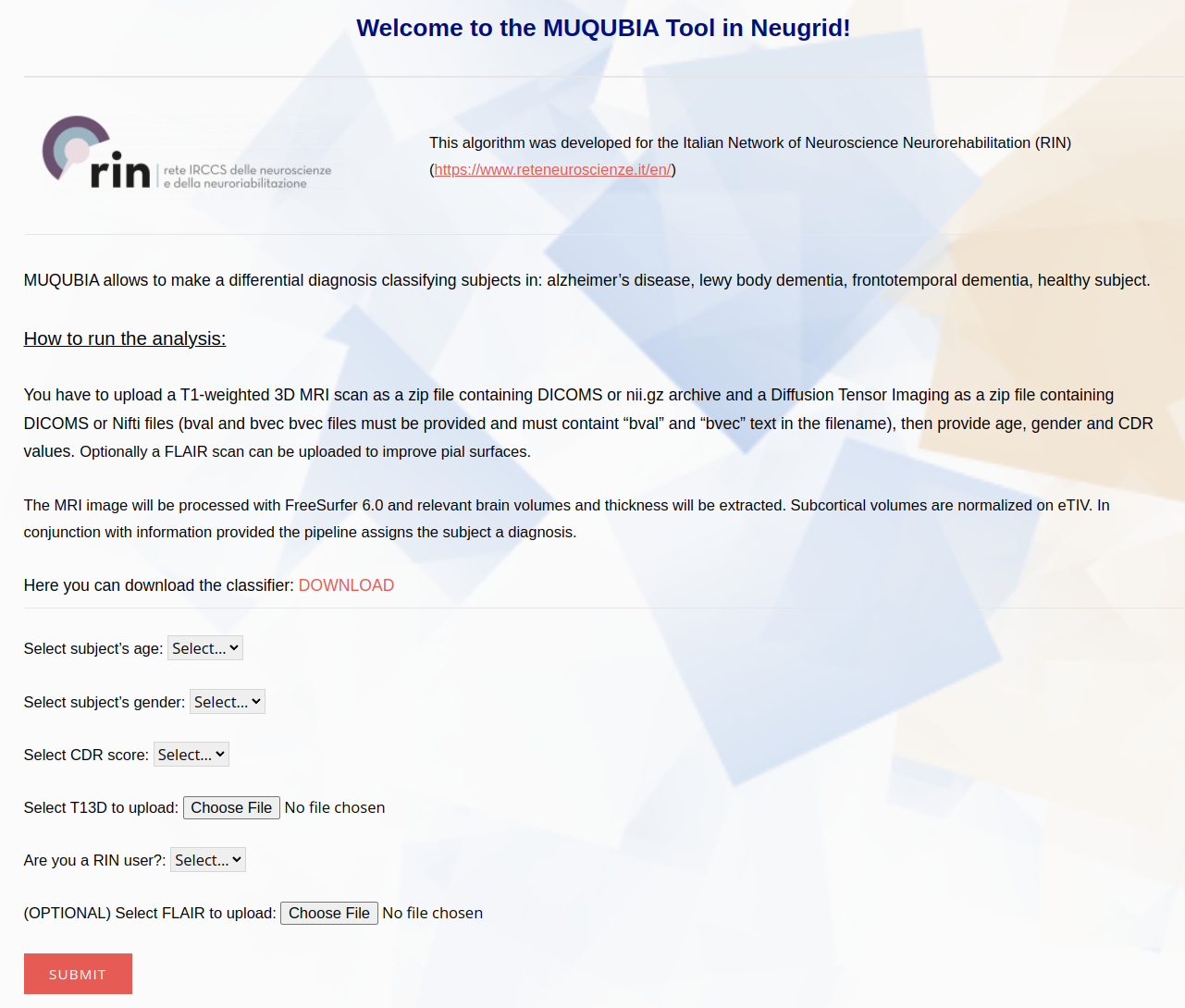
**Figure Sup 5**: Interface of the MUQUBIA pipeline in the neuGRID platform. The user must enter the information required by the classifier (age, gender, T13D and DTI images and optionally the CDR and FLAIR scan). The MUQUBIA pipeline runs on the neuGRID servers (https://neugrid2.eu) to produce a PDF report that is sent to the user’s mailbox (see Supplementary Figure 5). The web page includes a link to download the two classifiers so that users can also run MUQUBIA independently on local resources.


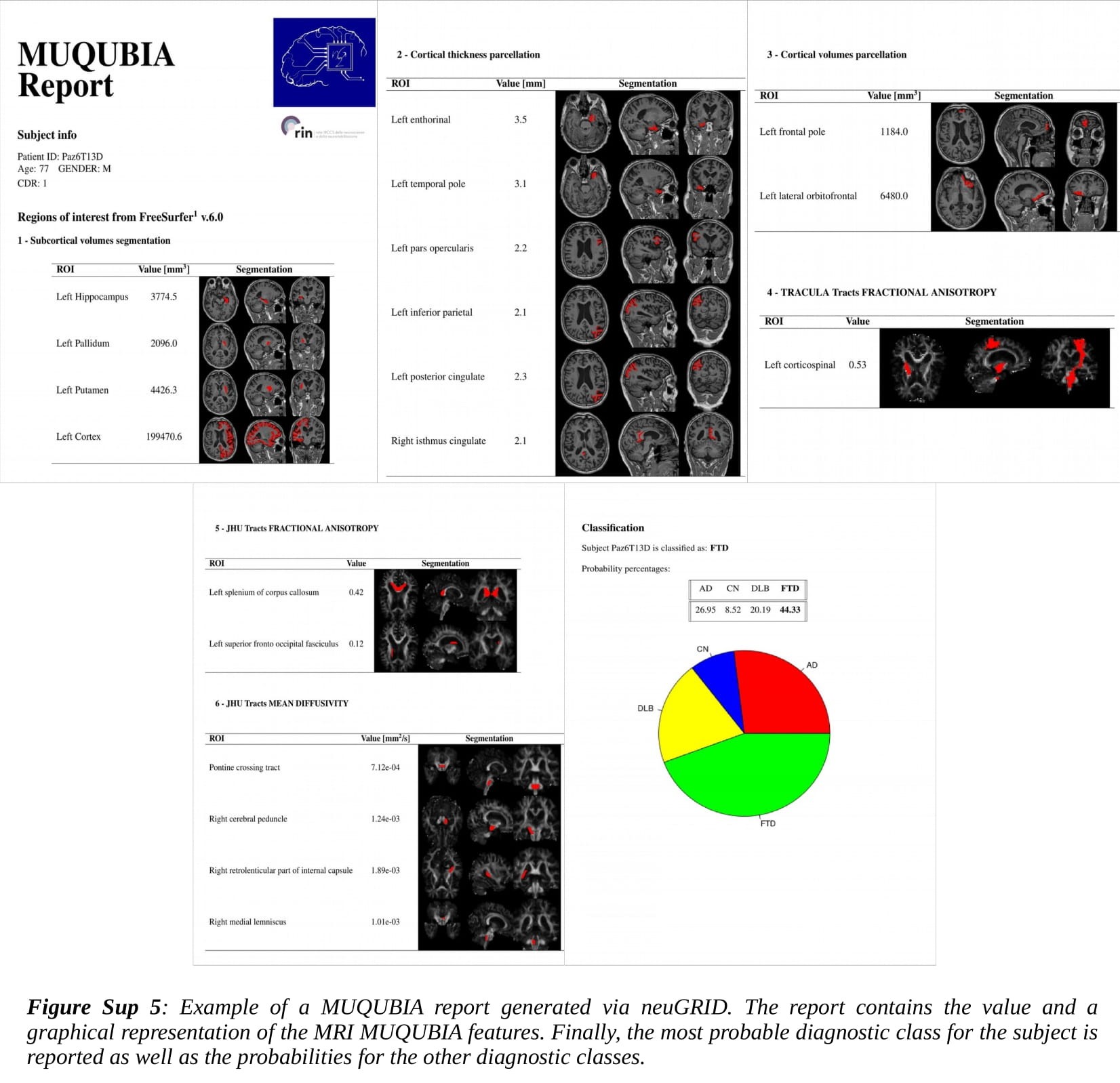


**Figure Sup 6**: Example of a MUQUBIA report generated via neuGRID (https://neugrid2.eu). The report includes the value and graphical representation of MRI MUQUBIA features organized into six main sections (i.e.: subcortical volumes, cortical thickness, cortical volumes, fractional anisotropy and mean diffusivity measurements). Finally, the most probable diagnostic class for the subject is returned, as well as the probabilities for the other diagnostic classes.


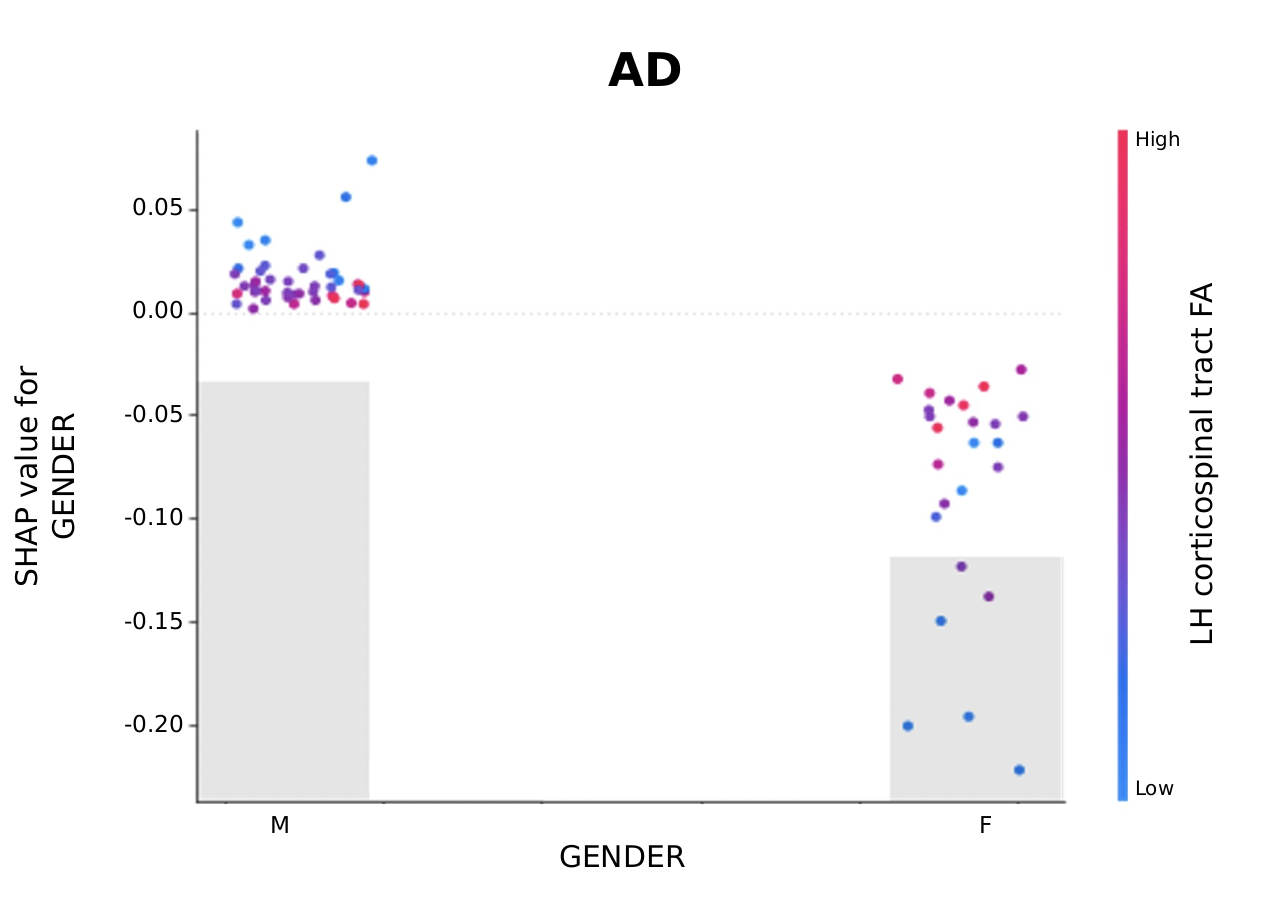
**Figure Sup 7**: Influence of gender on MUQUBIA classification in AD patients. In women, degeneration of the left corticospinal tract had a progressive and greater effect on final classification, which was not the case in men. Acronyms: AD: Alzheimer’s Dementia, FA: Fractional Anisotropy, LH: left hemisphere, M: Male, F: Female.


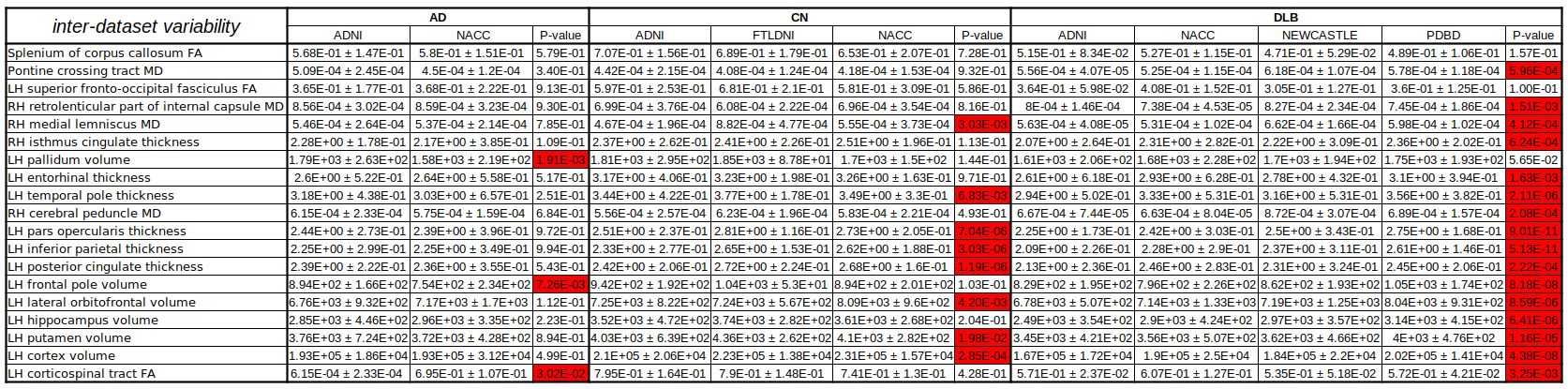


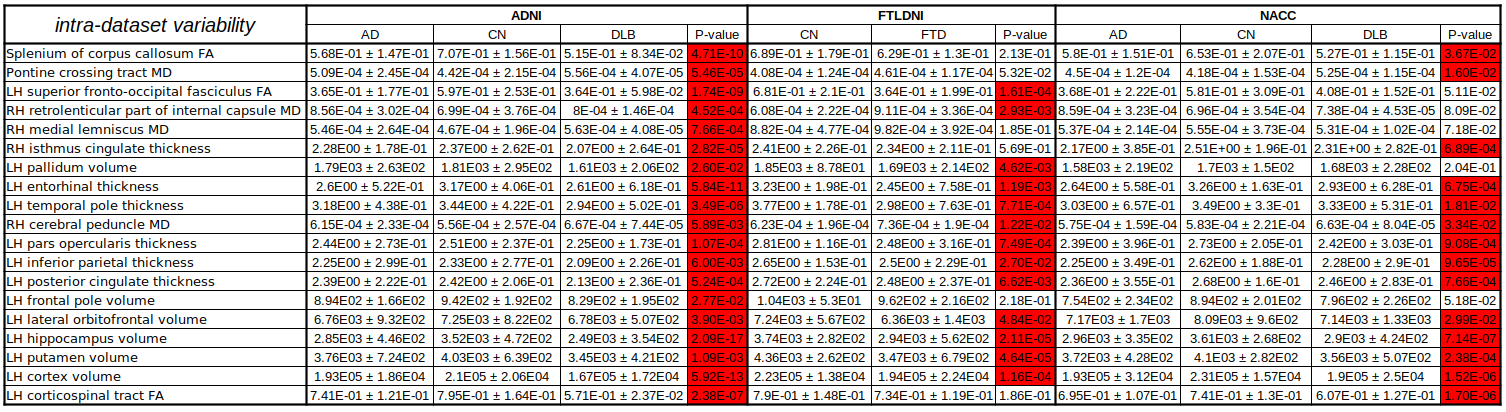


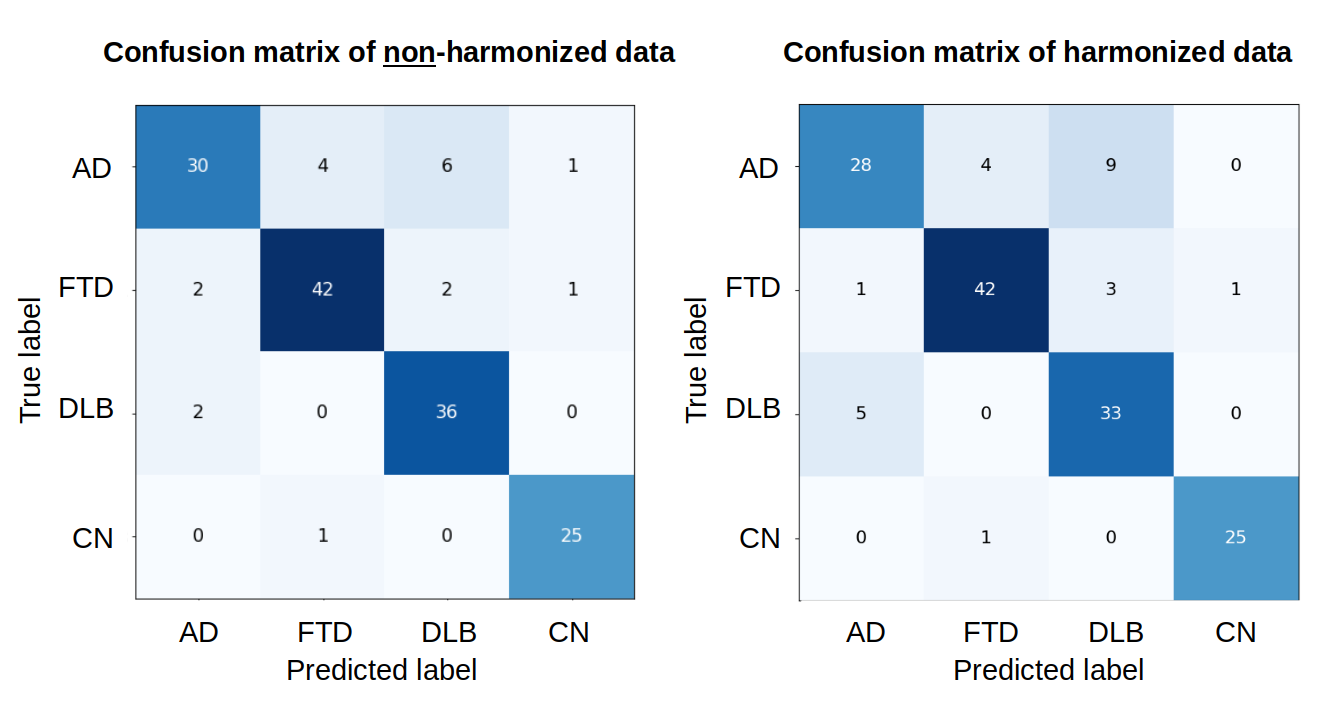
**Figure Sup 8:** Inter- and intra- cohort variability of the MRI features selected by MUQUBIA. The two tables contain the means and standard deviations of the MRI features selected by the MUQUBIA algorithm and the p-values determined with the Kruskal-Wallis test for the comparison of the same diagnostic group from different datasets in the case of inter-cohort variability and of the different diagnostic classes from the same dataset in the case of intra-cohort variability. In the inter-cohort table, the distributions of the features were significantly different (p-value < 0.05, red cells) in 47% of cases, while in the case of diagnostic intra-cohort variability distributions were significantly different in 81% of cases. This suggests that pathology-related differences were greater than the potential bias from the acquisition protocols used. The robustness of the MUQUBIA algorithm to potential differences in MRI acquisition was further confirmed by the similarity of the classifier’s confusion matrix trained with non-harmonized data or data harmonized with the COMBAT tool [10].

**Supplementary Table S1**: Diagnostic, selection, and exclusion criteria for each group and database

|  | ADNI | FTLDNI | NACC | PDBP | Newcastle |
| --- | --- | --- | --- | --- | --- |
| AD | -**Diagnostic criteria**:  McKhann et al. 1984 [1]  McKhann et al. 2011 [2]  -**Selection criteria**:  Age from 55 to 90 years.  Fluent in English or Spanish.  Permitted medications. Stable for at least 4 weeks prior to screening.  Adequate visual and auditory acuity.  No additional diseases.  Completed 6^th^ grades of education or sufficient work history to exclude mental retardation.  Modified Hachinski score <= 4.  GDS < 6.  Memory complaints by patient or study partner.  Abnormal memory function score on the Wechsler Memory Scale (adjusted for education).  MMSE between 20 and 26.  CDR >= 0.5  Memory Box score >= 1.0.  -**Exclusion criteria**:  Any significant neurological disease other than AD.  Abnormal baseline MRI.  Presence of pacemakers, aneurysm clips, artificial heart valves, ear implants, metal fragments or foreign objects in the eyes, skin, or body.  Major depression, bipolar disorder, history of schizophrenia.  History of alcohol or substance abuse or dependency within the past 2 years.  Psychotic features, agitation or behavioral problems within the past 3 months.  Any significant systemic illness or unstable medical condition.  Clinically significant laboratory abnormalities. | // | -**Diagnostic criteria:**  McKhann et al., 1984 [1]  McKhann et al., 2011 [2]  -**Selection criteria:**  Each Center enrolls its participants according to its own protocol  -**Exclusion criteria**:  Concomitant dementia.  Concurrent neurological disease. Non-neurological medical co-morbidity or medication use that could have impact on cognition. | // | // |
| FTD | // | **-Diagnostic criteria**:  Rascovsky et al., 2011 [4];  Gorno-Tempini et al., 2011 [5]  **-Selection criteria:**  Age between 45 and 90 years.  Able to undergo testing procedures, including neuroimaging, and agree to longitudinal follow-up.  Need a reliable study partner.  **-Exclusion criteria:**  Any significant neurological disease other than FTD.  Any significant systemic illness or unstable medical condition.  Presence of pacemakers, aneurysm clips, artificial heart valves, ear implants, metal fragments or foreign objects in the eyes, skin or body. Long-standing (>10 years) history of alcohol or substance abuse.  Long-standing (>10 years) history of major depression, bipolar disorder or schizophrenia.  Clinically significant abnormalities in B12, RPR or TFTs | // | // | // |
| DLB | **-Diagnostic criteria:**  Montine et al., 2012 [3]  **-Selection criteria:**  Not missing neuritic plaque burden or Braak neurofibrillary tangle stage.  -**Exclusion criteria**:  NA | // | -**Diagnostic criteria:**  McKeith et al., 2005 [6]  McKeith et al., 2017 [7]  -**Selection criteria:**  Each Center enrolls its participants according to its own protocol  -**Exclusion criteria**:  NA | **-Diagnostic criteria**:  McKeith et al., 2017 [7]  -**Selection criteria**:  AGE ≥ 18 years old.  Not using investigational drugs or devices within 60 days prior to baseline visit.  No major psychiatric disorder currently or by history.  Able to participate in study activities including all required clinical assessments and biological donations.  No contraindication to lumbar puncture.  No contraindication to MRI imaging.  -**Exclusion criteria**:  Presence of another neurologic disorder.  Unwilling to return for follow-up yearly and undergo neuropsychological testing and MR imaging.  If undergoing Tau imaging cannot have QT Prolongation.  Do not have a reliable informant. | -**Diagnostic criteria**:  McKeith et al., 1996 [8]  McKeith et al., [2005](https://onlinelibrary.wiley.com/doi/10.1002/hbm.23100#hbm23100-bib-0057) [6]  McKeith et al., 2017 [7]  -**Selection criteria**:  AGE ≥ 60 years old  MMSE > 10  -**Exclusion criteria**:  contraindications for PET-CT or MRI, moderate to severe visual impairment, previous history of alcohol or substance misuse, significant neurological or psychiatric history, moderate to severe cerebral small vessel disease, focal brain lesions on brain imaging; severe concurrent illness. |
| CN | **-Selection criteria**:  Free of memory complaints.  Normal memory function score on the Wechsler Memory Scale (adjusted for education).  MMSE between 24 and 30.  CDR = 0. Memory Box score = 0.  Absence of impairment in cognitive functions.  Stability of Permitted Medications for 4 weeks. GDS < 6.  Age from 55 to 90 years.  Adequate visual and auditory acuity.  Good general health.  Not pregnant, lactating, or of childbearing potential (two years post-menopausal or surgically sterile).  Modified Hachinski score <=4.  Completed 6^th^ grades of education or sufficient work history to exclude mental retardation.  Fluent in English or Spanish.  **-Exclusion criteria**:  Any significant neurologic disease.  Abnormal baseline MRI. Multiple lacunes or lacunes in a critical memory structure.  Presence of pacemakers, aneurysm clips, artificial heart valves, ear implants, metal fragments or foreign objects in the eyes, skin, or body.  Major depression, bipolar disorder, history of schizophrenia.  History of alcohol or substance abuse or dependency within the past 2 years.  Psychotic features, agitation or behavioral problems within the past 3 months.  Any significant systemic illness or unstable medical condition.  Clinically significant laboratory abnormalities. | **-Selection criteria:**  Age between 40 and 80.  No known diagnosis of a neurological or neurodegenerative condition.  No known history of memory complaints.  Must be able to walk five steps with minimal assistance.  **-Exclusion criteria**:  Any significant neurological disease.  History of significant head trauma or known structural abnormalities. Any significant systemic illness or unstable/uncontrolled medical condition.  The presence of pacemakers, aneurysm clips, artificial heart valves, ear implants, or metal fragments or metal objects in the eyes, skin, or body.  No longstanding history of alcohol or substance abuse, major depression, bipolar disorder or schizophrenia.  No clinically significant abnormalities in B12, RPR or TFTs. | **-Selection criteria:**  CDR = 0.  Neuorpshycological testing within the normal range.  Normal behavior.  **-Exclusion criteria**:  Presence of MCI, dementia, or non-MCI impairment. | // | // |

*The table reports all the inclusion and exclusion criteria adopted by each database to enroll subjects and perform the diagnosis. Acronyms: GDS, Geriatric Depression Scale; MMSE, Mini Mental State Examination; CDR, Clinical Dementia Rating Dementia Staging Instrument; AD, Alzheimer’s Dementia; FTD, Frontotemporal Dementia; DLB, Dementia with Lewy Bodies; MCI, Mild Cognitive Impairment; MRI, Magnetic Resonance Imaging; NA, Not Available; PET-CT, Positron Emission Tomography Computed Tomography; B12, Vitamin B12; RPR, Rapid Plasma Reagin; TFT, Thyroid Function Test.*

**Supplementary Table S2:** The complete dataset composed by subjects collected from 5 databases and sequence availability.

| N° | **ID** | **DIAGNOSIS** | **DATASET** | **Autoptic assessment** | **T13D** | **FLAIR** | **DTI** |
| --- | --- | --- | --- | --- | --- | --- | --- |
| 1 | 003_S_4136 | AD | ADNI | x | v | v | v |
| 2 | 003_S_4152 | AD | ADNI | x | v | v | v |
| 3 | 003_S_4373 | AD | ADNI | x | v | v | v |
| 4 | 003_S_4892 | AD | ADNI | x | v | v | v |
| 5 | 003_S_5165 | AD | ADNI | x | v | v | v |
| 6 | 003_S_5187 | AD | ADNI | x | v | v | v |
| 7 | 005_S_4707 | AD | ADNI | x | v | v | v |
| 8 | 005_S_4910 | AD | ADNI | x | v | v | v |
| 9 | 005_S_5038 | AD | ADNI | x | v | v | v |
| 10 | 005_S_5119 | AD | ADNI | x | v | v | v |
| 11 | 006_S_6689 | AD | ADNI | x | v | v | v |
| 12 | 007_S_4568 | AD | ADNI | x | v | v | v |
| 13 | 007_S_5196 | AD | ADNI | x | v | v | v |
| 14 | 011_S_4827 | AD | ADNI | x | v | v | v |
| 15 | 011_S_6303 | AD | ADNI | x | v | v | v |
| 16 | 013_S_6768 | AD | ADNI | x | v | v | v |
| 17 | 016_S_4009 | AD | ADNI | x | v | v | v |
| 18 | 016_S_4353 | AD | ADNI | x | v | v | v |
| 19 | 016_S_4591 | AD | ADNI | x | v | v | v |
| 20 | 016_S_4887 | AD | ADNI | x | v | v | v |
| 21 | 016_S_4963 | AD | ADNI | x | v | v | v |
| 22 | 016_S_5032 | AD | ADNI | x | v | v | v |
| 23 | 016_S_5057 | AD | ADNI | x | v | v | v |
| 24 | 016_S_5251 | AD | ADNI | x | v | v | v |
| 25 | 016_S_6839 | AD | ADNI | x | v | v | v |
| 26 | 021_S_4924 | AD | ADNI | x | v | v | v |
| 27 | 022_S_6013 | AD | ADNI | x | v | v | v |
| 28 | 022_S_6796 | AD | ADNI | x | v | v | v |
| 29 | 023_S_6661 | AD | ADNI | x | v | v | v |
| 30 | 027_S_4801 | AD | ADNI | x | v | v | v |
| 31 | 027_S_4938 | AD | ADNI | v | v | v | v |
| 32 | 027_S_4962 | AD | ADNI | v | v | v | v |
| 33 | 027_S_4964 | AD | ADNI | x | v | v | v |
| 34 | 027_S_6648 | AD | ADNI | x | v | v | v |
| 35 | 027_S_6733 | AD | ADNI | x | v | v | v |
| 36 | 027_S_6849 | AD | ADNI | x | v | v | v |
| 37 | 029_S_4307 | AD | ADNI | x | v | v | v |
| 38 | 032_S_6600 | AD | ADNI | x | v | v | v |
| 39 | 033_S_6705 | AD | ADNI | x | v | v | v |
| 40 | 033_S_6824 | AD | ADNI | x | v | v | v |
| 41 | 035_S_6650 | AD | ADNI | x | v | v | v |
| 42 | 036_S_6179 | AD | ADNI | x | v | v | v |
| 43 | 036_S_6231 | AD | ADNI | x | v | v | v |
| 44 | 052_S_4959 | AD | ADNI | x | v | v | v |
| 45 | 052_S_5062 | AD | ADNI | x | v | v | v |
| 46 | 052_S_6305 | AD | ADNI | x | v | v | v |
| 47 | 094_S_4089 | AD | ADNI | x | v | v | v |
| 48 | 094_S_4282 | AD | ADNI | x | v | v | v |
| 49 | 094_S_4737 | AD | ADNI | x | v | v | v |
| 50 | 098_S_4201 | AD | ADNI | x | v | v | v |
| 51 | 098_S_4215 | AD | ADNI | x | v | v | v |
| 52 | 098_S_6601 | AD | ADNI | x | v | v | v |
| 53 | 098_S_6658 | AD | ADNI | x | v | v | v |
| 54 | 100_S_6713 | AD | ADNI | x | v | v | v |
| 55 | 109_S_4378 | AD | ADNI | x | v | v | v |
| 56 | 114_S_6595 | AD | ADNI | x | v | v | v |
| 57 | 116_S_6100 | AD | ADNI | x | v | v | v |
| 58 | 116_S_6543 | AD | ADNI | x | v | v | v |
| 59 | 126_S_4494 | AD | ADNI | x | v | v | v |
| 60 | 126_S_4686 | AD | ADNI | x | v | v | v |
| 61 | 126_S_6683 | AD | ADNI | x | v | v | v |
| 62 | 126_S_6721 | AD | ADNI | x | v | v | v |
| 63 | 127_S_4500 | AD | ADNI | x | v | v | v |
| 64 | 127_S_4940 | AD | ADNI | x | v | v | v |
| 65 | 127_S_4992 | AD | ADNI | x | v | v | v |
| 66 | 127_S_5056 | AD | ADNI | x | v | v | v |
| 67 | 127_S_5058 | AD | ADNI | v | v | v | v |
| 68 | 127_S_5067 | AD | ADNI | x | v | v | v |
| 69 | 127_S_5095 | AD | ADNI | x | v | v | v |
| 70 | 127_S_6433 | AD | ADNI | x | v | v | v |
| 71 | 127_S_6549 | AD | ADNI | x | v | v | v |
| 72 | 135_S_6389 | AD | ADNI | x | v | v | v |
| 73 | 135_S_6545 | AD | ADNI | x | v | v | v |
| 74 | 135_S_6687 | AD | ADNI | x | v | v | v |
| 75 | 135_S_6840 | AD | ADNI | x | v | v | v |
| 76 | 137_S_6812 | AD | ADNI | x | v | v | v |
| 77 | 168_S_6735 | AD | ADNI | x | v | v | v |
| 78 | 168_S_6754 | AD | ADNI | x | v | v | v |
| 79 | 168_S_6827 | AD | ADNI | x | v | v | v |
| 80 | 168_S_6843 | AD | ADNI | x | v | v | v |
| 81 | 301_S_6592 | AD | ADNI | x | v | v | v |
| 82 | 305_S_6810 | AD | ADNI | x | v | v | v |
| 83 | 305_S_6850 | AD | ADNI | x | v | v | v |
| 84 | 341_S_6820 | AD | ADNI | x | v | v | v |
| 85 | NACC010645 | AD | NACC | x | v | v | v |
| 86 | NACC014159 | AD | NACC | x | v | v | v |
| 87 | NACC047218 | AD | NACC | v | v | v | v |
| 88 | NACC051988 | AD | NACC | x | v | v | v |
| 89 | NACC063353 | AD | NACC | x | v | v | v |
| 90 | NACC065301 | AD | NACC | x | v | v | v |
| 91 | NACC077588 | AD | NACC | x | v | v | v |
| 92 | NACC109608 | AD | NACC | x | v | v | v |
| 93 | NACC128307 | AD | NACC | x | v | v | v |
| 94 | NACC129937 | AD | NACC | x | v | v | v |
| 95 | NACC131130 | AD | NACC | v | v | v | v |
| 96 | NACC133186 | AD | NACC | x | v | v | v |
| 97 | NACC172239 | AD | NACC | x | v | v | v |
| 98 | NACC172271 | AD | NACC | x | v | v | v |
| 99 | NACC177590 | AD | NACC | x | v | v | v |
| 100 | NACC185704 | AD | NACC | x | v | v | v |
| 101 | NACC198306 | AD | NACC | x | v | v | v |
| 102 | NACC221021 | AD | NACC | x | v | v | v |
| 103 | NACC223800 | AD | NACC | x | v | v | v |
| 104 | NACC226634 | AD | NACC | x | v | v | v |
| 105 | NACC233243 | AD | NACC | x | v | v | v |
| 106 | NACC237988 | AD | NACC | x | v | v | v |
| 107 | NACC253417 | AD | NACC | x | v | v | v |
| 108 | NACC253637 | AD | NACC | x | v | v | v |
| 109 | NACC278460 | AD | NACC | x | v | v | v |
| 110 | NACC344247 | AD | NACC | x | v | v | v |
| 111 | AMPLE_A001 | DLB | NEWCASTLE | x | v | v | x |
| 112 | AMPLE_A002 | DLB | NEWCASTLE | x | v | v | x |
| 113 | AMPLE_A003 | DLB | NEWCASTLE | x | v | v | x |
| 114 | AMPLE_A005 | DLB | NEWCASTLE | x | v | v | x |
| 115 | AMPLE_A006 | DLB | NEWCASTLE | x | v | v | x |
| 116 | AMPLE_A007 | DLB | NEWCASTLE | x | v | v | x |
| 117 | AMPLE_A008 | DLB | NEWCASTLE | x | v | v | x |
| 118 | AMPLE_A009 | DLB | NEWCASTLE | x | v | v | x |
| 119 | AMPLE_A010 | DLB | NEWCASTLE | x | v | v | x |
| 120 | AMPLE_A011 | DLB | NEWCASTLE | x | v | v | x |
| 121 | AMPLE_A013 | DLB | NEWCASTLE | x | v | v | x |
| 122 | AMPLE_A014 | DLB | NEWCASTLE | x | v | v | x |
| 123 | AMPLE_A018 | DLB | NEWCASTLE | x | v | v | x |
| 124 | AMPLE_A019 | DLB | NEWCASTLE | x | v | v | x |
| 125 | AMPLE_A020 | DLB | NEWCASTLE | x | v | v | x |
| 126 | AMPLE_A022 | DLB | NEWCASTLE | x | v | v | x |
| 127 | AMPLE_A027 | DLB | NEWCASTLE | x | v | v | x |
| 128 | AMPLE_A028 | DLB | NEWCASTLE | x | v | v | x |
| 129 | AMPLE_A030 | DLB | NEWCASTLE | x | v | v | x |
| 130 | ART_A002 | DLB | NEWCASTLE | x | v | v | v |
| 131 | ART_A005 | DLB | NEWCASTLE | x | v | v | v |
| 132 | ART_A006 | DLB | NEWCASTLE | x | v | v | v |
| 133 | ART_A007 | DLB | NEWCASTLE | x | v | v | v |
| 134 | ART_A008 | DLB | NEWCASTLE | x | v | v | v |
| 135 | ART_A009 | DLB | NEWCASTLE | x | v | v | v |
| 136 | ART_A011 | DLB | NEWCASTLE | x | v | v | v |
| 137 | ART_A013 | DLB | NEWCASTLE | x | v | v | v |
| 138 | ART_A014 | DLB | NEWCASTLE | x | v | v | v |
| 139 | ART_A016 | DLB | NEWCASTLE | x | v | v | v |
| 140 | CAT_A001 | DLB | NEWCASTLE | x | v | x | v |
| 141 | CAT_A002 | DLB | NEWCASTLE | x | v | x | v |
| 142 | CAT_A004 | DLB | NEWCASTLE | x | v | x | v |
| 143 | CAT_A005 | DLB | NEWCASTLE | x | v | x | v |
| 144 | CAT_A006 | DLB | NEWCASTLE | x | v | x | v |
| 145 | CAT_A007 | DLB | NEWCASTLE | x | v | x | v |
| 146 | CAT_A008 | DLB | NEWCASTLE | x | v | x | v |
| 147 | CAT_A011 | DLB | NEWCASTLE | x | v | x | v |
| 148 | CAT_A012 | DLB | NEWCASTLE | x | v | x | v |
| 149 | CAT_A013 | DLB | NEWCASTLE | x | v | x | v |
| 150 | CAT_A014 | DLB | NEWCASTLE | x | v | x | v |
| 151 | CAT_A015 | DLB | NEWCASTLE | x | v | x | v |
| 152 | CAT_A016 | DLB | NEWCASTLE | x | v | x | v |
| 153 | CAT_A018 | DLB | NEWCASTLE | x | v | x | v |
| 154 | CAT_A019 | DLB | NEWCASTLE | x | v | x | v |
| 155 | CAT_A021 | DLB | NEWCASTLE | x | v | x | v |
| 156 | CAT_A022 | DLB | NEWCASTLE | x | v | x | v |
| 157 | CAT_A023 | DLB | NEWCASTLE | x | v | x | v |
| 158 | CAT_A024 | DLB | NEWCASTLE | x | v | x | v |
| 159 | DTI_A006 | DLB | NEWCASTLE | x | v | v | v |
| 160 | DTI_A010 | DLB | NEWCASTLE | x | v | v | v |
| 161 | DTI_A015 | DLB | NEWCASTLE | x | v | v | v |
| 162 | NACC005356 | DLB | NACC | x | v | v | x |
| 163 | NACC019553 | DLB | NACC | x | v | v | x |
| 164 | NACC020045 | DLB | NACC | x | v | v | v |
| 165 | NACC047046 | DLB | NACC | x | v | v | x |
| 166 | NACC050056 | DLB | NACC | x | v | v | v |
| 167 | NACC063408 | DLB | NACC | x | v | v | x |
| 168 | NACC117016 | DLB | NACC | x | v | v | v |
| 169 | NACC146825 | DLB | NACC | x | v | v | v |
| 170 | NACC149200 | DLB | NACC | x | v | v | x |
| 171 | NACC224250 | DLB | NACC | x | v | v | x |
| 172 | NACC235583 | DLB | NACC | x | v | v | v |
| 173 | NACC260252 | DLB | NACC | x | v | v | x |
| 174 | NACC264704 | DLB | NACC | x | v | v | x |
| 175 | NACC291602 | DLB | NACC | x | v | v | v |
| 176 | NACC293198 | DLB | NACC | x | v | v | x |
| 177 | NACC348810 | DLB | NACC | x | v | v | v |
| 178 | NACC412399 | DLB | NACC | x | v | v | x |
| 179 | NACC441212 | DLB | NACC | x | v | v | x |
| 180 | NACC457926 | DLB | NACC | x | v | v | v |
| 181 | NACC483045 | DLB | NACC | x | v | v | v |
| 182 | NACC634713 | DLB | NACC | x | v | v | x |
| 183 | NACC639691 | DLB | NACC | x | v | v | v |
| 184 | NACC687319 | DLB | NACC | x | v | v | v |
| 185 | NACC784096 | DLB | NACC | x | v | v | x |
| 186 | NACC810369 | DLB | NACC | x | v | v | x |
| 187 | NACC878266 | DLB | NACC | x | v | v | x |
| 188 | NACC987145 | DLB | NACC | x | v | v | x |
| 189 | 005_S_0448 | DLB | ADNI | x | v | x | x |
| 190 | 011_S_0053 | DLB | ADNI | x | v | x | x |
| 191 | 011_S_0183 | DLB | ADNI | v | v | x | x |
| 192 | 011_S_1080 | DLB | ADNI | x | v | x | x |
| 193 | 014_S_4263 | DLB | ADNI | x | v | v | x |
| 194 | 027_S_4802 | DLB | ADNI | x | v | v | v |
| 195 | 027_S_4936 | DLB | ADNI | x | v | v | v |
| 196 | 029_S_1384 | DLB | ADNI | x | v | x | x |
| 197 | 031_S_0830 | DLB | ADNI | x | v | x | x |
| 198 | 033_S_0567 | DLB | ADNI | x | v | x | x |
| 199 | 033_S_0723 | DLB | ADNI | x | v | x | x |
| 200 | 033_S_0724 | DLB | ADNI | v | v | x | x |
| 201 | 033_S_1116 | DLB | ADNI | x | v | x | x |
| 202 | 116_S_0834 | DLB | ADNI | x | v | x | x |
| 203 | 123_S_4526 | DLB | ADNI | x | v | v | x |
| 204 | PDAM173HGK | DLB | PDBP | x | v | v | v |
| 205 | PDDJ916LE2 | DLB | PDBP | v | v | v | v |
| 206 | PDRM964PWK | DLB | PDBP | x | v | v | v |
| 207 | PDVU751PW2 | DLB | PDBP | x | v | v | v |
| 208 | PDZZ702FMD | DLB | PDBP | x | v | v | v |
| 209 | PDDD367DR5 | DLB | PDBP | x | v | v | v |
| 210 | NIHAL261NEKWD | DLB | PDBP | x | v | v | v |
| 211 | NIHAU490KYUNN | DLB | PDBP | x | v | v | v |
| 212 | NIHEB037KLBDN | DLB | PDBP | x | v | v | v |
| 213 | NIHFP876TRNX1 | DLB | PDBP | x | v | v | v |
| 214 | NIHHM473EPHXU | DLB | PDBP | x | v | v | v |
| 215 | NIHHV433UJPHN | DLB | PDBP | x | v | v | v |
| 216 | NIHLJ543TLJWK | DLB | PDBP | x | v | v | v |
| 217 | NIHMU318MGLEY | DLB | PDBP | x | v | v | v |
| 218 | NIHPC082CDKEE | DLB | PDBP | x | v | v | v |
| 219 | NIHPH466WFZVR | DLB | PDBP | x | v | v | v |
| 220 | NIHPU224VMVZA | DLB | PDBP | x | v | v | v |
| 221 | NIHRJ154HDCT3 | DLB | PDBP | x | v | v | v |
| 222 | NIHTG164CWET6 | DLB | PDBP | x | v | v | v |
| 223 | NIHVF281ZCJF9 | DLB | PDBP | x | v | v | v |
| 224 | NIHXK394KZHD2 | DLB | PDBP | x | v | v | v |
| 225 | NIHXR083TBDY8 | DLB | PDBP | x | v | v | v |
| 226 | NIHXV129BGYAV | DLB | PDBP | x | v | v | v |
| 227 | NIHZC615LXTZM | DLB | PDBP | x | v | v | v |
| 228 | PDAZ833LFN | DLB | PDBP | x | v | v | v |
| 229 | PDCE445RPY | DLB | PDBP | x | v | v | v |
| 230 | PDCK041ZHA | DLB | PDBP | x | v | v | v |
| 231 | PDCN994PTV | DLB | PDBP | x | v | v | v |
| 232 | PDDX687RNV | DLB | PDBP | x | v | v | v |
| 233 | PDDY486ZZA | DLB | PDBP | x | v | v | v |
| 234 | PDDY588JG0 | DLB | PDBP | x | v | v | v |
| 235 | PDDY775DHP | DLB | PDBP | x | v | v | v |
| 236 | PDEH664VJ6 | DLB | PDBP | x | v | v | v |
| 237 | PDEM319VBZ | DLB | PDBP | x | v | v | v |
| 238 | PDEU208JJP | DLB | PDBP | x | v | v | v |
| 239 | PDEY578FCR | DLB | PDBP | x | v | v | v |
| 240 | PDEZ829YJX | DLB | PDBP | v | v | v | v |
| 241 | PDGL350FGU | DLB | PDBP | x | v | v | v |
| 242 | PDGW392UY8 | DLB | PDBP | x | v | v | v |
| 243 | PDHW455ZF0 | DLB | PDBP | x | v | v | v |
| 244 | PDJK824NBP | DLB | PDBP | x | v | v | v |
| 245 | PDKC411TKR | DLB | PDBP | x | v | v | v |
| 246 | PDLR120PF1 | DLB | PDBP | x | v | v | v |
| 247 | PDMZ635MRP | DLB | PDBP | x | v | v | v |
| 248 | PDNC588RWP | DLB | PDBP | x | v | v | v |
| 249 | PDNU804GKF | DLB | PDBP | x | v | v | v |
| 250 | PDPJ294NPW | DLB | PDBP | x | v | v | v |
| 251 | PDRM021LWE | DLB | PDBP | x | v | v | v |
| 252 | PDTC768GBQ | DLB | PDBP | x | v | v | v |
| 253 | PDTK237WW8 | DLB | PDBP | x | v | v | v |
| 254 | PDTY056EU4 | DLB | PDBP | x | v | v | v |
| 255 | PDVB760TWQ | DLB | PDBP | x | v | v | v |
| 256 | PDVC905DF3 | DLB | PDBP | x | v | v | v |
| 257 | PDXP775GEZ | DLB | PDBP | x | v | v | v |
| 258 | PDYT202XBZ | DLB | PDBP | x | v | v | v |
| 259 | PDYW037TUX | DLB | PDBP | x | v | v | v |
| 260 | PDYX998KRJ | DLB | PDBP | x | v | v | v |
| 261 | NIHUY841YVPJP | DLB | PDBP | x | v | v | v |
| 262 | PDKT040VKT | DLB | PDBP | x | v | v | v |
| 263 | PDAP540UU5 | DLB | PDBP | x | v | v | v |
| 264 | 1_S_0002 | FTD | FTLDNI | x | v | v | v |
| 265 | 1_S_0003 | FTD | FTLDNI | x | v | v | v |
| 266 | 1_S_0005 | FTD | FTLDNI | x | v | v | v |
| 267 | 1_S_0006 | FTD | FTLDNI | x | v | v | v |
| 268 | 1_S_0007 | FTD | FTLDNI | x | v | v | v |
| 269 | 1_S_0009 | FTD | FTLDNI | x | v | v | v |
| 270 | 1_S_0010 | FTD | FTLDNI | x | v | v | v |
| 271 | 1_S_0011 | FTD | FTLDNI | x | v | v | v |
| 272 | 1_S_0012 | FTD | FTLDNI | x | v | v | v |
| 273 | 1_S_0013 | FTD | FTLDNI | x | v | v | v |
| 274 | 1_S_0015 | FTD | FTLDNI | x | v | v | v |
| 275 | 1_S_0016 | FTD | FTLDNI | x | v | v | v |
| 276 | 1_S_0020 | FTD | FTLDNI | x | v | v | v |
| 277 | 1_S_0022 | FTD | FTLDNI | x | v | v | v |
| 278 | 1_S_0026 | FTD | FTLDNI | x | v | v | v |
| 279 | 1_S_0029 | FTD | FTLDNI | x | v | v | v |
| 280 | 1_S_0031 | FTD | FTLDNI | x | v | v | v |
| 281 | 1_S_0032 | FTD | FTLDNI | x | v | v | v |
| 282 | 1_S_0035 | FTD | FTLDNI | x | v | v | v |
| 283 | 1_S_0036 | FTD | FTLDNI | x | v | v | v |
| 284 | 1_S_0038 | FTD | FTLDNI | x | v | v | v |
| 285 | 1_S_0039 | FTD | FTLDNI | x | v | v | v |
| 286 | 1_S_0041 | FTD | FTLDNI | x | v | v | v |
| 287 | 1_S_0042 | FTD | FTLDNI | x | v | v | v |
| 288 | 1_S_0043 | FTD | FTLDNI | x | v | v | v |
| 289 | 1_S_0050 | FTD | FTLDNI | x | v | v | v |
| 290 | 1_S_0051 | FTD | FTLDNI | x | v | v | v |
| 291 | 1_S_0053 | FTD | FTLDNI | x | v | v | v |
| 292 | 1_S_0056 | FTD | FTLDNI | x | v | v | v |
| 293 | 1_S_0057 | FTD | FTLDNI | x | v | v | v |
| 294 | 1_S_0058 | FTD | FTLDNI | x | v | v | v |
| 295 | 1_S_0059 | FTD | FTLDNI | x | v | v | v |
| 296 | 1_S_0061 | FTD | FTLDNI | x | v | v | v |
| 297 | 1_S_0062 | FTD | FTLDNI | x | v | v | v |
| 298 | 1_S_0064 | FTD | FTLDNI | x | v | v | v |
| 299 | 1_S_0065 | FTD | FTLDNI | x | v | v | v |
| 300 | 1_S_0067 | FTD | FTLDNI | x | v | v | v |
| 301 | 1_S_0069 | FTD | FTLDNI | x | v | v | v |
| 302 | 1_S_0070 | FTD | FTLDNI | x | v | v | v |
| 303 | 1_S_0072 | FTD | FTLDNI | x | v | v | v |
| 304 | 1_S_0073 | FTD | FTLDNI | x | v | v | v |
| 305 | 1_S_0079 | FTD | FTLDNI | x | v | v | v |
| 306 | 1_S_0081 | FTD | FTLDNI | x | v | v | v |
| 307 | 1_S_0082 | FTD | FTLDNI | x | v | v | v |
| 308 | 1_S_0084 | FTD | FTLDNI | x | v | v | v |
| 309 | 1_S_0085 | FTD | FTLDNI | x | v | v | v |
| 310 | 1_S_0086 | FTD | FTLDNI | x | v | v | v |
| 311 | 1_S_0089 | FTD | FTLDNI | x | v | v | v |
| 312 | 1_S_0091 | FTD | FTLDNI | x | v | v | v |
| 313 | 1_S_0092 | FTD | FTLDNI | x | v | v | v |
| 314 | 1_S_0094 | FTD | FTLDNI | x | v | v | v |
| 315 | 1_S_0096 | FTD | FTLDNI | x | v | v | v |
| 316 | 1_S_0097 | FTD | FTLDNI | x | v | v | v |
| 317 | 1_S_0098 | FTD | FTLDNI | x | v | v | v |
| 318 | 1_S_0099 | FTD | FTLDNI | x | v | v | v |
| 319 | 1_S_0100 | FTD | FTLDNI | x | v | v | v |
| 320 | 1_S_0102 | FTD | FTLDNI | x | v | v | v |
| 321 | 1_S_0105 | FTD | FTLDNI | x | v | v | v |
| 322 | 1_S_0106 | FTD | FTLDNI | x | v | v | v |
| 323 | 1_S_0109 | FTD | FTLDNI | x | v | v | v |
| 324 | 1_S_0110 | FTD | FTLDNI | x | v | v | v |
| 325 | 1_S_0112 | FTD | FTLDNI | x | v | v | v |
| 326 | 1_S_0119 | FTD | FTLDNI | x | v | v | v |
| 327 | 1_S_0123 | FTD | FTLDNI | x | v | v | v |
| 328 | 1_S_0124 | FTD | FTLDNI | x | v | v | v |
| 329 | 1_S_0125 | FTD | FTLDNI | x | v | v | v |
| 330 | 1_S_0127 | FTD | FTLDNI | x | v | v | v |
| 331 | 1_S_0128 | FTD | FTLDNI | x | v | v | v |
| 332 | 1_S_0129 | FTD | FTLDNI | x | v | v | v |
| 333 | 1_S_0130 | FTD | FTLDNI | x | v | v | v |
| 334 | 1_S_0139 | FTD | FTLDNI | x | v | v | v |
| 335 | 1_S_0140 | FTD | FTLDNI | x | v | v | v |
| 336 | 1_S_0141 | FTD | FTLDNI | x | v | v | v |
| 337 | 1_S_0145 | FTD | FTLDNI | x | v | v | v |
| 338 | 1_S_0166 | FTD | FTLDNI | x | v | v | v |
| 339 | 1_S_0169 | FTD | FTLDNI | x | v | v | v |
| 340 | 1_S_0172 | FTD | FTLDNI | x | v | v | v |
| 341 | 1_S_0187 | FTD | FTLDNI | x | v | v | v |
| 342 | 1_S_0188 | FTD | FTLDNI | x | v | v | v |
| 343 | 1_S_0192 | FTD | FTLDNI | x | v | v | v |
| 344 | 1_S_0194 | FTD | FTLDNI | x | v | v | v |
| 345 | 1_S_0195 | FTD | FTLDNI | x | v | v | v |
| 346 | 1_S_0199 | FTD | FTLDNI | x | v | v | v |
| 347 | 1_S_0203 | FTD | FTLDNI | x | v | v | v |
| 348 | 1_S_0204 | FTD | FTLDNI | x | v | v | v |
| 349 | 1_S_0206 | FTD | FTLDNI | x | v | v | v |
| 350 | 1_S_0207 | FTD | FTLDNI | x | v | v | v |
| 351 | 1_S_0208 | FTD | FTLDNI | x | v | v | v |
| 352 | 1_S_0209 | FTD | FTLDNI | x | v | v | v |
| 353 | 1_S_0216 | FTD | FTLDNI | x | v | v | v |
| 354 | 1_S_0219 | FTD | FTLDNI | x | v | v | v |
| 355 | 1_S_0225 | FTD | FTLDNI | x | v | v | v |
| 356 | 1_S_0230 | FTD | FTLDNI | x | v | v | v |
| 357 | 1_S_0318 | FTD | FTLDNI | x | v | v | v |
| 358 | 1_S_0320 | FTD | FTLDNI | x | v | v | v |
| 359 | 1_S_0323 | FTD | FTLDNI | x | v | v | v |
| 360 | 1_S_0324 | FTD | FTLDNI | x | v | v | v |
| 361 | 1_S_0328 | FTD | FTLDNI | x | v | v | v |
| 362 | 1_S_0330 | FTD | FTLDNI | x | v | v | v |
| 363 | 1_S_0331 | FTD | FTLDNI | x | v | v | v |
| 364 | 1_S_0332 | FTD | FTLDNI | x | v | v | v |
| 365 | 1_S_0333 | FTD | FTLDNI | x | v | v | v |
| 366 | 1_S_0334 | FTD | FTLDNI | x | v | v | v |
| 367 | 1_S_0338 | FTD | FTLDNI | x | v | v | v |
| 368 | 1_S_0340 | FTD | FTLDNI | x | v | v | v |
| 369 | 1_S_0341 | FTD | FTLDNI | x | v | v | v |
| 370 | 1_S_0342 | FTD | FTLDNI | x | v | v | v |
| 371 | 1_S_0344 | FTD | FTLDNI | x | v | v | v |
| 372 | 1_S_0345 | FTD | FTLDNI | x | v | v | v |
| 373 | 1_S_0346 | FTD | FTLDNI | x | v | v | v |
| 374 | 1_S_0347 | FTD | FTLDNI | x | v | v | v |
| 375 | 2_S_0001 | FTD | FTLDNI | x | v | v | v |
| 376 | 2_S_0002 | FTD | FTLDNI | x | v | v | v |
| 377 | 2_S_0004 | FTD | FTLDNI | x | v | v | v |
| 378 | 2_S_0006 | FTD | FTLDNI | x | v | v | v |
| 379 | 2_S_0009 | FTD | FTLDNI | x | v | v | v |
| 380 | 2_S_0013 | FTD | FTLDNI | x | v | v | v |
| 381 | 2_S_0014 | FTD | FTLDNI | x | v | v | v |
| 382 | 2_S_0016 | FTD | FTLDNI | x | v | v | v |
| 383 | 2_S_0021 | FTD | FTLDNI | x | v | v | v |
| 384 | 2_S_0022 | FTD | FTLDNI | x | v | v | v |
| 385 | 2_S_0027 | FTD | FTLDNI | x | v | v | v |
| 386 | 2_S_0029 | FTD | FTLDNI | x | v | v | v |
| 387 | 2_S_0030 | FTD | FTLDNI | x | v | v | v |
| 388 | 2_S_0031 | FTD | FTLDNI | x | v | v | v |
| 389 | 2_S_0032 | FTD | FTLDNI | x | v | v | v |
| 390 | 3_S_0001 | FTD | FTLDNI | x | v | v | v |
| 391 | 3_S_0003 | FTD | FTLDNI | x | v | v | v |
| 392 | 3_S_0004 | FTD | FTLDNI | x | v | v | v |
| 393 | 3_S_0006 | FTD | FTLDNI | x | v | v | v |
| 394 | 3_S_0007 | FTD | FTLDNI | x | v | v | v |
| 395 | 3_S_0008 | FTD | FTLDNI | x | v | v | v |
| 396 | 3_S_0010 | FTD | FTLDNI | x | v | v | v |
| 397 | 3_S_0011 | FTD | FTLDNI | x | v | v | v |
| 398 | 3_S_0012 | FTD | FTLDNI | x | v | v | v |
| 399 | 002_S_0413 | CN | ADNI | x | v | v | v |
| 400 | 002_S_6007 | CN | ADNI | x | v | v | v |
| 401 | 002_S_6103 | CN | ADNI | x | v | v | v |
| 402 | 003_S_4081 | CN | ADNI | x | v | v | v |
| 403 | 003_S_4119 | CN | ADNI | x | v | v | v |
| 404 | 003_S_4288 | CN | ADNI | x | v | v | v |
| 405 | 003_S_4350 | CN | ADNI | x | v | v | v |
| 406 | 003_S_4441 | CN | ADNI | x | v | v | v |
| 407 | 003_S_4555 | CN | ADNI | x | v | v | v |
| 408 | 003_S_4644 | CN | ADNI | x | v | v | v |
| 409 | 003_S_4872 | CN | ADNI | x | v | v | v |
| 410 | 003_S_4900 | CN | ADNI | x | v | v | v |
| 411 | 005_S_0610 | CN | ADNI | x | v | v | v |
| 412 | 007_S_4387 | CN | ADNI | x | v | v | v |
| 413 | 007_S_4488 | CN | ADNI | x | v | v | v |
| 414 | 007_S_4516 | CN | ADNI | x | v | v | v |
| 415 | 007_S_4620 | CN | ADNI | x | v | v | v |
| 416 | 007_S_4637 | CN | ADNI | x | v | v | v |
| 417 | 016_S_4097 | CN | ADNI | x | v | v | v |
| 418 | 016_S_4121 | CN | ADNI | x | v | v | v |
| 419 | 016_S_4638 | CN | ADNI | x | v | v | v |
| 420 | 016_S_4688 | CN | ADNI | x | v | v | v |
| 421 | 016_S_4951 | CN | ADNI | x | v | v | v |
| 422 | 016_S_4952 | CN | ADNI | x | v | v | v |
| 423 | 018_S_4400 | CN | ADNI | x | v | v | v |
| 424 | 019_S_6186 | CN | ADNI | x | v | v | v |
| 425 | 020_S_6185 | CN | ADNI | x | v | v | v |
| 426 | 021_S_4254 | CN | ADNI | x | v | v | v |
| 427 | 021_S_4276 | CN | ADNI | x | v | v | v |
| 428 | 021_S_4335 | CN | ADNI | x | v | v | v |
| 429 | 021_S_4421 | CN | ADNI | x | v | v | v |
| 430 | 021_S_4558 | CN | ADNI | x | v | v | v |
| 431 | 023_S_0031 | CN | ADNI | x | v | v | v |
| 432 | 023_S_4448 | CN | ADNI | x | v | v | v |
| 433 | 024_S_6005 | CN | ADNI | x | v | v | v |
| 434 | 029_S_4279 | CN | ADNI | x | v | v | v |
| 435 | 029_S_4290 | CN | ADNI | x | v | v | v |
| 436 | 029_S_4384 | CN | ADNI | x | v | v | v |
| 437 | 029_S_4385 | CN | ADNI | x | v | v | v |
| 438 | 029_S_4585 | CN | ADNI | x | v | v | v |
| 439 | 029_S_4652 | CN | ADNI | x | v | v | v |
| 440 | 032_S_6279 | CN | ADNI | x | v | v | v |
| 441 | 033_S_4176 | CN | ADNI | x | v | v | v |
| 442 | 033_S_4179 | CN | ADNI | x | v | v | v |
| 443 | 036_S_4491 | CN | ADNI | x | v | v | v |
| 444 | 037_S_0303 | CN | ADNI | x | v | v | v |
| 445 | 037_S_0454 | CN | ADNI | x | v | v | v |
| 446 | 037_S_4028 | CN | ADNI | x | v | v | v |
| 447 | 037_S_4071 | CN | ADNI | x | v | v | v |
| 448 | 057_S_0934 | CN | ADNI | x | v | v | v |
| 449 | 094_S_4234 | CN | ADNI | x | v | v | v |
| 450 | 094_S_4503 | CN | ADNI | x | v | v | v |
| 451 | 094_S_4560 | CN | ADNI | x | v | v | v |
| 452 | 094_S_4649 | CN | ADNI | x | v | v | v |
| 453 | 098_S_0896 | CN | ADNI | x | v | v | v |
| 454 | 098_S_4003 | CN | ADNI | x | v | v | v |
| 455 | 098_S_4018 | CN | ADNI | x | v | v | v |
| 456 | 098_S_4275 | CN | ADNI | x | v | v | v |
| 457 | 098_S_4506 | CN | ADNI | x | v | v | v |
| 458 | 099_S_4076 | CN | ADNI | x | v | v | v |
| 459 | 099_S_4086 | CN | ADNI | x | v | v | v |
| 460 | 099_S_4104 | CN | ADNI | x | v | v | v |
| 461 | 109_S_4499 | CN | ADNI | x | v | v | v |
| 462 | 116_S_4043 | CN | ADNI | x | v | v | v |
| 463 | 116_S_4855 | CN | ADNI | x | v | v | v |
| 464 | 126_S_0680 | CN | ADNI | x | v | v | v |
| 465 | 127_S_4148 | CN | ADNI | x | v | v | v |
| 466 | 127_S_4198 | CN | ADNI | x | v | v | v |
| 467 | 127_S_4604 | CN | ADNI | x | v | v | v |
| 468 | 127_S_4645 | CN | ADNI | x | v | v | v |
| 469 | 127_S_4843 | CN | ADNI | x | v | v | v |
| 470 | 129_S_0778 | CN | ADNI | x | v | v | v |
| 471 | 129_S_4369 | CN | ADNI | x | v | v | v |
| 472 | 129_S_4371 | CN | ADNI | x | v | v | v |
| 473 | 129_S_4396 | CN | ADNI | x | v | v | v |
| 474 | 129_S_4422 | CN | ADNI | x | v | v | v |
| 475 | 130_S_4352 | CN | ADNI | x | v | v | v |
| 476 | 130_S_6319 | CN | ADNI | x | v | v | v |
| 477 | 131_S_0123 | CN | ADNI | x | v | v | v |
| 478 | 141_S_6178 | CN | ADNI | x | v | v | v |
| 479 | 1_S_0121 | CN | FTLDNI | x | v | v | v |
| 480 | 1_S_0153 | CN | FTLDNI | x | v | v | v |
| 481 | 1_S_0163 | CN | FTLDNI | x | v | v | v |
| 482 | 1_S_0164 | CN | FTLDNI | x | v | v | v |
| 483 | 1_S_0178 | CN | FTLDNI | x | v | v | v |
| 484 | 1_S_0184 | CN | FTLDNI | x | v | v | v |
| 485 | 1_S_0198 | CN | FTLDNI | x | v | v | v |
| 486 | 1_S_0255 | CN | FTLDNI | x | v | v | v |
| 487 | 1_S_0259 | CN | FTLDNI | x | v | v | v |
| 488 | 1_S_0301 | CN | FTLDNI | x | v | v | v |
| 489 | NACC003112 | CN | NACC | x | v | v | v |
| 490 | NACC013678 | CN | NACC | x | v | v | v |
| 491 | NACC016407 | CN | NACC | x | v | v | v |
| 492 | NACC077197 | CN | NACC | x | v | v | v |
| 493 | NACC099528 | CN | NACC | x | v | v | v |
| 494 | NACC176565 | CN | NACC | x | v | v | v |
| 495 | NACC249885 | CN | NACC | x | v | v | v |
| 496 | NACC255888 | CN | NACC | x | v | v | v |
| 497 | NACC329492 | CN | NACC | x | v | v | v |
| 498 | NACC389676 | CN | NACC | x | v | v | v |
| 499 | NACC463343 | CN | NACC | x | v | v | v |
| 500 | NACC501851 | CN | NACC | x | v | v | v |
| 501 | NACC600069 | CN | NACC | x | v | v | v |
| 502 | NACC686972 | CN | NACC | x | v | v | v |
| 503 | NACC739901 | CN | NACC | x | v | v | v |
| 504 | NACC808407 | CN | NACC | x | v | v | v |
| 505 | NACC848567 | CN | NACC | x | v | v | v |
| 506 | NACC923186 | CN | NACC | x | v | v | v |

*The table reports all the subjects included in the study, the diagnosis, the database of belonging, the availability of the neuropathologic data and the acquired sequences. Acronyms: AD, Alzheimer’s dementia; DLB, dementia with Lewy bodies; FTD, frontotemporal dementia; CN, cognitively normal controls; v, available; x, not available*

**Supplementary Table S3:** Quantification of variance in Fractional Anisotropy and Mean Diffusivity based on diagnosis, ventricular volume, and white matter hyperintensity in tracts considered by MUQUBIA

| **DTI Metrics** | | **Δ AIC** **(Model1 – Model2)** | **DX** | | **Ventricle [mm3]** | | **WMH [mm3]** | |
| --- | --- | --- | --- | --- | --- | --- | --- | --- |
|  |  |  | **Variance %** | **P-value** | **Variance %** | **P-value** | **Variance %** | **P-value** |
| **FA** | **LH Corticospinal tract** | -1.905 | 21.5 | <0.001 | 3.67 | <0.001 | N.A. | N.A. |
|  | **LH Superior fronto occipital fasciculus** | 2.454 | 17.49 | <0.001 | 27.6 | <0.001 | 0.89 | <0.05† |
|  | **Splenium of corpus callosum** | -0.995 | 18.79 | <0.001 | 23.55 | <0.001 | N.A. | N.A. |
| **MD** | **RH Medial lemniscus** | 0.724 | 28.98 | <0.001 | 1.33 | <0.001 | 0.69 | N.S. |
|  | **RH Retrolenticular part of internal capsule** | 0.81 | 11.54 | <0.001 | 20.17 | <0.001 | 0.7 | N.S. |
|  | **Pontine crossing tract** | -1.989 | 7.79 | <0.001 | 4.78 | <0.001 | N.A. | N.A. |
|  | **RH Cerebral peduncle** | -0.814 | 6.57 | <0.001 | 7.31 | <0.001 | N.A. | N.A. |

*Two multivariate linear regression models were designed to test whether and to what extent White Matter Hiperintensity (WMH) load affects DTI FA and MD estimates. In Model 1, the dependent variable was the DTI estimate for each selected tract whereas the independent variables were diagnosis and ventricle volume [9]. In Model 2, WMH volume was added as an additional independent variable. The present table shows that Model2 (with WMH effect) is a better model (Δ AIC > 0) to explain the variance of DTI metrics in the three tracts (i.e.: LH superior fronto occipital fasciculus, RH medial lemniscus, RH retrolenticular part of internal capsule) used by MUQUBIA. As for FA, the* *superior fronto occipital fasciculus tract is slightly influenced by WMH. Conversely, MD is not statistically influenced by the WMH effect. Variance % indicates the contribution of each independent variable to the total variance of the distribution. All predictors accounted for a maximum total variance of 45.98% for FA, and a maximum total variance of 32.41% for MD. Acronyms: N.S., Not Significant; N.A., Not applicable; AIC, Akaike’s Information Criteria; LH, Left Hemisphere; RH, Right Hemisphere; FA, Fractional Anisotropy; MD, Mean Diffusivity; †, Cohen’s d < 0.2 (i.e.: small effect size).*

**Supplementary Table S4:** List of all the 340 collected features.

| N° | **Feature’s name** | **Pipeline** |
| --- | --- | --- |
| 1 | Left-Lateral-Ventricle_Vol | FS |
| 2 | Left-Inf-Lat-Vent_Vol | FS |
| 3 | Left-Cerebellum-White-Matter_Vol | FS |
| 4 | Left-Cerebellum-Cortex_Vol | FS |
| 5 | Left-Thalamus-Proper_Vol | FS |
| 6 | Left-Caudate_Vol | FS |
| 7 | Left-Putamen_Vol  (LH putamen volume) | FS |
| 8 | Left-Pallidum_Vol  (LH pallidum volume) | FS |
| 9 | 3rd-Ventricle_Vol | FS |
| 10 | 4th-Ventricle_Vol | FS |
| 11 | Brain-Stem_Vol | FS |
| 12 | Left-Hippocampus_Vol  (LH hippocampus volume) | FS |
| 13 | Left-Amygdala_Vol | FS |
| 14 | CSF_Vol | FS |
| 15 | Left-Accumbens_Vol | FS |
| 16 | Left-VentralDC_Vol | FS |
| 17 | Left-vessel_Vol | FS |
| 18 | Left-choroid-plexus_Vol | FS |
| 19 | Right-Lateral-Ventricle_Vol | FS |
| 20 | Right-Inf-Lat-Vent_Vol | FS |
| 21 | Right-Cerebellum-White-Matter_Vol | FS |
| 22 | Right-Cerebellum-Cortex_Vol | FS |
| 23 | Right-Thalamus-Proper_Vol | FS |
| 24 | Right-Caudate_Vol | FS |
| 25 | Right-Putamen_Vol | FS |
| 26 | Right-Pallidum_Vol | FS |
| 27 | Right-Hippocampus_Vol | FS |
| 28 | Right-Amygdala_Vol | FS |
| 29 | Right-Accumbens_Vol | FS |
| 30 | Right-VentralDC_Vol | FS |
| 31 | Right-vessel_Vol | FS |
| 32 | Right-choroid-plexus_Vol | FS |
| 33 | 5th-Ventricle_Vol | FS |
| 34 | WM-hypointensities_Vol | FS |
| 35 | Left-WM-hypointensities_Vol | FS |
| 36 | Right-WM-hypointensities_Vol | FS |
| 37 | non-WM-hypointensities_Vol | FS |
| 38 | Left-non-WM-hypointensities_Vol | FS |
| 39 | Right-non-WM-hypointensities_Vol | FS |
| 40 | Optic-Chiasm_Vol | FS |
| 41 | CC_Posterior_Vol | FS |
| 42 | CC_Mid_Posterior_Vol | FS |
| 43 | CC_Central_Vol | FS |
| 44 | CC_Mid_Anterior_Vol | FS |
| 45 | CC_Anterior_Vol | FS |
| 46 | BrainSegVol | FS |
| 47 | BrainSegVolNotVent | FS |
| 48 | BrainSegVolNotVentSurf | FS |
| 49 | lhCortexVol  (LH cortex volume) | FS |
| 50 | rhCortexVol | FS |
| 51 | CortexVol | FS |
| 52 | lhCerebralWhiteMatterVol | FS |
| 53 | rhCerebralWhiteMatterVol | FS |
| 54 | CerebralWhiteMatterVol | FS |
| 55 | SubCortGrayVol | FS |
| 56 | TotalGrayVol | FS |
| 57 | SupraTentorialVol | FS |
| 58 | SupraTentorialVolNotVent | FS |
| 59 | SupraTentorialVolNotVentVox | FS |
| 60 | MaskVol | FS |
| 61 | lhSurfaceHoles | FS |
| 62 | rhSurfaceHoles | FS |
| 63 | SurfaceHoles | FS |
| 64 | EstimatedTotalIntraCranialVol | FS |
| 65 | lh_bankssts_volume | FS |
| 66 | lh_caudalanteriorcingulate_volume | FS |
| 67 | lh_caudalmiddlefrontal_volume | FS |
| 68 | lh_cuneus_volume | FS |
| 69 | lh_entorhinal_volume | FS |
| 70 | lh_fusiform_volume | FS |
| 71 | lh_inferiorparietal_volume | FS |
| 72 | lh_inferiortemporal_volume | FS |
| 73 | lh_isthmuscingulate_volume | FS |
| 74 | lh_lateraloccipital_volume | FS |
| 75 | lh_lateralorbitofrontal_volume  (LH lateral orbitofrontal volume) | FS |
| 76 | lh_lingual_volume | FS |
| 77 | lh_medialorbitofrontal_volume | FS |
| 78 | lh_middletemporal_volume | FS |
| 79 | lh_parahippocampal_volume | FS |
| 80 | lh_paracentral_volume | FS |
| 81 | lh_parsopercularis_volume | FS |
| 82 | lh_parsorbitalis_volume | FS |
| 83 | lh_parstriangularis_volume | FS |
| 84 | lh_pericalcarine_volume | FS |
| 85 | lh_postcentral_volume | FS |
| 86 | lh_posteriorcingulate_volume | FS |
| 87 | lh_precentral_volume | FS |
| 88 | lh_precuneus_volume | FS |
| 89 | lh_rostralanteriorcingulate_volume | FS |
| 90 | lh_rostralmiddlefrontal_volume | FS |
| 91 | lh_superiorfrontal_volume | FS |
| 92 | lh_superiorparietal_volume | FS |
| 93 | lh_superiortemporal_volume | FS |
| 94 | lh_supramarginal_volume | FS |
| 95 | lh_frontalpole_volume  (LH frontal pole volume) | FS |
| 96 | lh_temporalpole_volume | FS |
| 97 | lh_transversetemporal_volume | FS |
| 98 | lh_insula_volume | FS |
| 99 | rh_bankssts_volume | FS |
| 100 | rh_caudalanteriorcingulate_volume | FS |
| 101 | rh_caudalmiddlefrontal_volume | FS |
| 102 | rh_cuneus_volume | FS |
| 103 | rh_entorhinal_volume | FS |
| 104 | rh_fusiform_volume | FS |
| 105 | rh_inferiorparietal_volume | FS |
| 106 | rh_inferiortemporal_volume | FS |
| 107 | rh_isthmuscingulate_volume | FS |
| 108 | rh_lateraloccipital_volume | FS |
| 109 | rh_lateralorbitofrontal_volume | FS |
| 110 | rh_lingual_volume | FS |
| 111 | rh_medialorbitofrontal_volume | FS |
| 112 | rh_middletemporal_volume | FS |
| 113 | rh_parahippocampal_volume | FS |
| 114 | rh_paracentral_volume | FS |
| 115 | rh_parsopercularis_volume | FS |
| 116 | rh_parsorbitalis_volume | FS |
| 117 | rh_parstriangularis_volume | FS |
| 118 | rh_pericalcarine_volume | FS |
| 119 | rh_postcentral_volume | FS |
| 120 | rh_posteriorcingulate_volume | FS |
| 121 | rh_precentral_volume | FS |
| 122 | rh_precuneus_volume | FS |
| 123 | rh_rostralanteriorcingulate_volume | FS |
| 124 | rh_rostralmiddlefrontal_volume | FS |
| 125 | rh_superiorfrontal_volume | FS |
| 126 | rh_superiorparietal_volume | FS |
| 127 | rh_superiortemporal_volume | FS |
| 128 | rh_supramarginal_volume | FS |
| 129 | rh_frontalpole_volume | FS |
| 130 | rh_temporalpole_volume | FS |
| 131 | rh_transversetemporal_volume | FS |
| 132 | rh_insula_volume | FS |
| 133 | lh_bankssts_thickness | FS |
| 134 | lh_caudalanteriorcingulate_thickness | FS |
| 135 | lh_caudalmiddlefrontal_thickness | FS |
| 136 | lh_cuneus_thickness | FS |
| 137 | lh_entorhinal_thickness  (LH entorhinal thickness) | FS |
| 138 | lh_fusiform_thickness | FS |
| 139 | lh_inferiorparietal_thickness  (LH inferior parietal thickness) | FS |
| 140 | lh_inferiortemporal_thickness | FS |
| 141 | lh_isthmuscingulate_thickness | FS |
| 142 | lh_lateraloccipital_thickness | FS |
| 143 | lh_lateralorbitofrontal_thickness | FS |
| 144 | lh_lingual_thickness | FS |
| 145 | lh_medialorbitofrontal_thickness | FS |
| 146 | lh_middletemporal_thickness | FS |
| 147 | lh_parahippocampal_thickness | FS |
| 148 | lh_paracentral_thickness | FS |
| 149 | lh_parsopercularis_thickness  (LH pars opercularis thickness) | FS |
| 150 | lh_parsorbitalis_thickness | FS |
| 151 | lh_parstriangularis_thickness | FS |
| 152 | lh_pericalcarine_thickness | FS |
| 153 | lh_postcentral_thickness | FS |
| 154 | lh_posteriorcingulate_thickness  (LH posterior cingulate thickness) | FS |
| 155 | lh_precentral_thickness | FS |
| 156 | lh_precuneus_thickness | FS |
| 157 | lh_rostralanteriorcingulate_thickness | FS |
| 158 | lh_rostralmiddlefrontal_thickness | FS |
| 159 | lh_superiorfrontal_thickness | FS |
| 160 | lh_superiorparietal_thickness | FS |
| 161 | lh_superiortemporal_thickness | FS |
| 162 | lh_supramarginal_thickness | FS |
| 163 | lh_frontalpole_thickness | FS |
| 164 | lh_temporalpole_thickness  (LH temporal pole thickness) | FS |
| 165 | lh_transversetemporal_thickness | FS |
| 166 | lh_insula_thickness | FS |
| 167 | lh_MeanThickness_thickness | FS |
| 168 | rh_bankssts_thickness | FS |
| 169 | rh_caudalanteriorcingulate_thickness | FS |
| 170 | rh_caudalmiddlefrontal_thickness | FS |
| 171 | rh_cuneus_thickness | FS |
| 172 | rh_entorhinal_thickness | FS |
| 173 | rh_fusiform_thickness | FS |
| 174 | rh_inferiorparietal_thickness | FS |
| 175 | rh_inferiortemporal_thickness | FS |
| 176 | rh_isthmuscingulate_thickness  (RH isthmus cingulate thickness) | FS |
| 177 | rh_lateraloccipital_thickness | FS |
| 178 | rh_lateralorbitofrontal_thickness | FS |
| 179 | rh_lingual_thickness | FS |
| 180 | rh_medialorbitofrontal_thickness | FS |
| 181 | rh_middletemporal_thickness | FS |
| 182 | rh_parahippocampal_thickness | FS |
| 183 | rh_paracentral_thickness | FS |
| 184 | rh_parsopercularis_thickness | FS |
| 185 | rh_parsorbitalis_thickness | FS |
| 186 | rh_parstriangularis_thickness | FS |
| 187 | rh_pericalcarine_thickness | FS |
| 188 | rh_postcentral_thickness | FS |
| 189 | rh_posteriorcingulate_thickness | FS |
| 190 | rh_precentral_thickness | FS |
| 191 | rh_precuneus_thickness | FS |
| 192 | rh_rostralanteriorcingulate_thickness | FS |
| 193 | rh_rostralmiddlefrontal_thickness | FS |
| 194 | rh_superiorfrontal_thickness | FS |
| 195 | rh_superiorparietal_thickness | FS |
| 196 | rh_superiortemporal_thickness | FS |
| 197 | rh_supramarginal_thickness | FS |
| 198 | rh_frontalpole_thickness | FS |
| 199 | rh_temporalpole_thickness | FS |
| 200 | rh_transversetemporal_thickness | FS |
| 201 | rh_insula_thickness | FS |
| 202 | rh_MeanThickness_thickness | FS |
| 203 | LPA_lesions_vol_norm | LPA |
| 204 | LPA_lesions_number | LPA |
| 205 | MD_Avg_Center_fmajor_PP | TRACULA |
| 206 | FA_Avg_Center_fmajor_PP | TRACULA |
| 207 | MD_Avg_Center_fminor_PP | TRACULA |
| 208 | FA_Avg_Center_fminor_PP | TRACULA |
| 209 | MD_Avg_Center_lh.atr_PP | TRACULA |
| 210 | FA_Avg_Center_lh.atr_PP | TRACULA |
| 211 | MD_Avg_Center_lh.cab_PP | TRACULA |
| 212 | FA_Avg_Center_lh.cab_PP | TRACULA |
| 213 | MD_Avg_Center_lh.ccg_PP | TRACULA |
| 214 | FA_Avg_Center_lh.ccg_PP | TRACULA |
| 215 | MD_Avg_Center_lh.cst_AS | TRACULA |
| 216 | FA_Avg_Center_lh.cst_AS  (LH corticospinal tract FA) | TRACULA |
| 217 | MD_Avg_Center_lh.ilf_AS | TRACULA |
| 218 | FA_Avg_Center_lh.ilf_AS | TRACULA |
| 219 | MD_Avg_Center_lh.slfp_PP | TRACULA |
| 220 | FA_Avg_Center_lh.slfp_PP | TRACULA |
| 221 | MD_Avg_Center_lh.slft_PP | TRACULA |
| 222 | FA_Avg_Center_lh.slft_PP | TRACULA |
| 223 | MD_Avg_Center_lh.unc_AS | TRACULA |
| 224 | FA_Avg_Center_lh.unc_AS | TRACULA |
| 225 | MD_Avg_Center_rh.atr_PP | TRACULA |
| 226 | FA_Avg_Center_rh.atr_PP | TRACULA |
| 227 | MD_Avg_Center_rh.cab_PP | TRACULA |
| 228 | FA_Avg_Center_rh.cab_PP | TRACULA |
| 229 | MD_Avg_Center_rh.ccg_PP | TRACULA |
| 230 | FA_Avg_Center_rh.ccg_PP | TRACULA |
| 231 | MD_Avg_Center_rh.cst_AS | TRACULA |
| 232 | FA_Avg_Center_rh.cst_AS | TRACULA |
| 233 | MD_Avg_Center_rh.ilf_AS | TRACULA |
| 234 | FA_Avg_Center_rh.ilf_AS | TRACULA |
| 235 | MD_Avg_Center_rh.slfp_PP | TRACULA |
| 236 | FA_Avg_Center_rh.slfp_PP | TRACULA |
| 237 | MD_Avg_Center_rh.slft_PP | TRACULA |
| 238 | FA_Avg_Center_rh.slft_PP | TRACULA |
| 239 | MD_Avg_Center_rh.unc_AS | TRACULA |
| 240 | FA_Avg_Center_rh.unc_AS | TRACULA |
| 241 | Middle_cerebellar_peduncle_1_FA | JHU |
| 242 | Middle_cerebellar_peduncle_1_MD | JHU |
| 243 | Pontine_crossing_tract_2_FA | JHU |
| 244 | Pontine_crossing_tract_2_MD  (Pontine crossing tract MD) | JHU |
| 245 | Genu_of_corpus_callosum_3_FA | JHU |
| 246 | Genu_of_corpus_callosum_3_MD | JHU |
| 247 | Body_of_corpus_callosum_4_FA | JHU |
| 248 | Body_of_corpus_callosum_4_MD | JHU |
| 249 | Splenium_of_corpus_callosum_5_FA  (Splenium of corpus callosum FA) | JHU |
| 250 | Splenium_of_corpus_callosum_5_MD | JHU |
| 251 | Fornix_6_FA | JHU |
| 252 | Fornix_6_MD | JHU |
| 253 | Corticospinal_tract_R_7_FA | JHU |
| 254 | Corticospinal_tract_R_7_MD | JHU |
| 255 | Corticospinal_tract_L_8_FA | JHU |
| 256 | Corticospinal_tract_L_8_MD | JHU |
| 257 | Medial_lemniscus_R_9_FA | JHU |
| 258 | Medial_lemniscus_R_9_MD  (RH medial lemniscus MD) | JHU |
| 259 | Medial_lemniscus_L_10_FA | JHU |
| 260 | Medial_lemniscus_L_10_MD | JHU |
| 261 | Inferior_cerebellar_peduncle_R_11_FA | JHU |
| 262 | Inferior_cerebellar_peduncle_R_11_MD | JHU |
| 263 | Inferior_cerebellar_peduncle_L_12_FA | JHU |
| 264 | Inferior_cerebellar_peduncle_L_12_MD | JHU |
| 265 | Superior_cerebellar_peduncle_R_13_FA | JHU |
| 266 | Superior_cerebellar_peduncle_R_13_MD | JHU |
| 267 | Superior_cerebellar_peduncle_L_14_FA | JHU |
| 268 | Superior_cerebellar_peduncle_L_14_MD | JHU |
| 269 | Cerebral_peduncle_R_15_FA | JHU |
| 270 | Cerebral_peduncle_R_15_MD  (RH cerebral peduncle MD) | JHU |
| 271 | Cerebral_peduncle_L_16_FA | JHU |
| 272 | Cerebral_peduncle_L_16_MD | JHU |
| 273 | Anterior_limb_of_internal_capsule_R_17_FA | JHU |
| 274 | Anterior_limb_of_internal_capsule_R_17_MD | JHU |
| 275 | Anterior_limb_of_internal_capsule_L_18_FA | JHU |
| 276 | Anterior_limb_of_internal_capsule_L_18_MD | JHU |
| 277 | Posterior_limb_internal_capsule_R_19_FA | JHU |
| 278 | Posterior_limb_internal_capsule_R_19_MD | JHU |
| 279 | Posterior_limb_of_internal_capsule_L_20_FA | JHU |
| 280 | Posterior_limb_of_internal_capsule_L_20_MD | JHU |
| 281 | Retrolenticular_part_of_internal_capsule_R_21_FA | JHU |
| 282 | Retrolenticular_part_of_internal_capsule_R_21_MD  (RH retrolenticular part of internal capsule MD) | JHU |
| 283 | Retrolenticular_part_of_internal_capsule_L_22_FA | JHU |
| 284 | Retrolenticular_part_of_internal_capsule_L_22_MD | JHU |
| 285 | Anterior_corona_radiata_R_23_FA | JHU |
| 286 | Anterior_corona_radiata_R_23_MD | JHU |
| 287 | Anterior_corona_radiata_L_24_FA | JHU |
| 288 | Anterior_corona_radiata_L_24_MD | JHU |
| 289 | Superior_corona_radiata_R_25_FA | JHU |
| 290 | Superior_corona_radiata_R_25_MD | JHU |
| 291 | Superior_corona_radiata_L_26_FA | JHU |
| 292 | Superior_corona_radiata_L_26_MD | JHU |
| 293 | Posterior_corona_radiata_R_27_FA | JHU |
| 294 | Posterior_corona_radiata_R_27_MD | JHU |
| 295 | Posterior_corona_radiata_L_28_FA | JHU |
| 296 | Posterior_corona_radiata_L_28_MD | JHU |
| 297 | Posterior_thalamic_radiation_R_29_FA | JHU |
| 298 | Posterior_thalamic_radiation_R_29_MD | JHU |
| 299 | Posterior_thalamic_radiation_L_30_FA | JHU |
| 300 | Posterior_thalamic_radiation_L_30_MD | JHU |
| 301 | Sagittal_stratum_R_31_FA | JHU |
| 302 | Sagittal_stratum_R_31_MD | JHU |
| 303 | Sagittal_stratum_L_32_FA | JHU |
| 304 | Sagittal_stratum_L_32_MD | JHU |
| 305 | External_capsule_R_33_FA | JHU |
| 306 | External_capsule_R_33_MD | JHU |
| 307 | External_capsule_L_34_FA | JHU |
| 308 | External_capsule_L_34_MD | JHU |
| 309 | Cingulum_cingulate_gyrus_R_35_FA | JHU |
| 310 | Cingulum_cingulate_gyrus_R_35_MD | JHU |
| 311 | Cingulum_cingulate_gyrus_L_36_FA | JHU |
| 312 | Cingulum_cingulate_gyrus_L_36_MD | JHU |
| 313 | Cingulum_hippocampus_R_37_FA | JHU |
| 314 | Cingulum_hippocampus_R_37_MD | JHU |
| 315 | Cingulum_hippocampus_L_38_FA | JHU |
| 316 | Cingulum_hippocampus_L_38_MD | JHU |
| 317 | Fornix_cres_Stria_terminalis_R_39_FA | JHU |
| 318 | Fornix_cres_Stria_terminalis_R_39_MD | JHU |
| 319 | Fornix_cres_Stria_terminalis_L_40_FA | JHU |
| 320 | Fornix_cres_Stria_terminalis_L_40_MD | JHU |
| 321 | Superior_longitudinal_fasciculus_R_41_FA | JHU |
| 322 | Superior_longitudinal_fasciculus_R_41_MD | JHU |
| 323 | Superior_longitudinal_fasciculus_L_42_FA | JHU |
| 324 | Superior_longitudinal_fasciculus_L_42_MD | JHU |
| 325 | Superior_fronto_occipital_fasciculus_R_43_FA | JHU |
| 326 | Superior_fronto_occipital_fasciculus_R_43_MD | JHU |
| 327 | Superior_fronto_occipital_fasciculus_L_44_FA  (LH superior fronto-occipital fasciculus FA) | JHU |
| 328 | Superior_fronto_occipital_fasciculus_L_44_MD | JHU |
| 329 | Uncinate_fasciculus_R_45_FA | JHU |
| 330 | Uncinate_fasciculus_R_45_MD | JHU |
| 331 | Uncinate_fasciculus_L_46_FA | JHU |
| 332 | Uncinate_fasciculus_L_46_MD | JHU |
| 333 | Tapetum_R_47_FA | JHU |
| 334 | Tapetum_R_47_MD | JHU |
| 335 | Tapetum_L_48_FA | JHU |
| 336 | Tapetum_L_48_MD | JHU |
| 337 | AGE | // |
| 338 | GENDER | // |
| 339 | EDUCATION | // |
| 340 | CDR | // |

*The table reports all the features collected and the pipelines used to quantify them. The coding of the names of the informative features selected by the algorithm MUQUBIA is reported in the parentheses. Acronyms: CSF, cerebrospinal fluid; CC, corpus callosum; CDR, Clinical Dementia Rating; WM, white matter; lh or LH, left hemisphere; rh or RH, right hemisphere; eTIV, estimated total intracranial volume; LPA, lesion prediction algorithm; MD, mean diffusivity; FA, fractional anisotropy, atr: Anterior thalamic radiation, cab: Cingulum-Angular Bundle, ccg: Cingulum-Cingulate Gyrus, cst: corticospinal tract, ilf: inferior longitudinal fasciculus, slfp: superior longitudinal fasciculus- Parietal, slft: SLF-Temporal, unc: uncinate fasciculus, fmajor, forceps major; fminor, forceps minor.*

**Supplementary Table S5:** Influence of age and gender on the MRI features

| **MRI features** | **P-values without age and gender as covariates** | **P-values with age and gender as covariates** | **Comparison with and without correction** |
| --- | --- | --- | --- |
| LH corticospinal tract | <0.05§^*£ç | <0.05 §^*£ç | 0.73 &• |
| LH superior fronto-occipital fasciculus | <0.05§*£ | <0.05 §°*£ç | 0.47 @&• |
| RH medial lemniscus [mm^2^/s] | <0.05^°*£ç | <0.05 ^°*£ç | 0.69 & |
| LH entorhinal [mm] | <0.05§^*£ç | <0.05 § | 0.99 |
| LH hippocampus [mm^3^] | <0.05§*£ | <0.05 §^*£ç | 0.79• |
| LH inferior parietal [mm] | <0.05§^° | <0.05 §^° | 0.77• |
| LH cortex [mm^3^] | <0.05§*£ | <0.05 §*£ | 0.94• |
| LH putamen [mm^3^] | <0.05§*£ | <0.05 §°*£ç | 0.91 |
| LH frontal pole [mm^3^] | <0.05§^°ç | <0.05 § | 0.95 |
| RH retrolenticular part of internal capsule [mm^2^/s] | <0.05§^*£ç | <0.05 §^°£ç | 0.70 &• |
| LH pars opercularis [mm] | <0.05§^ | <0.05 §^£ç | 0.94 |
| Pontine crossing tract [mm^2^/s] | <0.05§^*£ç | <0.05 ^*£ç | 0.89• |
| Splenium of corpus callosum | <0.05§^°*£ç | <0.05 §^*£ç | 0.75 &• |
| LH pallidum [mm^3^] | <0.05*£ | <0.05 °£ç | 0.99 |
| RH isthmus cingulate [mm] | <0.05§* | <0.05 §*£ | 0.93 |
| LH lateral orbitofrontal [mm^3^] | <0.05§^°£ç | <0.05 §^°£ç | 0.73 |
| LH posterior cingulate [mm] | <0.05§°*ç | <0.05 §* | 0.95• |
| RH cerebral peduncle [mm^2^/s] | <0.05^°*£ | <0.05 ^°*£ | 0.84 &• |
| LH temporal pole [mm] | <0.05§^£ç | <0.05 §^°£ç | 0.94 |

*P-values were determined using the GLM with age and gender as covariates or Kruskal-Wallis test to investigate the influence of these two variables. Acronyms: MD, mean diffusivity; FA, fractional anisotropy; GLM Generalized Linear Model; lh, left hemisphere; rh, right hemisphere §, Post-hoc significant analysis difference between AD and CN; ^, Post-hoc significant analysis difference between AD and DLB; °, Post-hoc significant analysis difference between AD and FTD; *, Post-hoc significant analysis difference between CN and DLB; £, Post-hoc significant analysis difference between CN and FTD; ç, Post-hoc significant analysis difference between DLB and FTD; @ Significant difference between AD corrected for age and sex and not corrected; & Significant difference between DLB corrected and not corrected; • Significant difference between FTD corrected and not corrected.*

**Supplementary Table S6:** Comparison of the informative features between training and test set.

| **Feature** | **train set** | **test set** | **P-value** |
| --- | --- | --- | --- |
| CDR | 0.71±0.52 | 0.81±0.53 | 0.09 |
| LH corticospinal tract FA | 0.53±0.16 | 0.54±0.16 | 0.38 |
| LH superior fronto-occipital fasciculus FA | 0.42±0.22 | 0.39±0.21 | 0.25 |
| RH medial lemniscus MD [mm²/s] | 6.7⋅10^-4^±3.3⋅10^-4^ | 6.8⋅10^-4^±3.5⋅10^-4^ | 0.71 |
| LH entorhinal thickness [mm] | 2.82±0.62 | 2.71±0.66 | 0.09 |
| LH hippocampus volume [mm³] | 3087±540 | 3022±528 | 0.20 |
| Age [years] | 71.79±9.38 | 69.97±9.55 | 0.07 |
| LH inferior parietal thickness [mm] | 2.4±0.3 | 2.41±0.3 | 0.67 |
| LH cortex volume [mm³] | 197508±24098 | 196550±23693 | 0.98 |
| LH putamen volume [mm³] | 3765±639 | 3687±654 | 0.34 |
| LH frontal pole volume [mm³] | 920±212 | 931.18±205.13 | 0.68 |
| RH retrolenticular part of internal capsule MD [mm²/s] | 8.2⋅10^-4^±3.1⋅10^-4^ | 7.9⋅10^-4^±3⋅10^-4^ | 0.17 |
| LH pars opercularis thickness [mm] | 2.51±0.31 | 2.52±0.3 | 0.91 |
| Pontine crossing tract MD [mm²/s] | 5⋅10^-4^±1.6⋅10^-4^ | 5⋅10^-4^±2⋅10^-4^ | 0.44 |
| GENDER [% of females] | 36% | 37% | 0.91 |
| Splenium of corpus callosum FA | 0.59±0.16 | 0.6±0.15 | 0.40 |
| LH pallidum volume [mm³] | 1721±239 | 1739±243 | 0.64 |
| RH isthmus cingulate thickness [mm] | 2.31±0.26 | 2.33±0.24 | 0.68 |
| LH lateral orbitofrontal volume [mm³] | 7053±1281 | 6954±1278 | 0.47 |
| LH posterior cingulate thickness [mm] | 2.42±0.25 | 2.44±0.29 | 0.62 |
| RH cerebral peduncle MD [mm²/s] | 6.7⋅10^-4^±2.4⋅10^-4^ | 6.7⋅10^-4^±2⋅10^-4^ | 0.67 |
| LH temporal pole thickness [mm] | 3.26±0.57 | 3.15±0.65 | 0.12 |

*Values denote means and standard deviations for continuous variables or percentages for dichotomous variables. P-values were determined with the Kruskal-Wallis test or Chi squared test (α=0.05). Acronyms: CDR, Clinical Dementia Rating scale; LH, Left Hemisphere; RH, Right Hemisphere; FA, Fractional Anisotropy; MD, Mean Diffusivity.*

**Supplementary Table S7:** Variance comparison of the informative features of the entire data set with and without median imputation.

|  | **AD** | **FTD** | **DLB** | **CN** |
| --- | --- | --- | --- | --- |
| **CDR** | 0.73 | 0.95 | 0.66 | 0.81 |
| **LH corticospinal tract FA** | 0.99 | 0.90 | 0.90 | 0.98 |
| **LH superior fronto-occipital fasciculus FA** | 0.86 | 0.77 | 0.79 | 0.99 |
| **RH medial lemniscus MD** | 0.75 | 0.83 | 0.12 | 0.76 |
| **LH entorhinal thickness** | 0.98 | 0.96 | 0.97 | 0.94 |
| **LH hippocampus volume** | 0.97 | 0.99 | 0.99 | 1 |
| **Age** | 1 | 1 | 0.98 | 1 |
| **LH inferior parietal thickness** | 0.98 | 0.98 | 0.95 | 0.94 |
| **LH cortex volume** | 0.98 | 0.99 | 0.96 | 0.98 |
| **LH putamen volume** | 0.98 | 0.99 | 0.99 | 0.92 |
| **LH frontal pole volume** | 0.98 | 0.98 | 1 | 0.98 |
| **RH retrolenticular part of internal capsule MD** | 0.91 | 0.88 | 0.41 | 0.86 |
| **LH pars opercularis thickness** | 0.98 | 0.99 | 0.96 | 0.93 |
| **Pontine crossing tract MD** | 0.61 | 0.92 | 0.47 | 0.79 |
| **GENDER** | 1 | 1 | 0.98 | 1 |
| **Splenium of corpus callosum FA** | 0.9 | 0.87 | 0.88 | 0.91 |
| **LH pallidum volume** | 0.96 | 0.99 | 1 | 0.96 |
| **RH isthmus cingulate thickness** | 0.96 | 1 | 0.98 | 0.99 |
| **LH lateral orbitofrontal volume** | 0.99 | 0.98 | 1 | 0.93 |
| **LH posterior cingulate thickness** | 1 | 0.99 | 0.97 | 1 |
| **RH cerebral peduncle MD** | 0.69 | 0.87 | 0.20 | 0.85 |
| **LH temporal pole thickness** | 0.88 | 0.97 | 0.97 | 0.97 |

*Values denote the p-values determined with the Brown-Forsythe test (α=0.05). Acronyms: CDR, Clinical Dementia Rating scale; LH, Left Hemisphere; RH, Right Hemisphere; FA, Fractional Anisotropy; MD, Mean Diffusivity.*

***References***

- *[1] McKhann, G. et al. Clinical diagnosis of Alzheimer's disease: report of the NINCDS-ADRDA Work Group under the auspices of Department of Health and Human Services Task Force on Alzheimer's Disease. Neurology. 34, 939-44. doi:* [*10.1212/wnl.34.7.939*](https://doi.org/10.1212/wnl.34.7.939) *(1984)*
- *[2] McKhann, G.M. et al. The diagnosis of dementia due to Alzheimer's disease: recommendations from the National Institute on Aging-Alzheimer's Association workgroups on diagnostic guidelines for Alzheimer's disease. Alzheimers Dement. 7, 263-9. doi:* [*10.1016/j.jalz.2011.03.005*](https://doi.org/10.1016/j.jalz.2011.03.005) *(2011)*
- *[3] Montine, T.J. et al. National Institute on Aging-Alzheimer's Association guidelines for the neuropathologic assessment of Alzheimer's disease: a practical approach. Acta Neuropathol. 123, 1-11. doi:* [*10.1007/s00401-011-0910-3*](https://doi.org/10.1007/s00401-011-0910-3) *(2012)*
- *[4] Rascovsky, K. et al. Sensitivity of revised diagnostic criteria for the behavioural variant of frontotemporal dementia. Brain. 134(Pt 9), 2456-77. doi:* [*10.1093/brain/awr179*](https://doi.org/10.1093/brain/awr179) *(2011)*
- *[5] Gorno-Tempini, M.L. et al. Classification of primary progressive aphasia and its variants. Neurology. 76, 1006-14. doi:* [*10.1212/WNL.0b013e31821103e6*](https://doi.org/10.1212/WNL.0b013e31821103e6) *(2011)*
- *[6] McKeith, I.G. et al. Diagnosis and management of dementia with Lewy bodies: third report of the DLB Consortium. Neurology. 65, 1863-72. doi:* [*10.1212/01.wnl.0000187889.17253.b1*](http://https.//doi.org/10.1212/01.wnl.0000187889.17253.b1) *(2005)*
- *[7] McKeith, I.G. et al. Diagnosis and management of dementia with Lewy bodies: Fourth consensus report of the DLB Consortium. Neurology. 89, 88-100. doi:* [*10.1212/WNL.0000000000004058*](https://doi.org/10.1212/WNL.0000000000004058) *(2017)*
- *[8] McKeith, I.G. et al. Consensus guidelines for the clinical and pathologic diagnosis of dementia with Lewy bodies (DLB): report of the consortium on DLB international workshop. Neurology. 47, 1113-24. doi:* [*10.1212/wnl.47.5.1113*](https://doi.org/10.1212/wnl.47.5.1113) *(1996)*
- *[9] Jochems, A.C.C. et al. Contribution of white matter hyperintensities to ventricular enlargement in older adults. Neuroimage Clin. 34, 103019. doi:* [*10.1016/j.nicl.2022.103019*](https://doi.org/10.1016/j.nicl.2022.103019) *(2022)*
- *[10] Orlhac, F. Et al. A Guide to ComBat Harmonization of Imaging Biomarkers in Multicenter Studies. J Nucl Med. 63(2):172-179. doi:* [*10.2967/jnumed.121.262464*](https://doi.org/10.2967/jnumed.121.262464) *(2022)*
